# Proteomics Studies in Gestational Diabetes Mellitus: A Systematic Review and Meta-Analysis

**DOI:** 10.3390/jcm11102737

**Published:** 2022-05-12

**Authors:** Natthida Sriboonvorakul, Jiamiao Hu, Dittakarn Boriboonhirunsarn, Leong Loke Ng, Bee Kang Tan

**Affiliations:** 1 Department of Clinical Tropical Medicine, Faculty of Tropical Medicine, Mahidol University, Bangkok 10400, Thailand; natthida.srn@mahidol.ac.th; 2 Engineering Research Centre of Fujian-Taiwan Special Marine Food Processing and Nutrition, Ministry of Education, Fuzhou 100816, China; jiamiao.hu@fafu.edu.cn; 3 Department of Obstetrics & Gynecology, Faculty of Medicine, Siriraj Hospital, Mahidol University, Bangkok 10700, Thailand; dittakarn.bor@mahidol.ac.th; 4 Department of Cardiovascular Sciences, University of Leicester, Leicester LE1 7RH, UK; lln1@leicester.ac.uk; 5 Diabetes Research Centre, Leicester General Hospital, Leicester LE5 4PW, UK

**Keywords:** gestational diabetes mellitus, pregnancy, proteomics, systematic review, meta-analysis

## Abstract

Gestational Diabetes Mellitus (GDM) is the most common metabolic complication during pregnancy and is associated with serious maternal and fetal complications such as pre-eclampsia and stillbirth. Further, women with GDM have approximately 10 times higher risk of diabetes later in life. Children born to mothers with GDM also face a higher risk of childhood obesity and diabetes later in life. Early prediction/diagnosis of GDM leads to early interventions such as diet and lifestyle, which could mitigate the maternal and fetal complications associated with GDM. However, no biomarkers identified to date have been proven to be effective in the prediction/diagnosis of GDM. Proteomic approaches based on mass spectrometry have been applied in various fields of biomedical research to identify novel biomarkers. Although a number of proteomic studies in GDM now exist, a lack of a comprehensive and up-to-date meta-analysis makes it difficult for researchers to interpret the data in the existing literature. Thus, we undertook a systematic review and meta-analysis on proteomic studies and GDM. We searched MEDLINE, EMBASE, Web of Science and Scopus from inception to January 2022. We searched Medline, Embase, CINHAL and the Cochrane Library, which were searched from inception to February 2021. We included cohort, case-control and observational studies reporting original data investigating the development of GDM compared to a control group. Two independent reviewers selected eligible studies for meta-analysis. Data collection and analyses were performed by two independent reviewers. The PROSPERO registration number is CRD42020185951. Of 120 articles retrieved, 24 studies met the eligibility criteria, comparing a total of 1779 pregnant women (904 GDM and 875 controls). A total of 262 GDM candidate biomarkers (CBs) were identified, with 49 CBs reported in at least two studies. We found 22 highly replicable CBs that were significantly different (nine CBs were upregulated and 12 CBs downregulated) between women with GDM and controls across various proteomic platforms, sample types, blood fractions and time of blood collection and continents. We performed further analyses on blood (plasma/serum) CBs in early pregnancy (first and/or early second trimester) and included studies with more than nine samples (nine studies in total). We found that 11 CBs were significantly upregulated, and 13 CBs significantly downregulated in women with GDM compared to controls. Subsequent pathway analysis using Database for Annotation, Visualization and Integrated Discovery (DAVID) bioinformatics resources found that these CBs were most strongly linked to pathways related to complement and coagulation cascades. Our findings provide important insights and form a strong foundation for future validation studies to establish reliable biomarkers for GDM.

## 1. Introduction

Gestational diabetes mellitus (GDM) is the most common metabolic complication during pregnancy, affecting up to 25% of pregnancies [1]. Women with GDM have higher risks of developing hypertensive disorders during pregnancy, in particular, pre-eclampsia, pre-term birth, cesarean delivery, as well as mental health problems such as anxiety and depression [2,3,4]. Risks to the children include macrosomia (20–30%), small for gestational age (7–10%), shoulder dystocia, neonatal hypoglycemia, neonatal hyperbilirubinemia, respiratory distress syndrome and stillbirth [4]. Further, women with GDM have approximately 10 times higher risk of developing type 2 diabetes mellitus (T2DM) later in life [5,6]. Up to half of the women with GDM develop T2DM within 10 years after delivery [5,6]. Children born to mothers with GDM also face a higher risk of childhood obesity and diabetes later in life [4].

GDM occurs during pregnancy and is characterized by hyperglycemia and insulin resistance, which is partly attributable to the secretion of hormones such as estrogen, progesterone, cortisol and human placental lactogen from the placenta [7,8,9]. In addition, impairment of a compensatory increase in insulin secretion from pancreatic beta-cells contributes to the development of GDM [10]. This dysfunction can be caused by either an autoimmune process (a state of chronic insulin resistance) or a genetic abnormality leading to abnormalities of insulin secretion [11].

There are several screening/diagnostic criteria for GDM, such as the International Association of the Diabetes and Pregnancy Study Groups [12] and the American Diabetes Association criteria [13]. Various combinations of risk factors have been used for selective screening and diagnosis of GDM [14]. Risk factors for GDM include a high maternal body mass index (BMI), maternal age, previous GDM, previous macrosomic baby, family history of diabetes (specifically, first-degree relatives) and ethnicity (such as South Asian women) [15,16]. Screening is usually performed using the Oral Glucose Tolerance Test (OGTT) at 24–28 weeks of gestation (the second trimester), leaving only limited time for interventions/treatments.

Recently, research into biomarkers for early and accurate prediction of GDM has increased. Early prediction/diagnosis lends itself to early interventions such as diet and lifestyle, which could mitigate the maternal and fetal complications associated with GDM [17]. Adipokines, especially adiponectin, have received particular attention [18]. Adiponectin has insulin-sensitizing and protective effects on vascular endothelial cells [19]. Other adipokines related to adiponectin are also being studied in the context of diabetes mellitus [20], such as C1QTNF-related protein-1 (CTRP-1). A recent publication reported that CTRP-1 is linked to insulin resistance in pregnancy and could be a metabolic biomarker for insulin resistance in women with GDM [21]. Other adipokines that have been related to GDM include chemerin (new Ref. [1]) and retinol-binding protein-4 [22]. In addition, maternal lipids, in particular triglycerides, are associated with GDM [23]. Nevertheless, no biomarkers identified to date have been proven to be effective in the prediction/diagnosis of GDM, including combining maternal risk factors [24,25,26,27,28,29,30,31].

The proteome is an expression of a set of proteins in a given time and space with varied compositions in different cells, tissues or biological fluids, which is commonly studied in untargeted biomarker discovery [32]. Proteomic approaches based on mass spectrometry (MS) have been applied in various fields of biomedical research to identify novel biomarkers using platforms such as liquid-chromatography tandem MS using Data Dependent Acquisition (DDA) or Data Independent Acquisition (DIA) [33]. Aptamer-based proteomics have also been used in biomarker discovery studies [34]. Although a number of proteomic studies in GDM now exist, a lack of a comprehensive and up-to-date meta-analysis makes it difficult for researchers to interpret the data in the existing literature. To the best of our knowledge, this is the first meta-analysis performed on this topic.

## 2. Material and Methods

The aim of this systematic review was to identify proteomic studies using an untargeted discovery approach (to avoid bias) in women with and without GDM to assess if levels differed between the two groups. In addition, we also assessed the replicability and regulation of candidate biomarkers (CB) for GDM in terms of study cohorts, sample types, blood fractions and blood collection time, proteomic platform and continent.

### 2.1. Search Strategy

This systematic review is reported following the Preferred Reporting Items for Systematic Reviews and Meta-Analyses (PRISMA) [35]. We searched the following databases: MEDLINE, EMBASE, Web of Science and Scopus from their inception to January 2022. The search strategy for each database was developed using the following terms: “Proteomics” and “Gestational diabetes mellitus”. Keywords for Gestational diabetes mellitus: “Gestational Diabetes” or “Diabetes Mellitus Gestational” or “Pregnancy-Induced Diabetes” or “GDM”, and Keywords for Proteomics analysis: “Proteomics” or “Proteomic” and “Mass Spectrometry”. In addition, hand-searching the reference list for eligible studies and direct contact with authors, when necessary. The final search was carried out on 13 December 2021. We limited our search to articles written in English but did not place any restrictions on publication date. The protocol was registered with the International Prospective Register for Systematic Reviews (PROSPERO) database: registration number CRD42020185951.

### 2.2. Study Selection

Two reviewers (N.S., J.H.) independently screened the title and abstracts of all papers and, according to their relevance, obtained full-text reports for further scrutiny, and an agreement was reached on final inclusions. In the event of any disagreement, a third reviewer (B.K.T) was involved in resolving any disagreements. The inclusion criteria for this systematic review were: (1) proteomic studies; (2) mass spectrometry analysis; (3) cohort, case-control and observational studies reporting original data; (4) articles published in the English language; (5) studies performed on human participants; (6) studies performed on pregnant women; (7) studies with a control group. The exclusion criteria were: (1) other types of diabetes apart from GDM; (2) conference abstracts, case reports, case series, letters, editorials, guidelines, theses, commentaries, reviews, systematic reviews, brief communication or technical note; (3) reported associations without any retrievable data. Search results were managed in Endnote.

Any disagreement during the process was resolved through discussion and, when necessary, by the advice of a third reviewer (B.K.T.). Any discrepancies were resolved through discussion or with the advice of a third reviewer (B.K.T.).

### 2.3. Data Extraction and Risk of Bias Assessment

Data were extracted independently by two reviewers (N.S., J.H.) following the Cochrane Handbook guidelines [36], and findings were reported according to PRISMA guidelines [35]. Any discrepancies were resolved through discussion or with the advice of a third reviewer (B.K.T.). The risk of bias in each study was assessed using the Newcastle–Ottawa Scale by the two reviewers (N.S. and J.H.) independently. We judged studies that received a score of nine or eight stars to be at low risk of bias, studies that scored seven or six stars to be at medium risk, and those that scored five or less to be at high risk. The articles included in our meta-analysis are presented in Supplementary Table S1; for information that was not reported in either the manuscript or accompanying Supplementary Materials, we contacted the corresponding authors of these manuscripts to acquire the information.

### 2.4. Data Analysis

#### 2.4.1. Replicability of Candidate Biomarkers (CBs)

The replicability of a CB was assessed according to the number of cohorts that were reported. The replicable CBs were grouped by sample types, blood fraction and time of blood collection, proteomic platform and continent, given the influence these analytical and demographic factors have on protein quantification and expression. The various proteomic strategies that were utilized for CB discovery were categorized according to the mass spectrometry approach, including gel image and spectra intensity (label-free and chemical labeling). In addition, the replicable CBs should be grouped by ethnicity as this could influence the expression of CBs. However, there was only one cohort with data on ethnicity (data not shown). Given most studies reported only the county of origin, the replicability of CBs was grouped by continents instead. The discovery of similar CBs across different cohorts would point to a potentially valuable GDM biomarker if the regulation was consistent. In this systematic review, a CB would be considered if reported consistently across all cohorts.

#### 2.4.2. Meta-Analysis

Studies reporting CB with fold change and p-value were included and calculated using the generic inverse variance method in RevMan 5 to obtain the ratio of mean values (RoM) and 95% confidence intervals (CI). For studies that did not report the exact *p*-value, e.g., *p* < 0.05, we set the *p*-value equal to 0.05 to perform calculations. Pooled analyses were performed amongst 40 CBs that were reported in at least 2 studies (Alpha-1-antitrypsin, Annexin A4, Apolipoprotein A-V, Apolipoprotein C-III, Apolipoprotein E, Apolipoprotein M, C4b-binding protein alpha chain, Coagulation factor IX, Coagulation factor V, Coagulation factor X, Coagulation factor XII, Complement C1s subcomponent, Complement component C6, Complement component C7, Complement component C8 beta chain, Complement component C8 gamma chain, Complement component C9, Complement factor B, Complement factor H, C-reactive protein, Endoplasmin, Fibrinogen alpha chain, Fibrinogen beta chain, Fibrinogen gamma chain, Gelsolin, Glyceraldehyde-3-phosphate dehydrogenase, Ig mu chain C region, IGL@ protein, Insulin-like growth factor-binding protein 5, Mannan-binding lectin serine protease 2, Pappalysin-1 (also known as pregnancy-associated plasma protein A (PAPP-A), Plasma protease C1 inhibitor, Pregnancy zone protein, Proteoglycan 4, Prothrombin, Retinol-binding protein 4, Secreted phosphoprotein 24, Serum amyloid *p*-component, Sex hormone-binding globulin and Serum paraoxonase/arylesterase 1). Outcome measures were presented as a ratio of mean (RoM) between GDM and controls with 95% confidence intervals (CI). Between-study heterogeneity was assessed by the Higgins I2 statistic, which provides an estimate of the percentage of variability across studies that is due to heterogeneity rather than chance alone [37]. The random-effect model was used given the variability of CB measurements between different laboratories and proteomic platforms. An RoM of more than 1 would indicate upregulation of GDM CBs, and an RoM of less than 1 would indicate downregulation. A similar approach was used when we performed further analyses on blood (plasma/serum) CBs in early pregnancy (first and/or early second trimester).

#### 2.4.3. Enrichment Analysis

The enrichment analysis was conducted on consistently regulated CBs to identify the most enhanced pathway in which a CB is consistently involved and in a replicable manner. The analysis was performed using the Database for Annotation, Visualization and Integrated Discovery (DAVID) bioinformatics resources. DAVID is an integrated biological database that extracts meaningful information from large lists of proteins [38]. UniProt accession numbers were used as the identifier in DAVID. The importance of enriched pathways was ranked in accordance with the modified Fisher’s exact test (EASE score) [39].

## 3. Results

### 3.1. Literature Search Results and Characteristics of Included Studies

**Figure 1:**
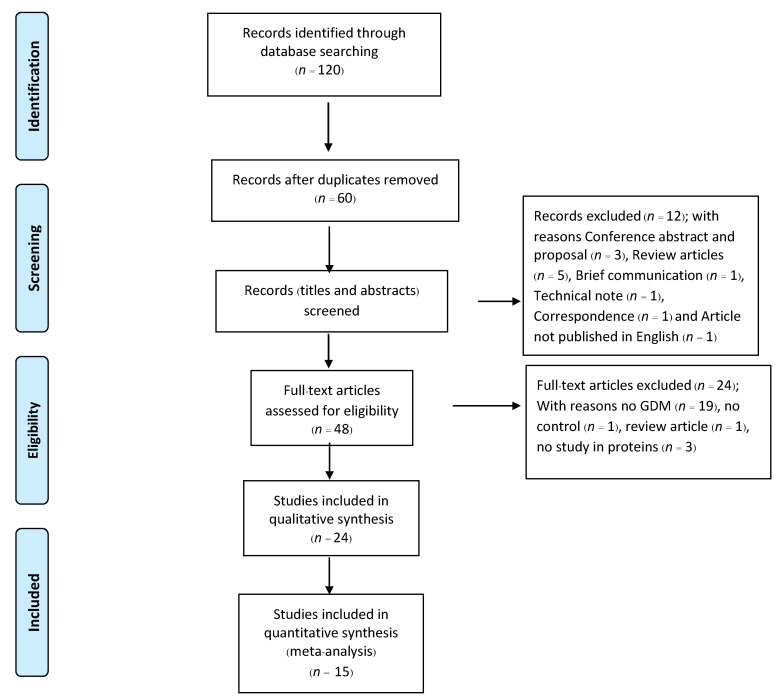
Preferred Reporting Items for Systematic Reviews and Meta-Analyses (PRISMA) chart of study selection for systematic review and meta-analysis.

The results from our literature search are summarized in a PRISMA flowchart in Figure 1. A total of 120 articles were identified through MEDLINE, EMBASE, Web of Science and Scopus. After the removal of duplicates, 60 articles remained and were screened based on titles and abstracts. Only 48 full-text articles were relevant and assessed for eligibility based on the pre-specified inclusion criteria. Finally, a total of 24 studies were included in this systematic review, 15 of which had adopted the case-control approach, 9 were with both case-control and longitudinal study designs. Characteristics of the studies included in the meta-analyses are presented in Supplementary Table S1. Information about how the diagnosis of GDM is made by each study is presented in Supplementary Table S2.

For case-control studies, the reported CBs were determined amongst 687 pregnant women with GDM and 656 controls. For longitudinal studies, 217 GDM and 219 controls were involved. The 24 included studies were conducted in the following continents, i.e., Asia (*n* = 14), Europe (*n* = 6), North America (*n* = 3) and Australia (*n* = 1). Whilst 11 studies utilized plasma samples, 7 studies utilized serum, and 6 studies utilized other samples, i.e., urine (*n* = 1), amniotic fluid (*n* = 1), rectus abdominus skeletal muscle tissue (*n* = 1), placenta villi (*n* = 1), omental adipose tissue (*n* = 1) and urine exosomes (*n* = 1). Furthermore, 11 studies performed sample collection for proteomic analyses during the second trimester of pregnancy, 4 studies during the first trimester of pregnancy, 1 study during the first and second trimesters of pregnancy, 1 study during the second and third trimesters of pregnancy, and 7 studies did not provide data on when the samples were collected.

### 3.2. Quality and Risk of Bias Assessment

Supplementary Table S3 shows the quality assessment using the Newcastle–Ottawa Scale (NOS) for all included studies. All 24 included studies showed good quality and low risk of bias, with four stars in the selection domain, one or two stars in the comparability domain, and three stars in the outcome/exposure domain (Total NOS score 8 or 9). Therefore, all 24 studies were included in the qualitative synthesis. A total of 15 studies were included in the meta-analysis.

### 3.3. Replicability of CBs

The replicability of CBs was assessed across different cohorts. Amongst the 24 included studies, two studies shared the same cohort after clarification from the authors [40,41]. The total cohorts were thus reduced to 23 independent cohorts [42,43,44,45,46,47,48,49,50,51,52,53,54,55,56,57,58,59,60,61,62].

Supplementary Table S4 shows the replicability of 262 CBs across the 23 independent cohorts. The CBs were significantly and differentially expressed in women with GDM compared to controls. Approximately 19% of CBs (49 out of a total 262 CBs) were reported in at least two different cohorts. Fibrinogen alpha chain was reported in 10 independent cohorts [46,47,48,49,50,51,52,53,54,55]. A total of 15 CBs were reported across three cohorts, 33 CBs were reported across two cohorts, and the remaining CBs were only discovered in a single cohort (Supplementary Table S4).

Of the 49 replicable CBs, a total of 14 CBs were found to be consistently upregulated and associated with complement and coagulation cascades, peroxisome proliferator-activated receptors signaling pathway, immune and inflammatory responses, glycolytic pathway, cardiovascular disease, autoimmune disorders, malignancy and autoimmune disease. On the other hand, 16 CBs were found to be consistently downregulated and known to be involved in complement and coagulation cascades, prion diseases, systematic lupus erythematosus and amoebiasis. The regulation of 19 CBs was found to be inconsistent across the relevant studies and associated with complement and coagulation cascades, platelet activation and staphylococcus aureus infection. Interestingly, all three types of replicable CBs (consistent upregulation, consistent downregulation and inconsistent regulation) were involved in complement and coagulation cascades.

#### 3.3.1. Grouping of Replicable CBs by Sample Type

Supplementary Table S5 shows the grouping of replicable CBs with consistent regulation by sample type (*n* = 30). Plasma/sera samples were obtained from peripheral vessels. In addition, plasma samples were obtained from umbilical vessels. All CBs were found in both plasma and/or serum. The serum is derived from blood but without clotting factors, containing proteins such as albumin, immunoglobulins, transferrin, haptoglobin and lipoproteins [63]. Analyses of CBs in serum/plasma samples frequently require separation of their components, usually, albumin depletion as a fraction of interest. Such “preprocessing” of serum/plasma specimens is very important in proteomic analyses based on mass spectrometry as high-abundant proteins such as albumin suppress peptide ions originating from low-abundant proteins, limiting the probability and reliability of their detection [64]. We found that PAPP-A was found in plasma but not in sera. It is an enzyme that cleaves insulin-like growth factor-binding protein 4 and insulin-like growth factor-binding protein 5, releasing insulin growth factor (IGF). IGF then binds to IGF receptors, resulting in activation of the IGF pathway. This protein plays an important role in bone formation, inflammation, wound healing and female fertility [65]. Furthermore, haptoglobin was found in both sera and urine exosomes but not in plasma. Through hemolysis, hemoglobin is found to accumulate in the kidneys and is secreted in the urine. Haptoglobin combines with free plasma hemoglobin to allow hepatic recycling of heme iron and to prevent renal damage. Haptoglobin acts as an antioxidant, has antibacterial activity and plays a role in modulating many aspects of the acute phase response. Hemoglobin/haptoglobin complexes are rapidly cleared by the macrophage CD163 scavenger receptor expressed on the surface of liver Kupfer cells through an endocytic lysosomal degradation pathway [66]. Replicable CBs were identified more in sera compared to plasma even though 11 studies utilized plasma whereas 7 studies utilized sera.

#### 3.3.2. Grouping of Replicable CBs by Blood fraction and Collection Time

Table 1 shows the grouping of replicable CBs with consistent regulation by blood fraction and collection time. All replicable plasma CBs were collected during the second trimester of pregnancy for proteomic analyses whilst replicable sera CBs were collected in either the first or the second trimester of pregnancy. One study did not report collection time [55]. We found that C-reactive protein (CRP), Ig mu chain C region (IGHM), Proteoglycan 4 (PRG4), Secreted phosphoprotein 24 (SPP24), Sex hormone-binding globulin (SHBG), Coagulation factor V (F5), complement component C9 (C9), Alpha-1-antitrypsin, Apolipoprotein C-III (APOC3) and Serum amyloid *p*-component (SAP) were identified only in second-trimester plasma samples and only in first trimester sera. Plasma and sera are important specimens for protein biomarker discovery as it is easily collected and contain highly abundant proteins secreted into the blood circulation by normal or damaged cells in both health and disease [67]. Several protein concentrations could be measured in plasma and/or sera in routine clinical practice. The dynamic concentration range of proteins such as albumin could achieve at least 10 orders of magnitude [68].

#### 3.3.3. Grouping of Replicable CBs by Proteomic Platform

Supplementary Table S6 shows the grouping of replicable CBs with consistent regulation by mass spectrometry proteomic platform. CRP, IGHM, PRG4, Secreted phosphoprotein 24, SHBG, APOC3, Haptoglobin, PAPP-A and SAP components were analyzed using both label-free and chemical labeling techniques. The label-free approach can identify more proteins and cover a broader range of protein expression levels; however, the chemical labeling approach is more accurate [69]. iTRAQ (isobaric tagging for relative and absolute quantification) and TMT (tandem mass tags) are chemical labels that have been used in a wide range of different clinically orientated plasma and sera proteomics studies [70]. We found that APOC3 was analyzed using all proteomic platforms (including gel image, label-free and chemical labeling). APOC3 is a small apolipoprotein of 79 amino acid residues [71]. In the circulation, APOC3 is mainly present on triglyceride (TG)-rich lipoproteins (TRLs) and high-density lipoprotein (HDL) and on low-density lipoprotein (LDL) particles [72]. APOC3 controls lipid metabolism in multiple ways, including the inhibition of lipoprotein lipolysis and receptor-mediated uptake of TRLs as well as stimulating the production of very-low-density lipoproteins (VLDLs) [73].

#### 3.3.4. Grouping of Replicable CBs by Continent

Supplementary Table S7 shows the grouping of replicable CBs with consistent regulation by continent. Most CBs were found in Asian-based studies. A total of 14 out of 24 included studies (58%) studies were conducted in Asia. PAPP-A was identified in Australian and Asian-based studies. SPP24, SHBG, Antithrombin-III and SAP were identified in European and Asian-based studies. Haptoglobin was identified in American and European-based studies.

#### 3.3.5. Consistent Pattern from 19 Inconsistent CBs

We performed further analyses of the 19 inconsistent CBs, including Annexin A4, Apolipoprotein A-IV, Apolipoprotein M, Complement factor B, F5, Extracellular matrix protein 1, Fibrinogen alpha chain (FGA), Fibrinogen beta chain, Fibrinogen gamma chain, IGL@ protein, Mannan-binding lectin serine protease 2, Phospholipid transfer protein, Plasma protease C1 inhibitor, Pregnancy zone protein, Prothrombin (P), Retinol-binding protein 4, Serum amyloid A-2 protein, Transthyretin and Serum paraoxonase/arylesterase 1 (PON1) to find consistent patterns (up or downregulation) according to sample type, sample collection time, proteomic platform and continent. We found that in Asian studies, there were three CBs (F5, Fibrinogen gamma chain and PON1), which were consistent according to sample type and proteomic platform. Using the chemical labeling proteomic platform, PON1 was consistently upregulated, whereas serum F5 and plasma Fibrinogen gamma chain were consistently downregulated.

### 3.4. Meta-Analysis

Supplementary Figures S1–S40 show the forest plots of the 40 CBs in women with GDM compared to controls. We found that 9 CBs were significantly upregulated in women with GDM compared to controls, i.e., Apolipoprotein A-V (APOA5), APOC3, Apolipoprotein E (APOE), Coagulation factor IX (F9), Coagulation factor X (F10), Coagulation factor XII (F12), Complement C1s subcomponent (C1S), PRG4 and SAP (Supplementary Figures S1–S9) and 13 CBs were found to be significantly downregulated in women with GDM compared to controls, i.e., C4b-binding protein alpha chain (C4BPA), Complement component C6 (C6), Complement component C7 (C7), Complement component C8 beta chain (C8B), Complement component C8 gamma chain (C8G), C9, Complement factor H (CFH), Endoplasmin (EPN), Gelsolin (GSN), IGHM, PAPP-A, SPP24 and SHBG (Supplementary Figures S10–S22); however, the remaining CBs were not significant in women with GDM compared to controls.

We performed further analyses on blood (plasma/serum) CBs in early pregnancy (first and/or early second trimester). Moreover, we excluded studies with less than 10 samples (see Supplementary Table S1—studies 3, 11, 12, 16, 17, 19). Hence, there were nine studies included in total for these further analyses (see Supplementary Table S1—studies 4, 7, 9, 15, 20, 21, 22, 23, 24). Supplementary Figures S41–S44 show the forest plots of the 27 CBs in women with GDM compared to controls. We found that 11 CBs were significantly upregulated in women with GDM compared to controls, i.e., APOA5, APOE, F9, F10, F12, C1S, FGA, IGF binding protein 5 (IGFBP-5), PRG4, SAP and PON1 and 13 CBs were found to be significantly downregulated in women with GDM compared to controls, i.e., were C4BPA, F5, C6, C7, C8B, C8G, C9, CFH, EPN, GSN, IGHM, P and SPP24; the remaining CBs were not significant in women with GDM compared to controls.

### 3.5. Pathway Analysis for Replicable CBs

Supplementary Table S8 shows the DAVID functional annotation chart analyses representing over-represented pathways in KEGG. We found that pathways related to complement and coagulation cascades (*p* = 1.5 × 10^−19^), systemic lupus erythematosus (*p* = 4.2 × 10^−4^) and prion diseases (*p* = 3.3 × 10^−5^) were significant. However, amoebiasis (*p* = 5.1 × 10^−1^) was not significant.

Further pathway analysis for replicable CBs pertaining to early pregnancy blood CBs for GDM (27 CBs) is presented in Supplementary Table S9. We found that pathways related to complement and coagulation cascades (*p* = 4.7 × 10^−22^), coronavirus disease COVID-19 (*p* = 6.3 × 10^−6^), system lupus erythematosus (*p* = 1.9 × 10^−4^), prion diseases (*p* = 5.9 × 10^−2^) and amoebiasis (*p* = 4.2 × 10^−1^) was not significant.

## 4. Discussion

The current study presents a systematic review and meta-analysis showing highly replicable CBs that can differentiate between women with and without GDM across various proteomic platforms, sample types, blood fractions and time of blood collection and continents. We found that 22 CBs were significantly different between women with GDM and controls, of which 9 CBs were upregulated and 13 CBs downregulated. The nine CBs that were upregulated were APOA5, APOC3, APOE, F9, F10, F12, C1S, PRG4 and SAP. Of these nine CBs, three CBs, i.e., APOC3, APOE and PRG4, were replicated with consistent regulation in at least three independent cohorts. On the other hand, the 13 CBs that were downregulated were C6, C7, C8B, C8G, C9, CFH, C4BPA, EPN, GSN, IGHM, PAPPA, SPP24 and SHBG. Moreover, 5 of these 13 CBs, i.e., C9, GSN, IGHM, SPP2 and SHBG, were replicated with consistent regulation in at least three independent cohorts. Subsequent pathway analysis using DAVID found that these CBs were linked to pathways related to complement and coagulation cascades, system lupus erythematosus and prion diseases.

In a bid to focus on the most clinically useful CBs for GDM, we performed further analyses on blood (plasma/serum) CBs in early pregnancy (first and/or early second trimester). Blood (plasma/serum) would be most likely to be used for routine early GDM screening. Moreover, the ability to predict/diagnose GDM early would allow interventions such as diet and lifestyle to be implemented in a timely manner, which could further lessen the maternal and fetal complications associated with GDM. Moreover, we included studies with more than nine samples. Hence, there were nine studies included in total for these further analyses (detailed in the Results section). We found that 24 CBs were significantly different between women with GDM and controls, of which 11 CBs were upregulated and 13 CBs downregulated. The 11 CBs that were upregulated were APOA5, APOE, F9, F10, F12, C1S, FGA, IGFBP-5, PRG4, SAP and PON1. Conversely, the 13 CBs that were downregulated were C4BPA, F5, C6, C7, C8B, C8G, C9, CFH, EPN, GSN, IGHM, P and SPP24. In addition, of these 24 CBs, 3 CBs, i.e., FGA, IGHM and PRG4, were replicated with consistent regulation in at least three independent cohorts. Subsequent pathway analysis using DAVID found that these CBs were linked to pathways related to complement and coagulation cascades, coronavirus disease COVID-19, system lupus erythematosus and prion diseases.

In the early pregnancy blood CBs for GDM, the twp Apolipoprotein CBs that were significantly upregulated in women with GDM were APOA5 and APOE. APOA5 plays an important role in the pathophysiology of insulin resistance-related hypertriglyceridemia; obese human participants had lower plasma APOA5 levels, which were inversely correlated with TGs, body mass index and homeostasis model assessment of insulin resistance (HOMA-IR) [74]. APOE promotes the clearance of VLDL and LDL from the blood circulation and is involved in the reverse transport of cholesterol [75]. A study reported that APOE protein levels are decreased in serum samples from patients with T2DM [76]. Interestingly, a recent study showed that APOE was down-regulated in the serum and placenta of women with GDM; however, this was a small study involving a total of 50 pregnant women [77].

There were four Coagulation factor CBs that were found to be significantly different in women with GDM in the early pregnancy blood CBs for GDM, i.e., (F9, F10 and F12 were upregulated whilst F5 was down-regulated. Coagulation factors are blood proteins that are involved in hemostasis. During pregnancy, the concentrations of F5, F7, F8, F9, F10, F12 and von Willebrand factor increase significantly together with an increase in the concentration of plasma fibrinogen [78]. This helps blood clot more easily during pregnancy to reduce blood loss during labor and delivery [79]. Further, F12 is intrinsically related to coagulation in normal pregnancy and is also a marker of GDM [80].

The remaining six CBs that were significantly upregulated amongst the early pregnancy blood CBs for GDM were C1S, FGA, IGFBP-5, PRG4, SAP and PON1. C1S expression was found to be upregulated in human adipocytes from obese, insulin-resistant study participants [81]. FGA is one of the main components of blood clots, playing a key role in haemostasis and thrombosis [82]. Changes in the IGF system were implicated in the development of GDM, glucose homeostasis and fetal growth [83]. Our finding of an upregulation of IGFBP-5 sheds new light on the relationship between the IGF axis and GDM. PRG4 is a proteoglycan that acts as a joint/boundary lubricant [84]. Of interest, a study showed that hyperglycemia altered proteoglycan expression and the glycosaminoglycan composition in the placentas of women with GDM [85]. Amongst acute-phase inflammation-induced proteins, SAP is primarily produced by hepatocytes and mainly in response to pro-inflammatory cytokines [86]. Further, SAP suppresses the progression of diabetic nephropathy by reducing the secretion of chemokine C-C motif ligand 1 by macrophages [87]. Human serum PON1 is associated with HDLs, and PON1 is one of the few modifying transfer proteins that interact with serum lipoproteins [88].

In the early pregnancy blood CBs for GDM, there were seven Complement CBs that were downregulated in women with GDM, i.e., C6, C7, C8B, C8G, C9, CFH and C4BPA. The complement system plays an important role in the initiation and maintenance of inflammation, and its activation is launched via three different pathways: the classic pathway, the alternative pathway and the lectin pathway [89]. C6, C7, C8B, C8G and C9 are the terminal complement components that are necessary to form the membrane attack complex (the main effector of complement-mediated tissue damage) and are constitutively present in plasma [90]. In addition, CFH is a serum glycoprotein that expedites the decay of C3 convertase and is a cofactor for the inactivation of C3b [91]. GDM is a pro-inflammatory state as evidenced by raised C-reactive protein (CRP) levels; increased oxidative stress is found in GDM, secondary to insulin resistance, leading to lower CFH and higher C3 levels [92]. A recent study reported that the elevated complement factors (C3, C4 and CFH) in women with GDM could be mainly accounted for by inflammation [93]. C4BP is a polymer of seven identical alpha chains (C4BPA) and one unique beta chain secreted from the pancreas; C4BP inhibits islet amyloid polypeptide-mediated inflammasome activation and secretion of the diabetogenic cytokine Interleukin 1 beta [94].

The remaining five CBs that were significantly downregulated in women with GDM were EPN, GSN, IGHM, P and SPP24. EPN is a luminal protein of the endoplasmic reticulum (ER) [95]. In T2DM, ER stress-induced dysfunction of pancreatic beta cells decreases insulin production and its bioactivity leading to hyperglycemia [96]. GDM is a pre-diabetic state; though there could be various underlying causes, most cases are characterized by low-grade chronic beta-cell dysfunction [97]. GSN was reported to modulate insulin secretion from pancreatic β-cells by remodeling the actin cytoskeleton [98] and stimulating insulin secretion by depolymerizing F-actin in β-cells [99]. Furthermore, GSN was able to reduce blood glucose levels, and plasma GSN levels were lower in T2DM [100]. IGHM is a constant region of immunoglobulin heavy chains; immunoglobulins or antibodies are antigen receptors expressed by B cells and secreted by plasma cells and represent one of the major components of the adaptive immune response [101]. IGHM levels were reported to be significantly downregulated in women with GDM [53,54], and importantly, low IGHM levels could predict women who subsequently developed GDM [51]. P is a plasma protein that is converted to thrombin and promotes blood coagulation. A recent report showed that the prothrombin time was significantly lower in women with GDM and related to the severity of GDM [102]. Finally, SPP24, primarily synthesized by the liver, is a cytokine-binding bone matrix protein with several truncated C-terminal products [103]. Moreover, serum SPP24 levels have been reported to be inversely correlated with the estimated Glomerular Filtration Rate (eGFR), a measure of kidney function [103].

The strengths of this systematic review and meta-analysis are that robust methods have been utilized throughout, including a comprehensive search of multiple databases; publication bias and study quality have both been assessed, and meta-analysis methodologies have been used to pool results across studies. We also employed enrichment analyses on consistently regulated CBs to identify the most enhanced pathway in which a CB is consistently involved and in a replicable manner using the Database for Annotation, Visualization and Integrated Discovery (DAVID) bioinformatics resources, which extracts meaningful information from large lists of proteins. Our systematic review and meta-analysis provide a more precise estimate of the effect size and increase the generalizability of the results of all individual studies. This enhances the precision of our assessment of CBs for the prediction of GDM, thus, providing important insights into the discovery of CBs to predict GDM using a proteomics approach. A limitation of this study is that we do not have the individual patient data for the included studies and are thus unable to perform a predictive model AUC (area under the curve) for data from early pregnancies. A further limitation would be that discovery-based studies have high inter-study variation, which could result in an inconclusive accentuation of biomarkers. However, the random effect model was selected in this systematic review and meta-analysis to reduce the variability of CB measurements between different laboratories and proteomic platforms. Furthermore, our study did not detect adiponectin, a fat-derived hormone with insulin-sensitizing and anti-inflammatory properties, the levels of which were found to be low in women with GDM [104]. Adiponectin is arguably the best-studied biomarker for GDM. This limitation could be related to the proteomic approaches employed by the researchers in the included studies. In order to elaborate, quantitation in plasma-based proteomics requires the reproducible removal of highly abundant proteins to allow the less abundant proteins to be detectable by the mass spectrometer (MS) [105]. There are two generally utilized methods, i.e., immune-depletion and enrichment [106]. Immuno-depletion involves the retention of proteins in columns using antibodies that have an affinity for preselected abundant proteins [105]. Enrichment is an alternative to depletion, and this technique involves protein separation by affinity chromatography [105]. However, a key challenge is the large dynamic range of protein concentrations in plasma [107]. A recent report has shown that the dynamic range of a typical MS platform is not compatible with the dynamic range of plasma protein concentrations, and this limits the ability of the MS to detect low-abundance proteins [106].

## 5. Conclusions

GDM is the most common metabolic complication during pregnancy and is associated with serious maternal and fetal complications such as pre-eclampsia and stillbirth. Further, women with GDM have approximately 10 times higher risk of T2DM later in life. Children born to mothers with GDM also face a higher risk of childhood obesity and diabetes later in life. To the best of our knowledge, this is the most comprehensive and up-to-date systematic review and the only meta-analysis with high-quality data on this topic.

This study identified 22 highly replicable CBs that were significantly different (9 CBs were upregulated and 12 CBs downregulated) between women with GDM and controls across various proteomic platforms, sample types, blood fractions and time of blood collection and continents. We performed further analyses on blood (plasma/serum) CBs in early pregnancy (first and/or early second trimester) and included studies with more than nine samples (nine studies in total). We found that 11 CBs were significantly upregulated, and 13 CBs significantly downregulated in women with GDM compared to controls. Subsequent pathway analysis using Database for Annotation, Visualization and Integrated Discovery (DAVID) bioinformatics resources found that these CBs were most strongly linked to pathways related to complement and coagulation cascades. Our findings provide important insights and form a strong foundation for future validation studies to establish reliable biomarkers for GDM.

## Figures and Tables

**Table 1:** Grouping of replicable candidate biomarkers (CBs) by blood fraction and collection time.

Replicable CB	Regulation	Total Cohort	Plasma (Number of Cohort)			Serum (Number of Cohort)			Reference (First Name, Year)		
			Sample Collection for Proteomics Analysis (Trimester)			Sample Collection for Proteomics Analysis (Trimester)					
			1st	1st–2nd	2nd	1st	1st–2nd	2nd			
C-reactive protein	up	3			2	1			Liu, X. 2020 [54]	Shen, L. 2019 [53]	Zhao, C. 2015 [49]
Ig mu chain C region (Immunoglobulin heavy constant mu)	down	3			2	1			Liu, X. 2020 [54]	Shen, L. 2019 [53]	Zhao, D. 2017 [51]
Proteoglycan 4	up	3			2	1			Liu, X. 2020 [54]	Shen, L. 2019 [53]	Zhao, D. 2017 [51]
Secreted phosphoprotein 24	down	3			1	2			Liu, X. 2020 [54]	Ravnsborg, T. 2019 [41]	Shen, L. 2019 [53]
Sex hormone-binding globulin	down	3			2	1			Liu, X. 2020 [54]	Ravnsborg, T. 2016 and 2019 [40,41]	Zhao, C. 2015 [49]
Coagulation factor V	down	2			1	1			Shen, L. 2019 [53]	Zhao, D. 2017 [51]	
Complement component C9	down	2			1	1			Shen, L. 2019 [53]	Zhao, D. 2017 [51]	
Alpha-1-antitrypsin	down	2			1	1			Shen, L. 2019 [53]	Zhao, C. 2015 [49]	
Antithrombin-III	down	2				1		1	Ravnsborg, T. 2019 [41]	Zhao, D. 2017 [51]	
Apolipoprotein A-V	up	2				1		1	Shen, L. 2019 [53]	Zhao, D. 2017 [51]	
Apolipoprotein C-III	up	2			1	1			Kim, S.M. 2012 [43]	Shen, L. 2019 [53]	
Apolipoprotein E	up	2				1		1	Shen, L. 2019 [53]	Zhao, D. 2017 [51]	
C4b-binding protein alpha chain	down	2				1		1	Shen, L. 2019 [53]	Zhao, D. 2017 [51]	
Coagulation factor IX	up	2				1		1	Shen, L. 2019 [53]	Zhao, D. 2017 [51]	
Coagulation factor X	up	2				1		1	Shen, L. 2019 [53]	Zhao, D. 2017 [51]	
Coagulation factor XII	up	2				1		1	Shen, L. 2019 [53]	Zhao, D. 2017 [51]	
Complement C1s subcomponent	up	2				1		1	Shen, L. 2019 [53]	Zhao, D. 2017 [51]	
Complement component C6	down	2				1		1	Shen, L. 2019 [53]	Zhao, D. 2017 [51]	
Complement component C7	down	2				1		1	Shen, L. 2019 [53]	Zhao, D. 2017 [51]	
Complement component C8 beta chain	down	2				1		1	Shen, L. 2019 [53]	Zhao, D. 2017 [51]	
Complement component C8 gamma chain	down	2				1		1	Shen, L. 2019 [53]	Zhao, D. 2017 [51]	
Complement factor H	down	2				1		1	Shen, L. 2019 [53]	Zhao, D. 2017 [51]	
Endoplasmin	down	2				1		1	Shen, L. 2019 [53]	Zhao, D. 2017 [51]	
Gelsolin	down	2				1		1	Shen, L. 2019 [53]	Zhao, D. 2017 [51]	
Glyceraldehyde-3-phosphate dehydrogenase	up	2				1		1	Shen, L. 2019 [53]	Zhao, D. 2017 [51]	
Insulin-like growth factor-binding protein 5	up	2				1		1	Shen, L. 2019 [53]	Zhao, D. 2017 [51]	
Pappalysin-1	down	2			2				Jayabalan, N. 2019 [59]	Zhao, C. 2015 [49]	
Serum amyloid *p*-component	up	2			1	1			Liu, X. 2020 [54]	Ravnsborg, T. 2019 [41]	
Serum paraoxonase/arylesterase 1	up	2				1		1	Shen, L. 2019 [53]	Zhao, D. 2017 [51]	

## Data Availability

All data underlying this article are available in the article and in its online Supplementary Material. We will willingly share our knowledge, protocol and expertise when asked.

## Supplementary Materials

The following supporting information can be downloaded at: https://www.mdpi.com/article/10.3390/jcm11102737/s1, Supplementary Figures—Supplementary Figures S1–S40 show the forest plots of the 40 CBs in women with GDM compared to controls; Figure S41—Forest plot for Serum amyloid P-component. GDM compared to controls; Figure S42—Forest plot for Apolipoprotein E. GDM compared to controls; Figure S43—Forest plot for Fibrinogen alpha chain. GDM compared to controls; Figure S44—Forest plot for Alpha-1-antitripsin. GDM compared to controls; Supplementary Table S1—Summary of included studies (*n* = 24) on differential protein expressions in GDM and controls; Supplementary Table S2—Diagnostic criteria using the OGTT for GDM guidelines used in included studies; Supplementary Table S3—Study quality assessment using Newcastle–Ottawa scale for case-control studies; Supplementary Table S4—Replicability of 262 CB across 23 independent cohorts; Supplementary Table S5—Grouping of replicable CBs by sample type; Supplementary Table S6—Grouping of replicable CBs by proteomic platform; Supplementary Table S7—Grouping of replicable CBs by continent; Supplementary Table S8—DAVID functional annotation chart analysis representing pathways in KEGG terms; Supplementary Table S9—DAVID functional annotation chart analysis representing pathways in KEGG terms on 27 early pregnancy blood CBs for GDM excluding pilot studies; PRISMA check list; Study search strategy; Data extraction template.

## References

[1] Zhu Y., Zhang C. (2016). Prevalence of gestational diabetes and risk of progression to type 2 diabetes: A global perspective. Curr Diabetes Rep..

[2] Beucher G., Viaris de Lesegno B., Dreyfus M. (2010). Maternal outcome of gestational diabetes mellitus. Diabetes Metab..

[3] OuYang H., Chen B., Abdulrahman A.M., Li L., Wu N. (2021). Associations between Gestational Diabetes and Anxiety or Depression: A Systematic Review. J Diabetes Res..

[4] Simeonova-Krstevska S., Bogoev M., Bogoeva K., Zisovska E., Samardziski I., Velkoska-Nakova V., Livrinova V., Todorovska I., Sima A., Blazevska-Siljanoska V. (2018). Maternal and Neonatal Outcomes in Pregnant Women with Gestational Diabetes Mellitus Treated with Diet, Metformin or Insulin. Open Access Maced. J. Med. Sci..

[5] Damm P. (1998). Gestational diabetes mellitus and subsequent development of overt diabetes mellitus. Dan Med. Bull..

[6] Herath H., Herath R., Wickremasinghe R. (2017). Gestational diabetes mellitus and risk of type 2 diabetes 10 years after the index pregnancy in Sri Lankan women—A community based retrospective cohort study. PLoS ONE.

[7] (2019). American Diabetes Association. Classification and Diagnosis of Diabetes: Standards of Medical Care in Diabetes. Diabetes Care.

[8] Thomas C.C., Philipson L.H. (2015). Update on diabetes classification. Med. Clin. N. Am..

[9] Plows J.F., Stanley J.L., Baker P.N., Reynolds C.M., Vickers M.H. (2018). The pathophysiology of gestational diabetes mellitus. Int. J. Mol. Sci..

[10] Reece E.A., Leguizamón G., Wiznitzer A. (2009). Gestational diabetes: The need for a common ground. Lancet.

[11] Metzger B.E., Buchanan T.A., Coustan D.R., De Leiva A., Dunger D.B., Hadden D.R., Hod M., Kitzmiller J.L., Kjos S.L., Oats J.N. (2007). Summary and recommendations of the fifth international workshop-conference on gestational diabetes mellitus. Diabetes Care.

[12] Basri N.I., Mahdy Z.A., Ahmad S., Abdul Karim A.K., Shan L.P., Abdul Manaf M.R., Ismail N.A.M. (2018). The World Health Organization (WHO) versus The International Association of Diabetes and Pregnancy Study Group (IADPSG) diagnostic criteria of gestational diabetes mellitus (GDM) and their associated maternal and neonatal outcomes. Horm. Mol. Biol. Clin. Investig..

[13] Association American D. (2018). Updates to the Standards of Medical Care in Diabetes-Diabetes Care. Diabetes Care.

[14] Lamain-de Ruiter M., Kwee A., Naaktgeboren C.A., de Groot I., Evers I.M., Groenendaal F., Hering Y.R., Huisjes A.J., Kirpestein C., Monincx W.M. (2016). External validation of prognostic models to predict risk of gestational diabetes mellitus in one Dutch cohort: Prospective multicentre cohort study. BMJ.

[15] Chu S.Y., Callaghan W.M., Kim S.Y., Schmid C.H., Lau J., England L.J., Dietz P.M. (2007). Maternal obesity and risk of gestational diabetes mellitus. Diabetes Care.

[16] Jensen D.M., Molsted-Pedersen L., Beck-Nielsen H., Westergaard J.G., Ovesen P., Damm P. (2003). Screening for gestational diabetes mellitus by a model based on risk indicators: A prospective study. Am. J. Obs. Gynecol..

[17] Powe C.E. (2017). Early Pregnancy Biochemical Predictors of Gestational Diabetes Mellitus. Curr. Diab. Rep..

[18] Francis E.C., Li M., Hinkle S.N., Cao Y., Chen J., Wu J., Zhu Y., Cao H., Kemper K., Rennert L. (2020). Adipokines in early and mid-pregnancy and subsequent risk of gestational diabetes: A longitudinal study in a multiracial cohort. BMJ Open Diabetes Res. Care.

[19] Adya R., Tan B.K., Chen J., Randeva H.S. (2012). Protective actions of globular and full-length adiponectin on human endothelial cells: Novel insights into adiponectin induced angiogenesis. J. Vasc. Res..

[20] Bai B., Ban B., Liu Z., Zhang M.M., Tan B.K., Chen J. (2017). Circulating C1q complement/TNF-related protein (CTRP) 1, CTRP9, CTRP12 and CTRP13 concentrations in Type 2 diabetes mellitus: In vivo regulation by glucose. PLoS ONE.

[21] Deischinger C., Leitner K., Baumgartner-Parzer S., Bancher-Todesca D., Kautzky-Willer A., Harreiter J. (2020). CTRP-1 levels are related to insulin resistance in pregnancy and gestational diabetes mellitus. Sci. Rep..

[22] Lewandowski K.C., Stojanovic N., Bienkiewicz M., Tan B.K., Prelevic G.M., Press M., Tuck S., O′Hare P.J., Randeva H.S. (2008). Elevated concentrations of retinol-binding protein-4 (RBP-4) in gestational diabetes mellitus: Negative correlation with soluble vascular cell adhesion molecule-1 (sVCAM-1). Gynecol. Endocrinol..

[23] Hu J., Gillies C.L., Lin S., Stewart Z.A., Melford S.E., Abrams K.R., Baker P.N., Khunti K., Tan B.K. (2021). Association of maternal lipid profile and gestational diabetes mellitus: A systematic review and meta-analysis of 292 studies and 97880 women. EClinicalMedicine.

[24] Georgiou H.M., Lappas M., Georgiou G.M., Marita A., Bryant V.J., Hiscock R., Permezel M., Khalil Z., Rice G.E. (2008). Screening for biomarkers predictive of gestational diabetes mellitus. Acta Diabetol..

[25] Maged A.M., Moety G.A., Mostafa W.A., Hamed D.A. (2014). Comparative study between different biomarkers for early prediction of gestational diabetes mellitus. J. Matern. Fetal Neonatal. Med..

[26] Nanda S., Savvidou M., Syngelaki A., Akolekar R., Nicolaides K.H. (2011). Prediction of gestational diabetes mellitus by maternal factors and biomarkers at 11 to 13 weeks. Prenat. Diagn..

[27] Caglar G.S., Ozdemir E.D., Cengiz S.D., Demirtas S. (2012). Sex-hormone-binding globulin early in pregnancy for the prediction of severe gestational diabetes mellitus and related complications. J. Obstet. Gynaecol. Res..

[28] Iliodromiti S., Sassarini J., Kelsey T.W., Lindsay R.S., Sattar N., Nelson S.M. (2016). Accuracy of circulating adiponectin for predicting gestational diabetes: A systematic review and meta-analysis. Diabetologia.

[29] Qiu C., Sorensen T.K., Luthy D.A., Williams M.A. (2004). A prospective study of maternal serum C-reactive protein (CRP) concentrations and risk of gestational diabetes mellitus. Paediatr. Perinat. Epidemiol..

[30] Savvidou M., Nelson S.M., Makgoba M., Messow C.M., Sattar N., Nicolaides K. (2010). First-trimester prediction of gestational diabetes mellitus: Examining the potential of combining maternal characteristics and laboratory measures. Diabetes.

[31] Theriault S., Giguere Y., Masse J., Girouard J., Forest J.C. (2016). Early prediction of gestational diabetes: A practical model combining clinical and biochemical markers. Clin. Chem. Lab. Med..

[32] Tambor V., Fucikova A., Lenco J., Kacerovsky M., Rehacek V., Stulik J., Pudil R. (2010). Application of proteomics in biomarker discovery: A primer for the clinician. Physiol. Res..

[33] Li K.W., Gonzalez-Lozano M.A., Koopmans F., Smit A.B. (2020). Recent Developments in Data Independent Acquisition (DIA) Mass Spectrometry: Application of Quantitative Analysis of the Brain Proteome. Front. Mol. Neurosci..

[34] Huang J., Chen X., Fu X., Li Z., Huang Y., Liang C. (2021). Advances in Aptamer-Based Biomarker Discovery. Front Cell Dev. Biol..

[35] Liberati A., Altman D.G., Tetzlaff J., Mulrow C., Gøtzsche P.C., Ioannidis J.P.A., Clarke M., Devereaux P.J., Kleijnen J., Moher D. (2009). The prisma statement for reporting systematic reviews and meta-analyses of studies that evaluate health care interventions: Explanation and elaboration. PLoS Med..

[36] Higgins J., Thomas J., Chandler J., Cumpston M., Li T., Page M., Welch V. (2021). Cochrane Handbook for Systematic Reviews of Interventions Version 6.2 (Updated February 2021).

[37] Higgins J.P., Thompson S.G. (2002). Quantifying heterogeneity in a meta-analysis. Stat. Med..

[38] Huang da W., Sherman B.T., Lempicki R.A. (2009). Systematic and integrative analysis of large gene lists using DAVID bioinformatics resources. Nat. Protoc..

[39] Shen L.J., Chen F.Y., Cao L.F., Zhang Y., Zhong H. (2012). Functional Enrichment Analysis by David in Transgenic MYCN Zebrafish Model. Blood.

[40] Ravnsborg T., Andersen L.L.T., Trabjerg N.D., Rasmussen L.M., Jensen D.M., Overgaard M. (2016). First-trimester multimarker prediction of gestational diabetes mellitus using targeted mass spectrometry. Diabetologia.

[41] Ravnsborg T., Svaneklink S., Andersen L.L.T., Larsen M.R., Jensen D.M., Overgaard M. (2019). First-trimester proteomic profiling identifies novel predictors of gestational diabetes mellitus. PLoS ONE.

[42] Boisvert M.R., Koski K.G., Skinner C.D. (2010). Increased Oxidative Modifications of Amniotic Fluid Albumin in Pregnancies Associated with Gestational Diabetes Mellitus. Anal. Chem..

[43] Kim S.M., Park J.S., Norwitz E.R., Lee S.M., Kim B.J., Park C.-W., Jun J.K., Kim C.-W., Syn H.C. (2012). Identification of Proteomic Biomarkers in Maternal Plasma in the Early Second Trimester That Predict the Subsequent Development of Gestational Diabetes. Reprod. Sci..

[44] Fruscalzo A., Londero A.P., Driul L., Henze A., Tonutti L., Ceraudo M., Zanotti G., Berni R., Schweigert F.J., Raila J. (2015). First trimester concentrations of the TTR-RBP4-retinol complex components as early markers of insulin-treated gestational diabetes mellitus. Clin. Chem. Lab. Med..

[45] Boyle K.E., Hwang H., Janssen R.C., DeVente J.M., Barbour L.A., Hernandez T.L., Mandarino L.J., Lappas M., Friedman J.E. (2014). Gestational Diabetes Is Characterized by Reduced Mitochondrial Protein Expression and Altered Calcium Signaling Proteins in Skeletal Muscle. PLoS ONE.

[46] Ai T., Chen F., Zhou S., Zhang J., Zheng H., Zhou Y., Hu W., Liu X., Li L., Lin J. (2015). Magnetic Bead-Based Serum Peptidome Profiling in Patients with Gestational Diabetes Mellitus. Biomed. Res. Int..

[47] Liu B., Xu Y., Voss C., Qiu F.H., Zhao M.Z., Liu Y.D., Nie J., Wang Z.L. (2012). Altered Protein Expression in Gestational Diabetes Mellitus Placentas Provides Insight into Insulin Resistance and Coagulation/Fibrinolysis Pathways. PLoS ONE.

[48] Hajduk J., Klupczynska A., Derezinski P., Matysiak J., Kokot P., Nowak D.M., Gajecka M., Nowak-Markwitz E., Kokot Z.J. (2015). A Combined Metabolomic and Proteomic Analysis of Gestational Diabetes Mellitus. Int. J. Mol. Sci..

[49] Zhao C., Wang F., Wang P., Ding H., Huang X., Shi Z. (2015). Early second-trimester plasma protein profiling using multiplexed isobaric tandem mass tag (TMT) labeling predicts gestational diabetes mellitus. Acta Diabetol..

[50] Liu F., Zhao C., Liu L., Ding H., Huo R., Shi Z. (2016). Peptidome profiling of umbilical cord plasma associated with gestational diabetes-induced fetal macrosomia. J. Proteom..

[51] Zhao D., Shen L., Wei Y., Xie J., Chen S., Liang Y., Chen Y., Wu H. (2017). Identification of candidate biomarkers for the prediction of gestational diabetes mellitus in the early stages of pregnancy using iTRAQ quantitative proteomics. Proteom. Clin. Appl..

[52] Miao Z., Wang J., Wang F., Liu L., Ding H., Shi Z. (2016). Comparative proteomics of umbilical vein blood plasma from normal and gestational diabetes mellitus patients reveals differentially expressed proteins associated with childhood obesity. Proteom. Clin. Appl..

[53] Shen L., Zhao D., Chen Y., Zhang K., Chen X., Lin J., Li C., Iqbal J., Zhao Y., Liang Y. (2019). Comparative Proteomics Analysis of Serum Proteins in Gestational Diabetes during Early and Middle Stages of Pregnancy. Proteom. Clin. Appl..

[54] Liu X., Sun J., Wen X., Duan J., Xue D., Pan Y., Sun J., Zhang W., Cheng X., Wang C. (2020). Proteome profiling of gestational diabetes mellitus at 16-18 weeks revealed by LC-MS/MS. J. Clin. Lab. Anal.

[55] Li J., Lu L., Xie X., Dai X., Zheng S., Chen L. (2021). Proteomics Analysis of Serum Proteins in Gestational Diabetes. Evid. Based Complement. Altern. Med..

[56] Ma Y., Gao J., Yin J., Gu L., Liu X., Chen S., Huang Q., Lu H., Yang Y., Zhou H. (2016). Identification of a Novel Function of Adipocyte Plasma Membrane-Associated Protein (APMAP) in Gestational Diabetes Mellitus by Proteomic Analysis of Omental Adipose Tissue. J. Proteome Res..

[57] Guo Y., Han Z., Guo L., Liu Y., Li G., Li H., Zhang J., Bai L., Wu H., Chen B. (2018). Identification of urinary biomarkers for the prediction of gestational diabetes mellitus in early second trimester of young gravidae based on iTRAQ quantitative proteomics. Endocr. J..

[58] Liao Y., Xu G.-F., Jiang Y., Zhu H., Sun L.-J., Peng R., Luo Q. (2018). Comparative proteomic analysis of maternal peripheral plasma and umbilical venous plasma from normal and gestational diabetes mellitus pregnancies. Medicine.

[59] Jayabalan N., Lai A., Nair S., Guanzon D., Scholz-Romero K., Palma C., McIntyre H.D., Lappas M., Salomon C. (2019). Quantitative Proteomics by SWATH-MS Suggest an Association Between Circulating Exosomes and Maternal Metabolic Changes in Gestational Diabetes Mellitus. Proteomics.

[60] Ramachandrarao S.P., Hamlin A.A., Awdishu L., Overcash R., Zhou M., Proudfoot J., Ishaya M., Aghania E., Madrigal A., Kokoy-Mondragon C. (2016). Proteomic analyses of Urine Exosomes reveal New Biomarkers of Diabetes in Pregnancy. Madr. J. Diabetes.

[61] Ilyas S., Roohi N., Ashraf S., Alyas S. (2020). Upregulated Retinol Binding Protein and Transthyretin as Predictive Biomarkers of Gestational Diabetes Mellitus. Curr. Proteom..

[62] Kopylov A.T., Kaysheva A.L., Papysheva O., Gribova I., Kotaysch G., Kharitonova L., Mayatskaya T., Krasheninnikova A., Morozov S.G. (2020). Association of Proteins Modulating Immune Response and Insulin Clearance During Gestation with Antenatal Complications in Patients with Gestational or Type 2 Diabetes Mellitus. Cells.

[63] Adkins J.N., Varnum S.M., Auberry K.J., Moore R.J., Angell N.H., Smith R.D., Springer D.L., Pounds J.G. (2002). Toward a human blood serum proteome: Analysis by multidimensional separation coupled with mass spectrometry. Mol. Cell Proteom..

[64] Steel L.F., Trotter M.G., Nakajima P.B., Mattu T.S., Gonye G., Block T. (2003). Efficient and specific removal of albumin from human serum samples. Mol. Cell Proteom..

[65] Chen B.K., Overgaard M.T., Bale L.K., Resch Z.T., Christiansen M., Oxvig C., Conover C.A. (2002). Molecular regulation of the IGF-binding protein-4 protease system in human fibroblasts: Identification of a novel inducible inhibitor. Endocrinology.

[66] Andersen C.B.F., Stodkilde K., Saederup K.L., Kuhlee A., Raunser S., Graversen J.H., Moestrup S.K. (2017). Haptoglobin. Antioxid Redox Signal..

[67] Anderson N.L., Anderson N.G. (2002). The human plasma proteome: History, character, and diagnostic prospects. Mol. Cell Proteom..

[68] Geyer P.E., Holdt L.M., Teupser D., Mann M. (2017). Revisiting biomarker discovery by plasma proteomics. Mol. Syst. Biol..

[69] Dayon L., Affolter M. (2020). Progress and pitfalls of using isobaric mass tags for proteome profiling. Expert Rev. Proteom..

[70] Moulder R., Bhosale S.D., Goodlett D.R., Lahesmaa R. (2018). Analysis of the plasma proteome using iTRAQ and TMT-based Isobaric labeling. Mass Spectrom Rev..

[71] Gangabadage C.S., Zdunek J., Tessari M., Nilsson S., Olivecrona G., Wijmenga S.S. (2008). Structure and dynamics of human apolipoprotein CIII. J. Biol Chem..

[72] Campos H., Perlov D., Khoo C., Sacks F.M. (2001). Distinct patterns of lipoproteins with apoB defined by presence of apoE or apoC-III in hypercholesterolemia and hypertriglyceridemia. J. Lipid Res..

[73] Jong M.C., Hofker M.H., Havekes L.M. (1999). Role of ApoCs in lipoprotein metabolism: Functional differences between ApoC1, ApoC2, and ApoC. Arter. Thromb. Vasc. Biol..

[74] Huang X.S., Zhao S.P., Hu M., Bai L., Zhang Q., Zhao W. (2010). Decreased apolipoprotein A5 is implicated in insulin resistance-related hypertriglyceridemia in obesity. Atherosclerosis.

[75] Ben Khedher M.R., Haddad M., Laurin D., Ramassamy C. (2021). Apolipoprotein E4-driven effects on inflammatory and neurotrophic factors in peripheral extracellular vesicles from cognitively impaired, no dementia participants who converted to Alzheimer’s disease. Alzheimers Dement..

[76] Marais A.D. (2019). Apolipoprotein E in lipoprotein metabolism, health and cardiovascular disease. Pathology.

[77] Li M., Hou X., Zhang R., Zheng X., Dang W. (2021). Apolipoprotein E deficiency correlates to oxidative stress in gestational diabetes mellitus. Int. J. Gynaecol. Obstet..

[78] Othman M., Santamaria Ortiz A., Cerda M., Erez O., Minford A., Obeng-Tuudah D., Blondon M., Bistervels I., Middeldorp S., Abdul-Kadir R. (2019). Thrombosis and hemostasis health in pregnancy: Registries from the International Society on Thrombosis and Haemostasis. Res. Pract. Thromb. Haemost..

[79] Renne T., Schmaier A.H., Nickel K.F., Blomback M., Maas C. (2012). In vivo roles of factor XII. Blood.

[80] Ozbasli E., Takmaz O., Karabuk E., Gungor M. (2020). Comparison of factor XII levels in gestational diabetes, fetal macrosomia, and healthy pregnancies. BMC Pregnancy Childbirth.

[81] Zhang J., Wright W., Bernlohr D.A., Cushman S.W., Chen X. (2007). Alterations of the classic pathway of complement in adipose tissue of obesity and insulin resistance. Am. J. Physiol. Endocrinol. Metab..

[82] Litvinov R.I., Weisel J.W. (2017). Fibrin mechanical properties and their structural origins. Matrix Biol..

[83] Wang X.R., Wang W.J., Yu X., Hua X., Ouyang F., Luo Z.C. (2019). Insulin-Like Growth Factor Axis Biomarkers and Gestational Diabetes Mellitus: A Systematic Review and Meta-Analysis. Front. Endocrinol..

[84] Marcelino J., Carpten J.D., Suwairi W.M., Gutierrez O.M., Schwartz S., Robbins C., Sood R., Makalowska I., Baxevanis A., Johnstone B. (1999). CACP, encoding a secreted proteoglycan, is mutated in camptodactyly-arthropathy-coxa vara-pericarditis syndrome. Nat. Genet..

[85] Chen C.P., Chang S.C., Vivian Yang W.C. (2007). High Glucose Alters Proteoglycan Expression and the Glycosaminoglycan Composition in Placentas of Women with Gestational Diabetes Mellitus and in Cultured Trophoblasts. Placenta.

[86] Bottazzi B., Doni A., Garlanda C., Mantovani A. (2010). An Integrated View of Humoral Innate Immunity: Pentraxins as a Paradigm. Annu. Rev. Immunol..

[87] Yuan J., Shen Y., Long Y., Yu F., Xie Y., Lin X., Zhao Y., Tian M., Dong R., Zha Y. (2016). Serum amyloid P component regulated the development of diabetic nephropathy via down regulation of CCL-1. Int. J. Clin. Exp. Pathol..

[88] Sorenson R.C., Bisgaier C.L., Aviram M., Hsu C., Billecke S., La Du B.N. (1999). Human serum paraoxonase/arylesterase’s retained hydrophobic N-terminal leader sequence associates with HDLs by binding phospholipids: Apolipoprotein AI stabilizes activity. Arterioscler. Thromb. Vasc. Biol..

[89] Kishore U., Reid K.B.M. (2000). C1q: Structure, function, and receptors. Immunopharmacology.

[90] Ghosh P., Sahoo R., Vaidya A., Chorev M., Halperin J.A. (2015). Role of complement and complement regulatory proteins in the complications of diabetes. Endocr. Rev..

[91] Harrison R.A., Lachmann P.J. (1980). The physiological breakdown of the third component of human complement. Mol. Immunol..

[92] Zhu C., Yang H., Geng Q., Ma Q., Long Y., Zhou C., Chen M. (2015). Association of oxidative stress biomarkers with gestational diabetes mellitus in pregnant women: A case-control study. PLoS ONE.

[93] Ramanjaneya M., Butler A.E., Alkasem M., Bashir M., Jerobin J., Godwin A., Moin A.S.M., Ahmed L., Elrayess M.A., Hunt S.C. (2021). Association of Complement-Related Proteins in Subjects With and Without Second Trimester Gestational Diabetes. Front. Endocrinol..

[94] Kulak K., Westermark G.T., Papac-Milicevic N., Renström E., Blom A.M., King B.C. (2017). The human serum protein C4b-binding protein inhibits pancreatic IAPP-induced inflammasome activation. Diabetologia.

[95] Koch G.L., Macer D.R., Wooding F.B. (1988). Endoplasmin is a reticuloplasmin. J. Cell Sci..

[96] Scheuner D., Vander Mierde D., Song B., Flamez D., Creemers J.W., Tsukamoto K., Ribick M., Schuit F.C., Kaufman R.J. (2005). Control of mRNA translation preserves endoplasmic reticulum function in beta cells and maintains glucose homeostasis. Nat Med..

[97] Buchanan T.A., Xiang A.H., Page K.A. (2012). Gestational diabetes mellitus: Risks and management during and after pregnancy. Nat. Rev. Endocrinol..

[98] Tomas A., Yermen B., Min L., Pessin J.E., Halban P.A. (2006). Regulation of pancreatic β-cell insulin secretion by actin cytoskeleton remodelling: Role of gelsolin and cooperation with the MAPK signalling pathway. J. Cell Sci..

[99] Kalwat M.A., Wiseman D.A., Luo W., Wang Z., Thurmond D.C. (2012). Gelsolin associates with the N terminus of syntaxin 4 to regulate insulin granule exocytosis. Mol. Endocrinol..

[100] Khatri N., Sagar A., Peddada N., Choudhary V., Chopra B.S., Garg V., Garg R.A. (2014). Plasma Gelsolin Levels Decrease in Diabetic State and Increase upon Treatment with F-Actin Depolymerizing Versions of Gelsolin. J. Diabetes Res..

[101] Schroeder H.W., Cavacini L. (2010). Structure and function of immunoglobulins. J. Allergy Clin. Immunol..

[102] Hu Z., Tian Y., Li J., Hu M., Hou J., Zhang M. (2021). Coagulation Index and Pregnancy Outcome in Gestational Diabetes Mellitus. Clin Lab..

[103] Turner M.E., White C.A., Taylor S.M., Neville K., Rees-Milton K., Hopman W.M., Adams M.A., Anastassiades T., Holden R.M. (2021). Secreted Phosphoprotein 24 is a Biomarker of Mineral Metabolism. Calcif Tissue Int..

[104] Chen J., Tan B., Karteris E., Zervou S., Digby J., Hillhouse E.W., Vatish M., Randeva H.S. (2006). Secretion of adiponectin by human placenta: Differential modulation of adiponectin and its receptors by cytokines. Diabetologia.

[105] Hakimi A., Auluck J., Jones G.D., Ng L.L., Jones D.J. (2014). Assessment of reproducibility in depletion and enrichment workflows for plasma proteomics using label-free quantitative data-independent LC-MS. Proteomics.

[106] Tu C., Rudnick P.A., Martinez M.Y., Cheek K.L., Stein S.E., Slebos R.J.C., Liebler D.C. (2010). Depletion of Abundant Plasma Proteins and Limitations of Plasma Proteomics. J. Proteome Res..

[107] Hortin G.L., Sviridov D. (2010). The dynamic range problem in the analysis of the plasma proteome. J. Proteom..

