# Supplementary material for: Proteomics Studies in Gestational Diabetes Mellitus: A Systematic Review and Meta-Analysis
Source: J Clin Med. 2022 May 12;11(10):2737. doi: 10.3390/jcm11102737 (PMC9143836; doi:10.3390/jcm11102737)
Supplement: Supplementary file 1 [file jcm-11-02737-s001.zip › jcm-1695841-SI/Data extraction template.pdf]

Data extraction template

No.	Parameter	Data
1	Author (Ref)	
2	Year	
3	DOI	
4	OGTT Test	
5	Diag Criteria	
6	Glucose Dose	
7	Country of study	
8	Continent	
9	Ethnicity	
10	Gestational age at sample collection for Proteomics Analysis	
11	Trimester (sample collection for Proteomics Analysis)	
12	Gestation age for GDM diagnosis	
13	Trimester (GDM diagnosis)	
14	Study Type	
15	Comparison	
16	Control (n)	
17	Con_Age_mean (years)	
18	Con_Age_SD	
19	Con_BMI_mean (kg/m^2)	
20	Con_BMI_SD	
21	Con_SBP_mean (Systolic Blood Pressure)	
22	Con_SBP_SD	
23	Con_DBP_mean (Diastolic Blood Pressure)	
24	Con_DBP_SD	
25	GDM (n)	
26	GDM_Age_mean (years)	
27	GDM_Age_SD	
28	GDM_BMI_mean	
29	GDM_BMI_SD	

30	GDM_SBP_mean (Systolic Blood Pressure)	
31	GDM_SBP_SD	
32	GDM_DBP_mean (Diastolic Blood Pressure)	
33	GDM_DBP_SD	
34	Fasting	
35	Proteinuria	
36	Exclusion	
37	Treatments	
38	Sample pooling	
39	Replications	
40	Special Sample processing (Preparation)	
41	Profiling (proteins or peptides)	
42	Platform	
43	Ref data	
44	Quality control for proteomic result	
45	Statistical method for differential expression	
46	Cut off	
47	Sample type	
48	Candidate Biomarker	
49	Protein ID	
50	Uniprot	
51	Regulation	
52	Fold change	
53	AUC	
54	p-value	
55	Proteomic platform	
56	Protein/Peptide number (total)	
57	Protein/Peptide number (differential expressed)	
58	Year of sample collection	
59	Year of sample analysis	

60	Verification and Validation (Technique)	
61	Diagnostic/validation panel (Proteins/peptides)	
62	Newcastle-Ottawa Score	
63	Note*	
