# Supplementary material for: Proteomics Studies in Gestational Diabetes Mellitus: A Systematic Review and Meta-Analysis
Source: J Clin Med. 2022 May 12;11(10):2737. doi: 10.3390/jcm11102737 (PMC9143836; doi:10.3390/jcm11102737)
Supplement: Supplementary file 1 [file jcm-11-02737-s001.zip › jcm-1695841-SI/Study search strategy.pdf]

Study search strategy

We conducted a systematic search of articles of interest in MEDLINE, EMBASE, Web of Science and Scopus

Search strategy
Search terms related to studies were combined in the following strategy
MeSH terms: “"Proteomics" and “Gestational diabetes mellitus”

Keywords for Gestational diabetes mellitus: “Gestational Diabetes” or “Diabetes Mellitus Gestational” or “Pregnancy-Induced Diabetes” or "GDM"

Keywords for Proteomics analysis: "Proteomics" or "Proteomic" and "Mass Spectrometry"

We performed the search using above strategy in the database "MEDLINE", "EMBASE", "Web of Science" and "Scopus" on May 2020 and on January 2022
and performed the search in “CINHAL” and the Cochrane Library on June 2020 and on February 2021.

In the search syntax we did not added any restriction on publication dates, while we only selected articles written in English.

In addition, hand searching the reference list for eligible studies and direct contact with authors, when necessary.
