# Supplementary material for: Proteomics Studies in Gestational Diabetes Mellitus: A Systematic Review and Meta-Analysis
Source: J Clin Med. 2022 May 12;11(10):2737. doi: 10.3390/jcm11102737 (PMC9143836; doi:10.3390/jcm11102737)
Supplement: Supplementary file 1 [file jcm-11-02737-s001.zip › jcm-1695841-SI/Supplementary Figure 41.pdf]

Figure S41a. Forest plot for Serum amyloid P-component. GDM compared to controls.

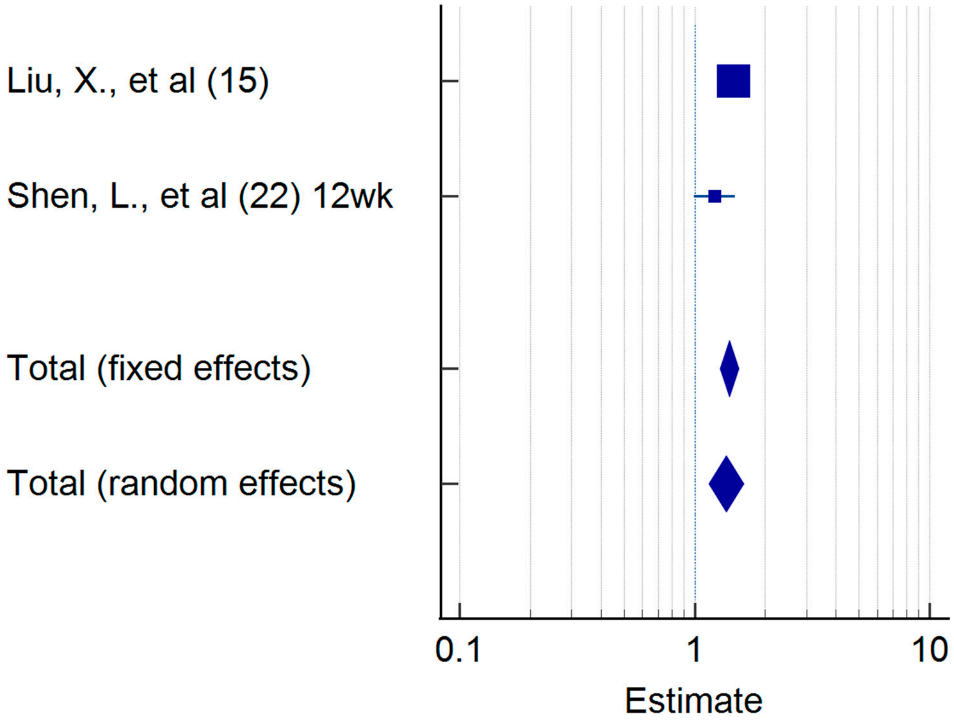

Protein	Study	RoM	95%CI	P	Weight (%)
Serum amyloid P-component	Liu, X., et al (15)	1.460	1.318 to 1.618		61.72
	Shen, L., et al (22) 12wk	1.220	1.000 to 1.488		38.28
	Total (random effects)	1.363	1.149 to 1.617	<0.001	100.00
Heterogeneity	$I^2 = 59.55$ %, P = 0.1159				

Figure S41. Forest plot for Secreted phosphoprotein 24. GDM compared to controls.

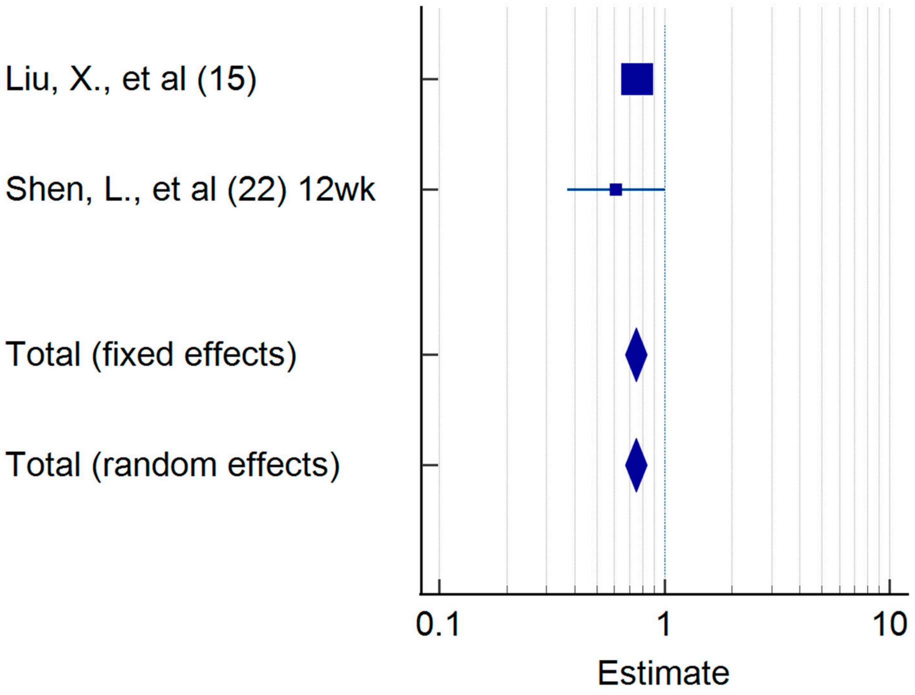

Protein	Study	RoM	95%CI	P	Weight (%)
Secreted phosphoprotein 24	Liu, X., et al (15)	0.757	0.676 to 0.849		94.96
	Shen, L., et al (22) 12wk	0.610	0.372 to 1.000		5.04
	Total (random effects)	0.749	0.670 to 0.837	<0.001	100.00
Heterogeneity	$I^2 = 0.00$ %, P = 0.4032				

Figure S41c. Forest plot for Apolipoprotein A-V. GDM compared to controls.

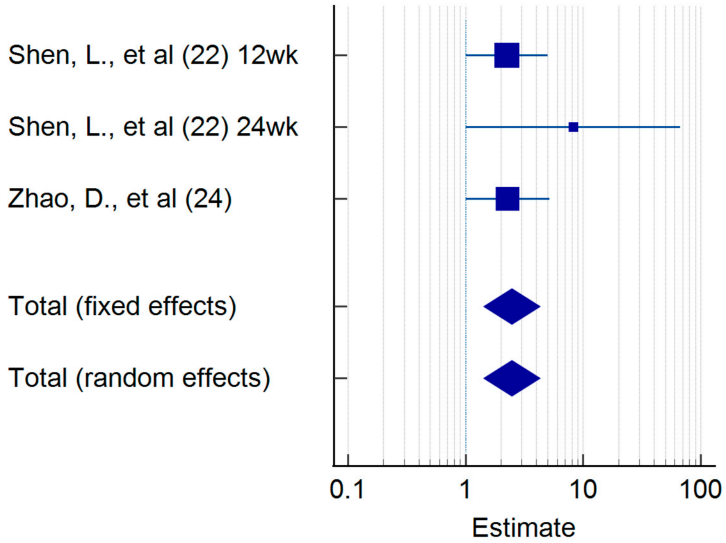

Protein	Study	RoM	95%CI	P	Weight (%)
Apolipoprotein A-V	Shen, L., et al (22) 12wk	2.240	1.000 to 5.018		47.52
	Shen, L., et al (22) 24wk	8.200	1.000 to 67.243		6.98
	Zhao, D., et al (24)	2.280	1.000 to 5.198		45.50
	Total (random effects)	2.472	1.418 to 4.311	0.001	100.00
Heterogeneity	$I^2 = 0.00$ %, P = 0.5112				
