# Supplementary material for: Proteomics Studies in Gestational Diabetes Mellitus: A Systematic Review and Meta-Analysis
Source: J Clin Med. 2022 May 12;11(10):2737. doi: 10.3390/jcm11102737 (PMC9143836; doi:10.3390/jcm11102737)
Supplement: Supplementary file 1 [file jcm-11-02737-s001.zip › jcm-1695841-SI/Supplementary Figure 42.pdf]

Figure S42a. Forest plot for Apolipoprotein E. GDM compared to controls.

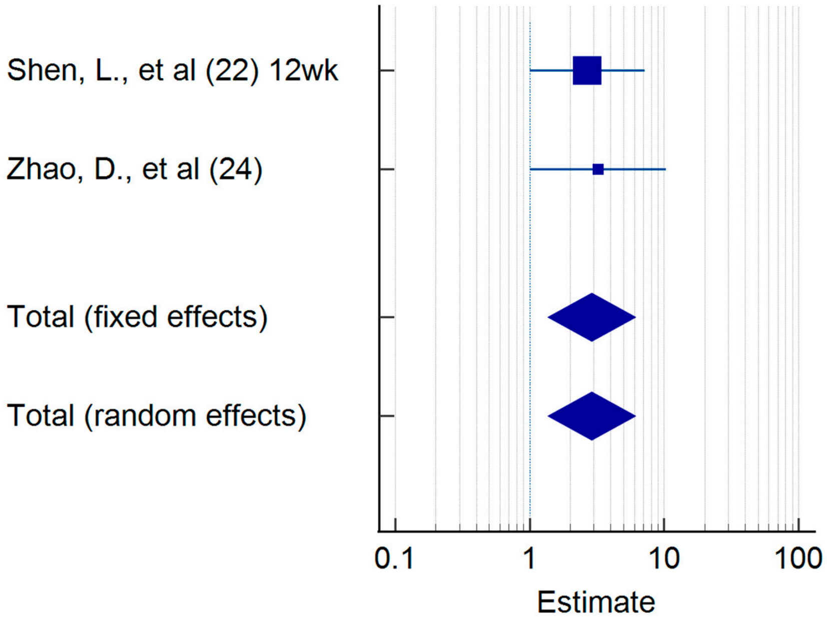

Protein	Study	RoM	95%CI	P	Weight (%)
Apolipoprotein E	Shen, L., et al (22) 12wk	2.680	1.000 to 7.183		58.33
	Zhao, D., et al (24)	3.210	1.000 to 10.304		41.67
	Total (random effects)	2.889	1.361 to 6.134	0.006	100.00
Heterogeneity	$I^2 = 0.00$ %, $P = 0.8168$				

Figure S42b. Forest plot for Coagulation factor IX. GDM compared to controls.

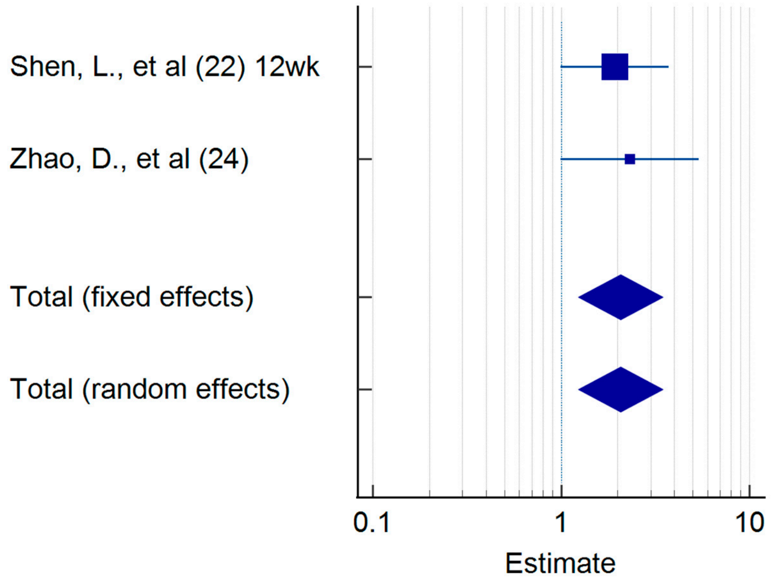

Protein	Study	RoM	95%CI	P	Weight (%)
Coagulation factor IX	Shen, L., et al (22) 12wk	1.930	1.000 to 3.725		62.09
	Zhao, D., et al (24)	2.320	1.000 to 5.382		37.91
	Total (random effects)	2.069	1.233 to 3.474	0.006	100.00
Heterogeneity	$I^2 = 0.00$ %, P = 0.7355				

Figure S42c. Forest plot for Coagulation factor X. GDM compared to controls.

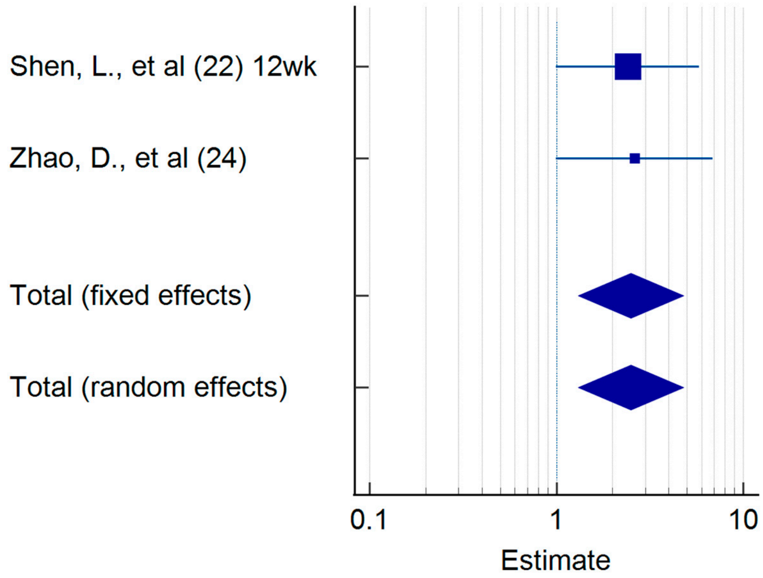

Protein	Study	RoM	95%CI	P	Weight (%)
Coagulation factor X	Shen, L., et al (22) 12wk	2.410	1.000 to 5.808		54.52
	Zhao, D., et al (24)	2.620	1.000 to 6.865		45.48
	Total (random effects)	2.503	1.307 to 4.793	0.006	100.00
Heterogeneity	$I^2 = 0.00$, $P = 0.9001$				

Figure S42d. Forest plot for Coagulation factor XII. GDM compared to controls.

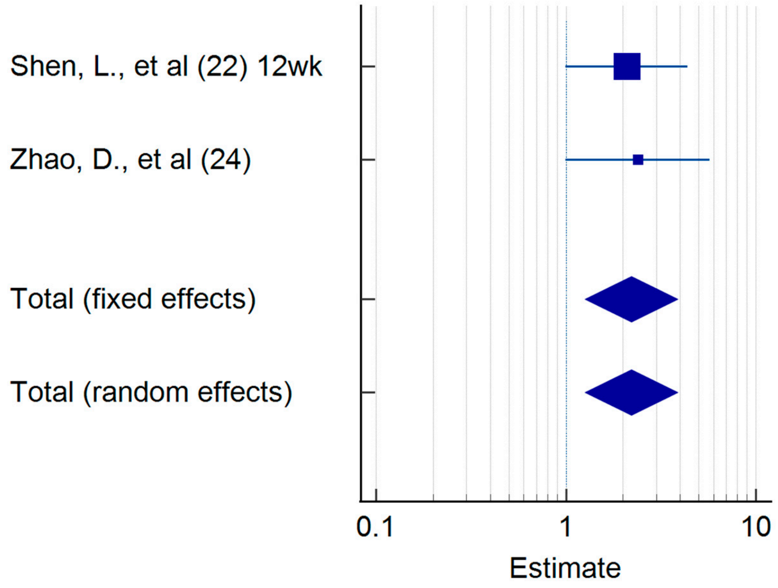

Protein	Study	RoM	95%CI	P	Weight (%)
Coagulation factor XII	Shen, L., et al (22) 12wk	2.090	1.000 to 4.368		58.28
	Zhao, D., et al (24)	2.390	1.000 to 5.712		41.72
	Total (random effects)	2.210	1.259 to 3.880	0.006	100.00
Heterogeneity	$I^2 = 0.00$ %, P = 0.8178				

Figure S42e. Forest plot for Complement C1s subcomponent. GDM compared to controls.

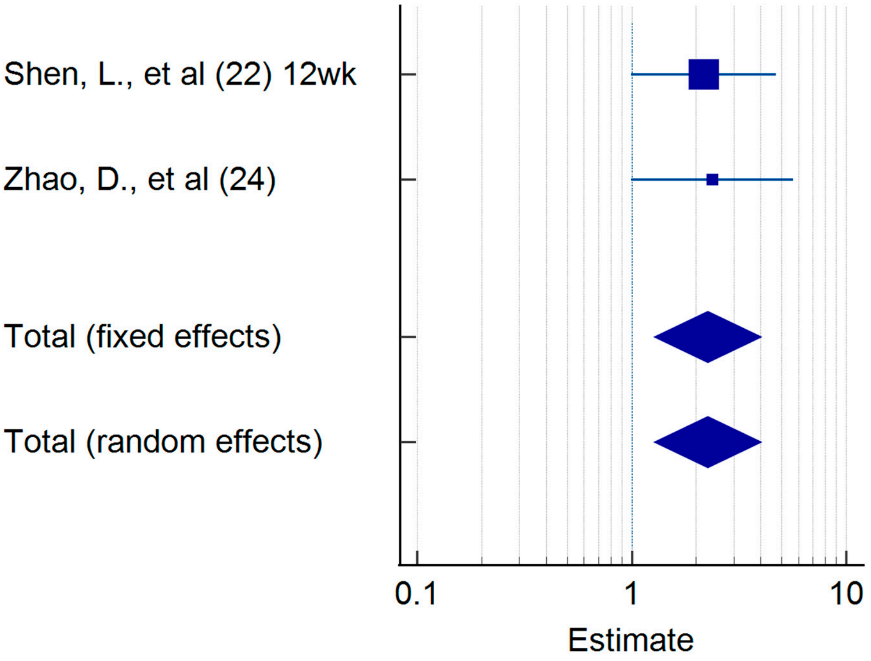

Protein	Study	RoM	95%CI	P	Weight (%)
Complement C1s subcomponent	Shen, L., et al (22) 12wk	2.170	1.000 to 4.709		55.61
	Zhao, D., et al (24)	2.380	1.000 to 5.664		44.39
	Total (random effects)	2.261	1.269 to 4.029	0.006	100.00
Heterogeneity	I^2 = 0.00 %, P = 0.8763				

Figure S42f. Forest plot for Insulin-like growth factor-binding protein 5. GDM compared to controls.

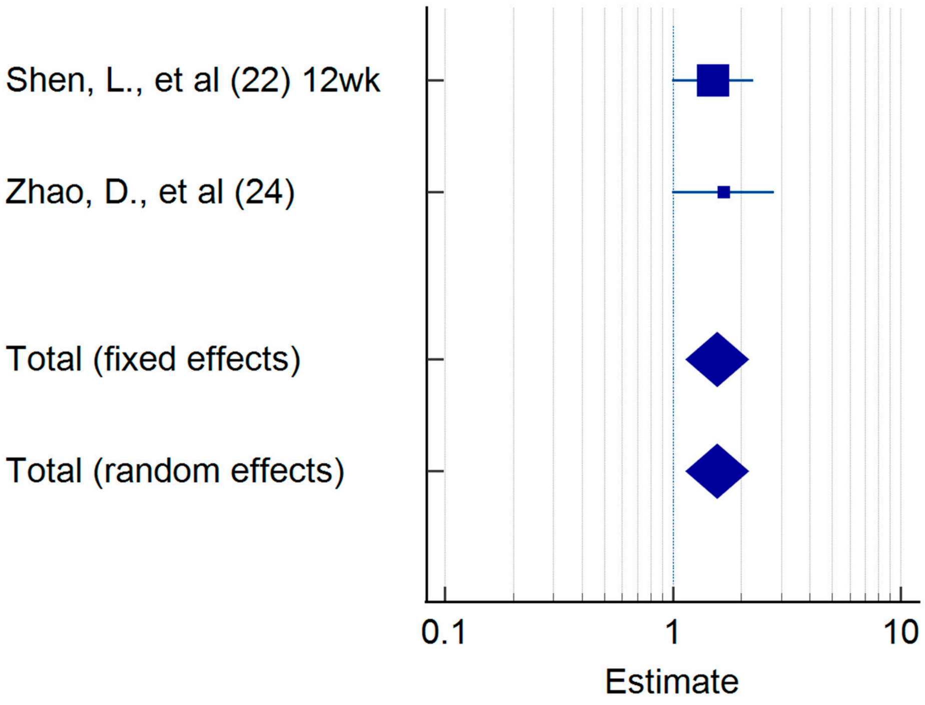

Protein	Study	RoM	95%CI	P	Weight (%)
Insulin-like growth factor-binding protein 5	Shen, L., et al (22) 12wk	1.500	1.000 to 2.250		61.53
	Zhao, D., et al (24)	1.670	1.000 to 2.789		38.47
	Total (random effects)	1.563	1.137 to 2.149	0.006	100.00
Heterogeneity	I^2 = 0.00 %, P = 0.7476				

Figure S42g. Forest plot for Serum amyloid P-component. GDM compared to controls.

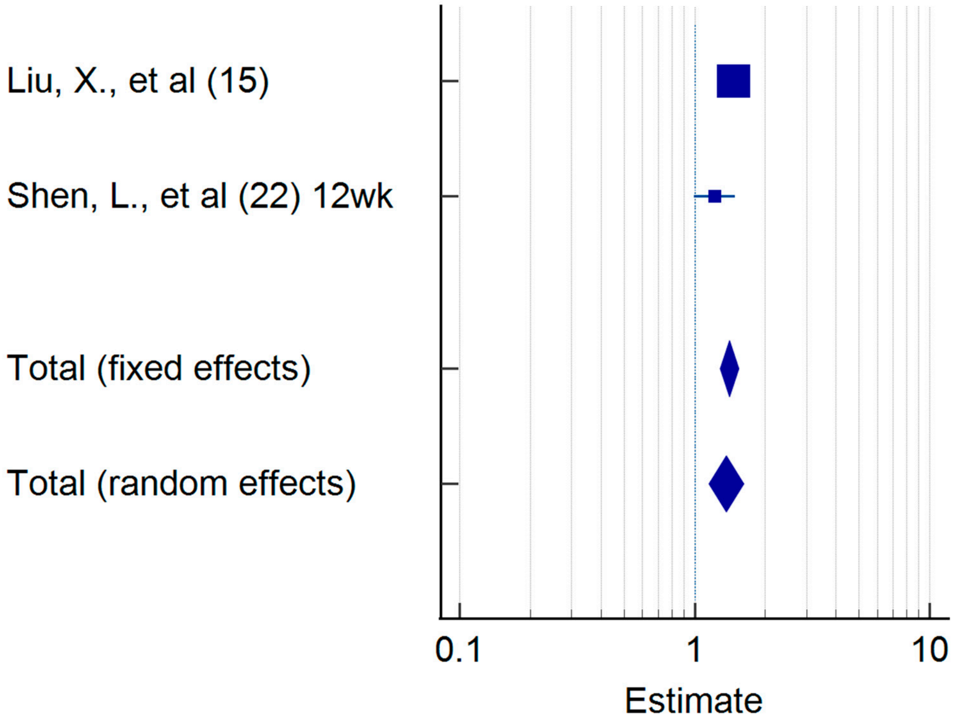

Protein	Study	RoM	95%CI	P	Weight (%)
Serum amyloid P-component	Liu, X., et al (15)	1.460	1.318 to 1.618		61.72
	Shen, L., et al (22) 12wk	1.220	1.000 to 1.488		38.28
	Total (random effects)	1.363	1.149 to 1.617	<0.001	100.00
Heterogeneity	I^2 = 59.55 %, P = 0.1159				

Figure S42h. Forest plot for Serum paraoxonase/arylesterase 1. GDM compared to controls.

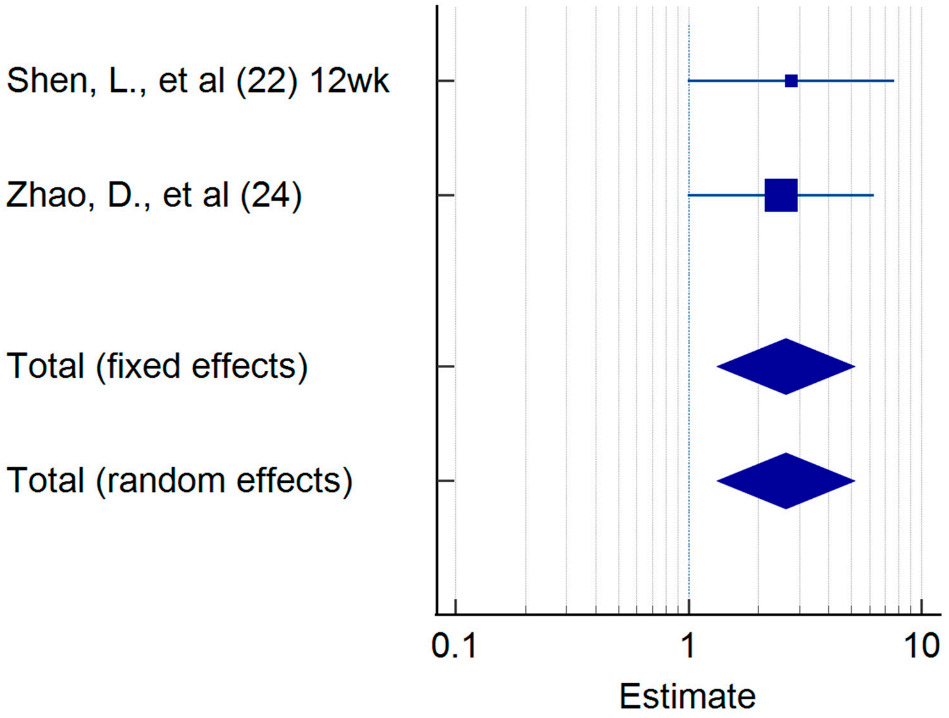

Protein	Study	RoM	95%CI	P	Weight (%)
Serum paraoxonase/arylesterase 1	Shen, L., et al (22) 12wk	2.770	1.000 to 7.673		44.72
	Zhao, D., et al (24)	2.500	1.000 to 6.250		55.28
	Total (random effects)	2.617	1.324 to 5.173	0.006	100.00
Heterogeneity	$I^2 = 0.00\%$, P = 0.8834				

Figure S42i. Forest plot for C4b-binding protein alpha chain. GDM compared to controls.

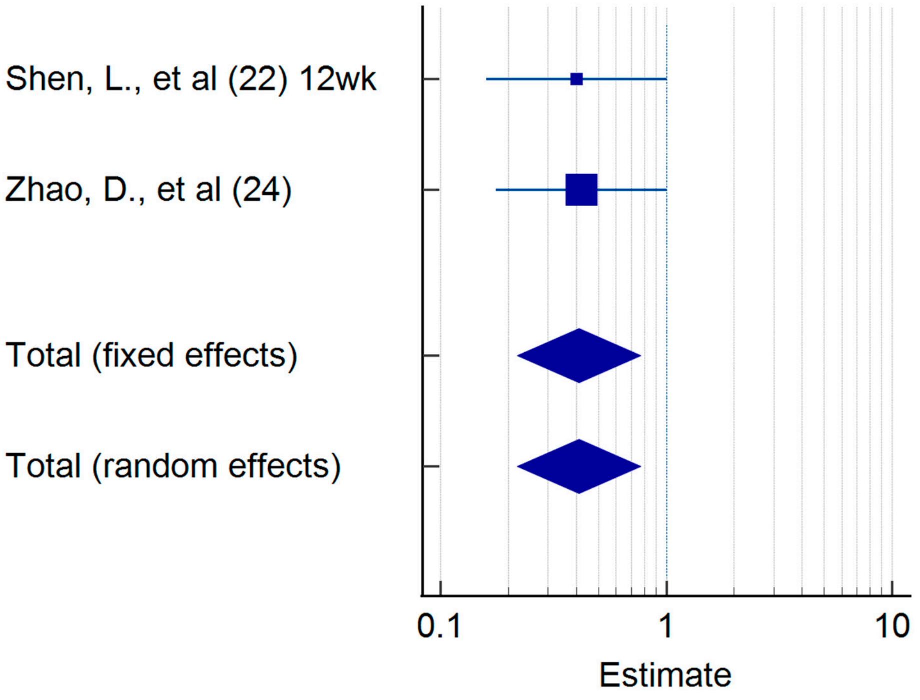

Protein	Study	RoM	95%CI	P	Weight (%)
C4b-binding protein alpha chain	Shen, L., et al (22) 12wk	0.400	0.160 to 1.000		47.27
	Zhao, D., et al (24)	0.420	0.176 to 1.000		52.73
	Total (random effects)	0.410	0.219 to 0.771	0.006	100.00
Heterogeneity	$I^2 = 0.00$ %, P = 0.9396				

Figure S42j. Forest plot for Coagulation factor V. GDM compared to controls.

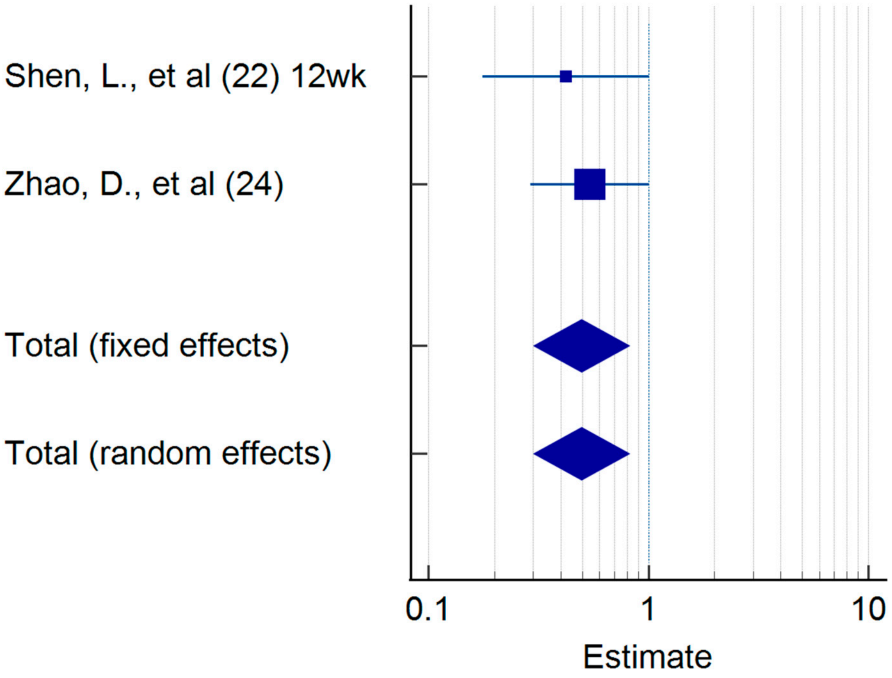

Protein	Study	RoM	95%CI	P	Weight (%)
Coagulation factor V	Shen, L., et al (22) 12wk	0.420	0.176 to 1.000		33.53
	Zhao, D., et al (24)	0.540	0.292 to 1.000		66.47
	Total (random effects)	0.496	0.300 to 0.820	0.006	100.00
Heterogeneity	$I^2 = 0.00$ %, P = 0.6434				

Figure S42k. Forest plot for Complement component C6. GDM compared to controls.

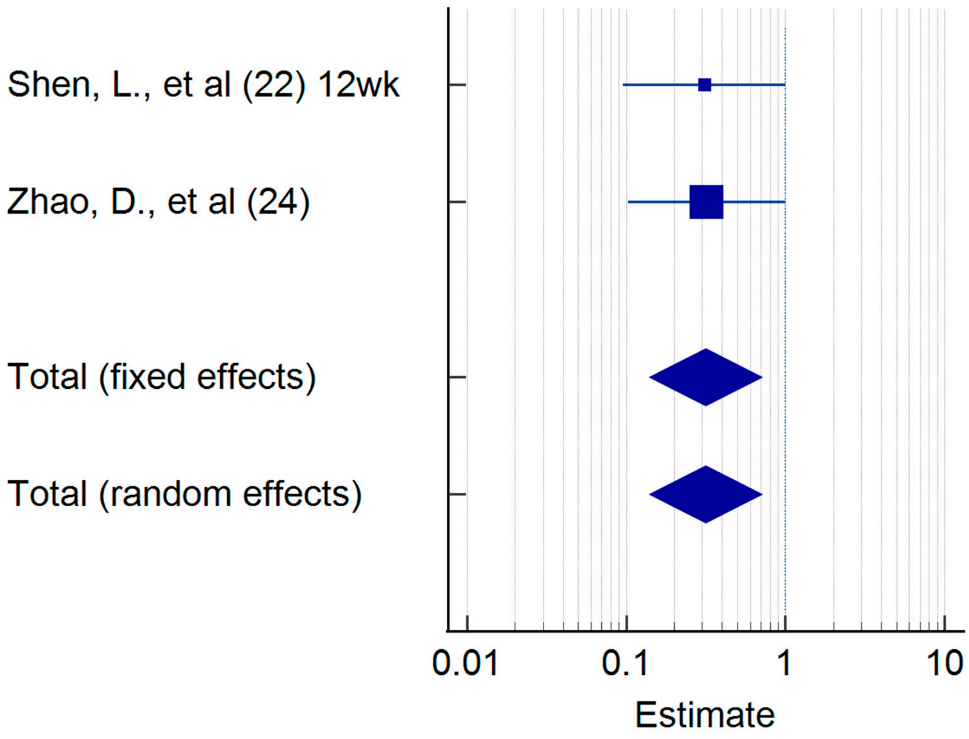

Protein	Study	RoM	95%CI	P	Weight (%)
Complement component C6	Shen, L., et al (22) 12wk	0.310	0.0961 to 1.000		48.63
	Zhao, D., et al (24)	0.320	0.102 to 1.000		51.37
	Total (random effects)	0.315	0.139 to 0.713	0.006	100.00
Heterogeneity	$I^2 = 0.00\%$, P = 0.9696				

Figure S42I. Forest plot for Complement component C7. GDM compared to controls.

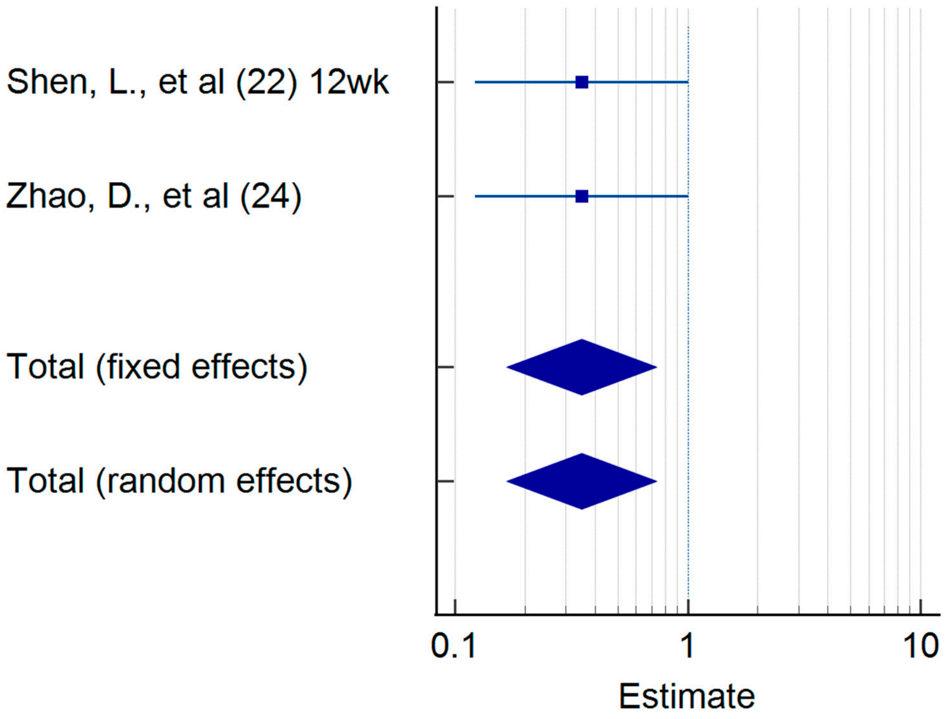

Protein	Study	RoM	95%CI	P	Weight (%)
Complement component C7	Shen, L., et al (22) 12wk	0.350	0.122 to 1.000		50.00
	Zhao, D., et al (24)	0.350	0.122 to 1.000		50.00
	Total (random effects)	0.350	0.167 to 0.735	0.006	100.00
Heterogeneity	$I^2 = 0.00$ %, P = 0.9798				

Figure S42m. Forest plot for Complement component C8 beta chain. GDM compared to controls.

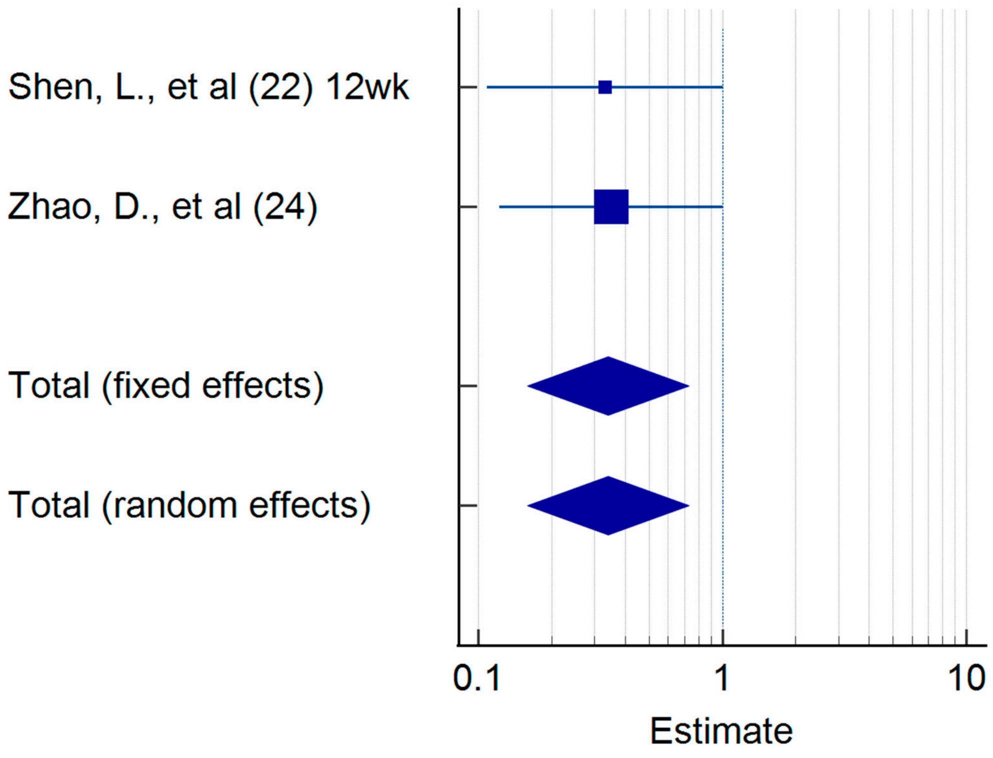

Protein	Study	RoM	95%CI	P	Weight (%)
Complement component C8 beta chain	Shen, L., et al (22) 12wk	0.330	0.109 to 1.000		47.28
	Zhao, D., et al (24)	0.350	0.122 to 1.000		52.72
	Total (random effects)	0.340	0.159 to 0.730	0.006	100.00
Heterogeneity	$I^2 = 0.00$ %, P = 0.9398				

Figure S42n. Forest plot for Complement component C8 gamma chain. GDM compared to controls.

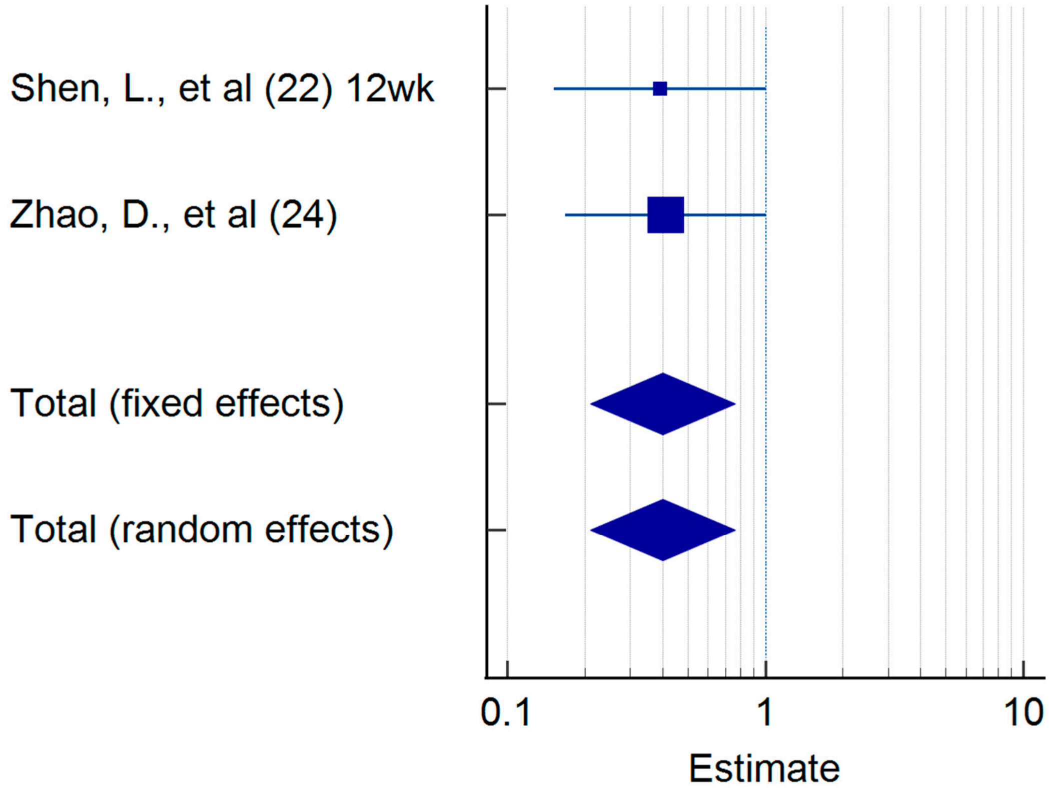

Protein	Study	RoM	95%CI	P	Weight (%)
Complement component C8 gamma chain	Shen, L., et al (22) 12wk	0.390	0.152 to 1.000		47.27
	Zhao, D., et al (24)	0.410	0.168 to 1.000		52.73
	Total (random effects)	0.400	0.210 to 0.765	0.006	100.00
Heterogeneity	$I^2 = 0.00$ %, P = 0.9397				

Figure S42o. Forest plot for Complement component C9. GDM compared to controls.

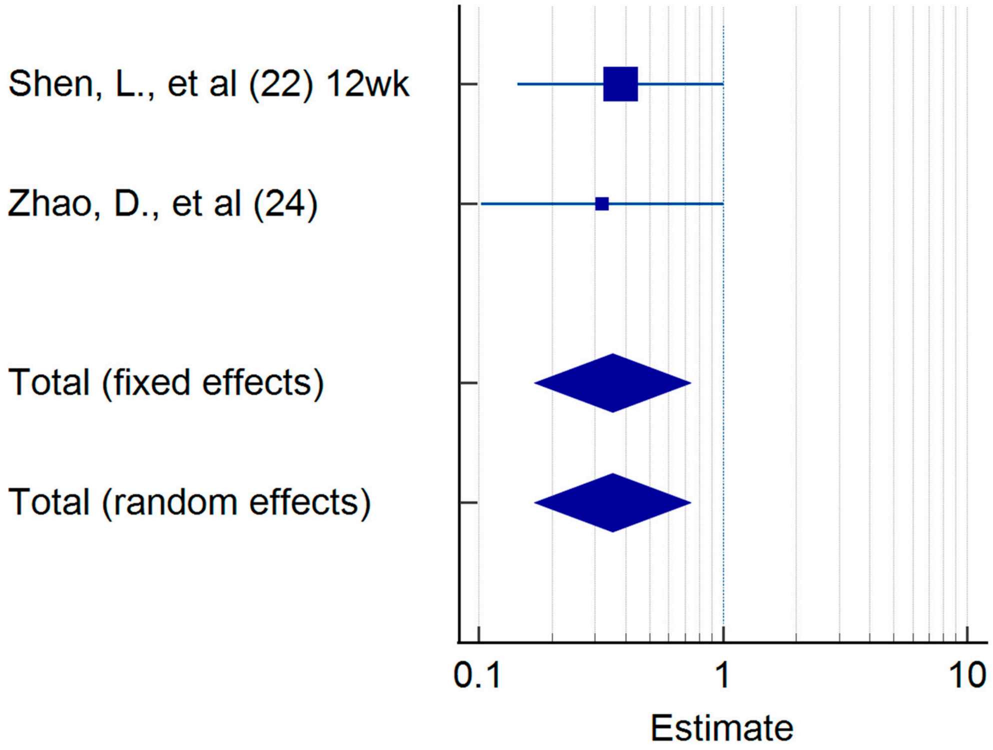

Protein	Study	RoM	95%CI	P	Weight (%)
Complement component C9	Shen, L., et al (22) 12wk	0.380	0.144 to 1.000		58.10
	Zhao, D., et al (24)	0.320	0.102 to 1.000		41.90
	Total (random effects)	0.354	0.169 to 0.739	0.006	100.00
Heterogeneity	$I^2 = 0.00$ %, P = 0.8217				

Figure S42p. Forest plot for Complement factor H. GDM compared to controls.

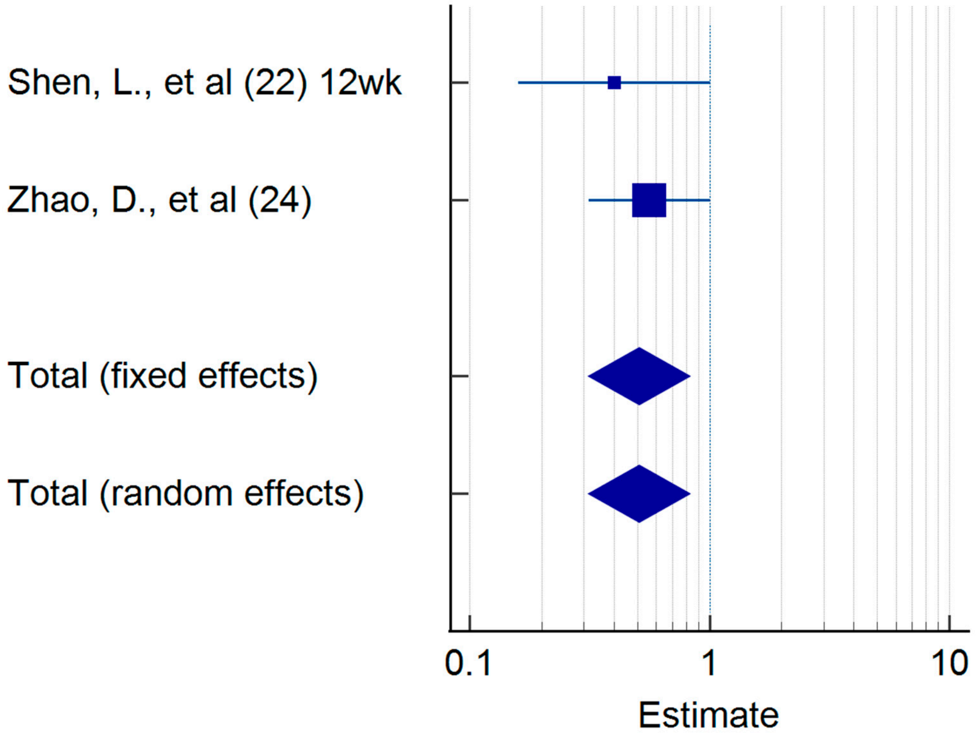

Protein	Study	RoM	95%CI	P	Weight (%)
Complement factor H	Shen, L., et al (22) 12wk	0.400	0.160 to 1.000		28.59
	Zhao, D., et al (24)	0.560	0.314 to 1.000		71.41
	Total (random effects)	0.509	0.312 to 0.830	0.007	100.00
Heterogeneity	$I^2 = 0.00$ %, P = 0.5431				

Figure S42q. Forest plot for Endoplasmin. GDM compared to controls.

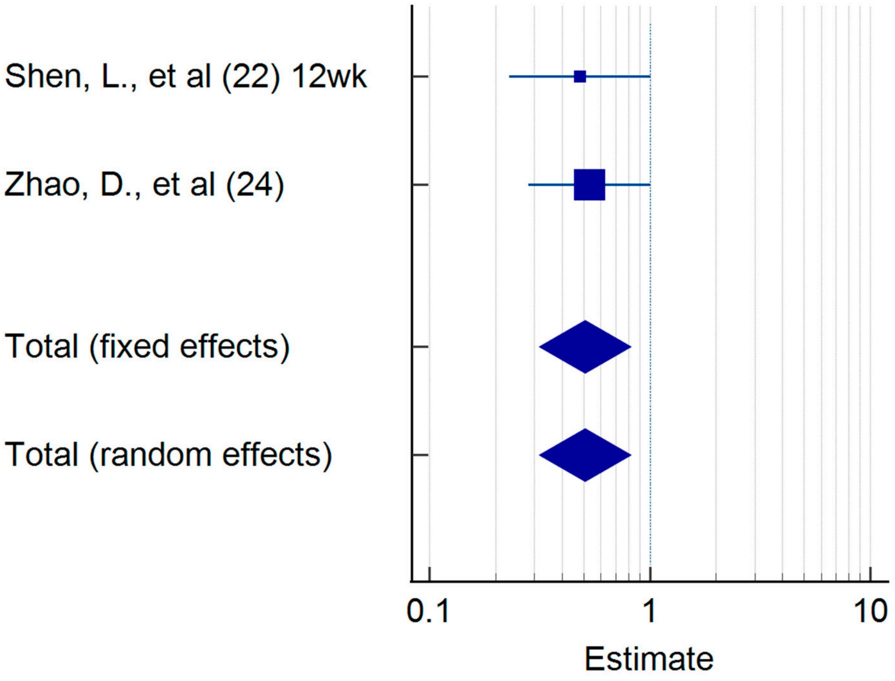

Protein	Study	RoM	95%CI	P	Weight (%)
Ensoplasmin	Shen, L., et al (22) 12wk	0.480	0.230 to 1.000		42.80
	Zhao, D., et al (24)	0.530	0.281 to 1.000		57.20
	Total (random effects)	0.508	0.314 to 0.821	0.006	100.00
Heterogeneity	$I^2 = 0.00$ %, P = 0.8414				

Figure S42r. Forest plot for Gelsolin. GDM compared to controls.

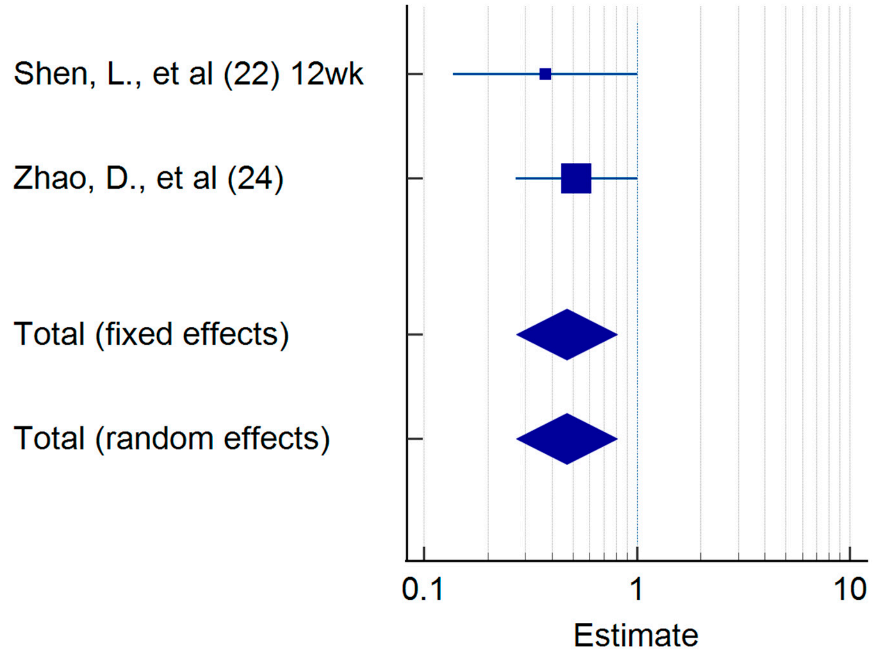

Protein	Study	RoM	95%CI	P	Weight (%)
Gelsolin	Shen, L., et al (22) 12wk	0.370	0.137 to 1.000		30.20
	Zhao, D., et al (24)	0.520	0.270 to 1.000		69.80
	Total (random effects)	0.469	0.272 to 0.810	0.007	100.00
Heterogeneity	$I^2 = 0.00$ %, P = 0.5751				

Figure S42s. Forest plot for Prothrombin. GDM compared to controls.

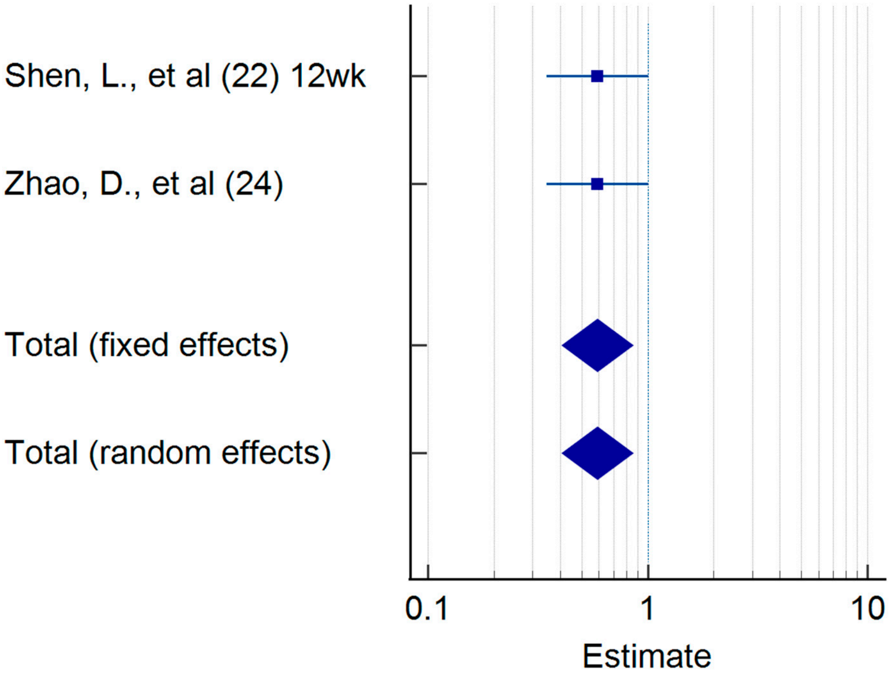

Protein	Study	RoM	95%CI	P	Weight (%)
Prothrombin	Shen, L., et al (22) 12wk	0.590	0.348 to 1.000		50.00
	Zhao, D., et al (24)	0.590	0.348 to 1.000		50.00
	Total (random effects)	0.590	0.406 to 0.857	0.006	100.00
Heterogeneity	$I^2 = 0.00$ %, P = 0.5656				
