# Supplementary material for: Proteomics Studies in Gestational Diabetes Mellitus: A Systematic Review and Meta-Analysis
Source: J Clin Med. 2022 May 12;11(10):2737. doi: 10.3390/jcm11102737 (PMC9143836; doi:10.3390/jcm11102737)
Supplement: Supplementary file 1 [file jcm-11-02737-s001.zip › jcm-1695841-SI/Supplementary Figure 43.pdf]

Figure S43a. Forest plot for Fibrinogen alpha chain. GDM compared to controls.

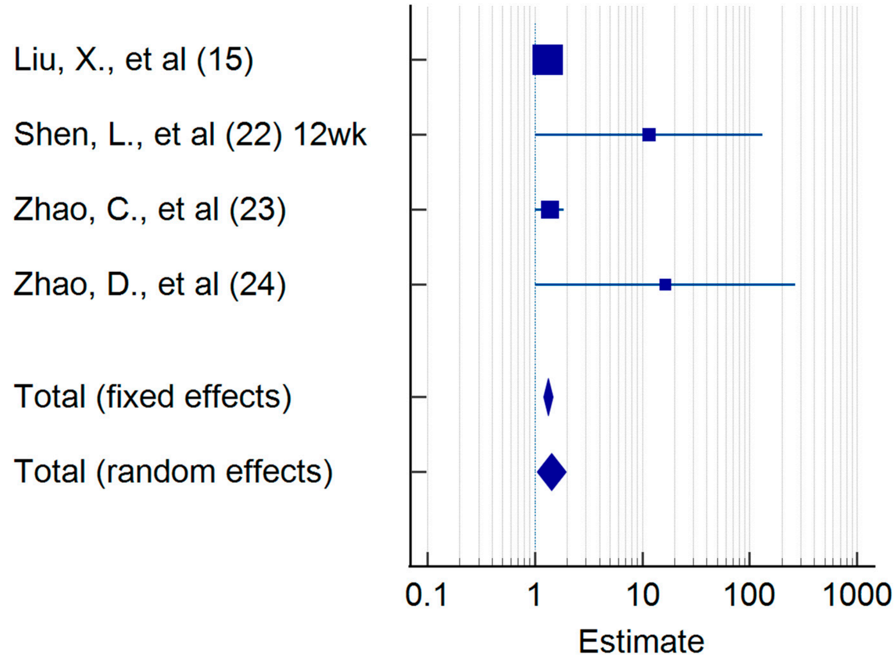

Protein	Study	RoM	95%CI	P	Weight (%)
Fibrinogen alpha chain	Liu, X., et al (15)	1.316	1.181 to 1.465		57.80
	Shen, L., et al (22) 12wk	11.550	1.000 to 133.408		1.61
	Zhao, C., et al (23)	1.370	1.011 to 1.856		39.34
	Zhao, D., et al (24)	16.330	1.000 to 266.683		1.25
	Total (random effects)	1.429	1.043 to 1.957	0.026	100.00
Heterogeneity	$I^2 = 51.38$ %, P = 0.1036				

Figure S43b. Forest plot for Proteoglycan 4. GDM compared to controls.

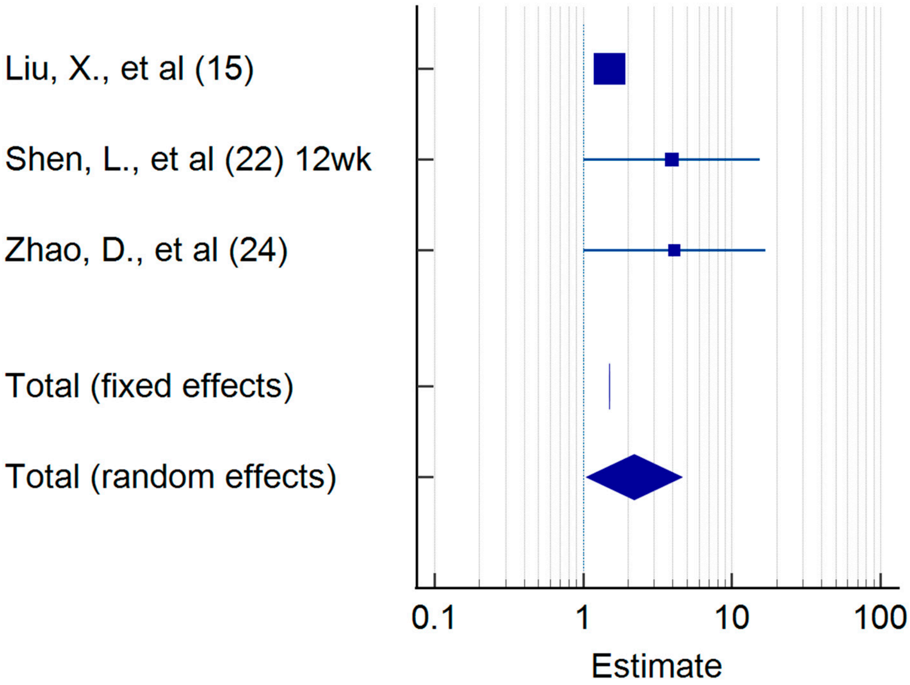

Protein	Study	RoM	95%CI	P	Weight (%)
Proteoglycan 4	Liu, X., et al (15)	1.498	1.495 to 1.501		61.10
	Shen, L., et al (22) 12wk	3.940	1.000 to 15.524		19.85
	Zhao, D., et al (24)	4.110	1.000 to 16.893		19.05
	Total (random effects)	2.200	1.046 to 4.628	0.038	100.00
Heterogeneity	$I^2 = 48.30$ %, P = 0.1445				

Figure S43c. Forest plot for Ig mu chain C region. GDM compared to controls.

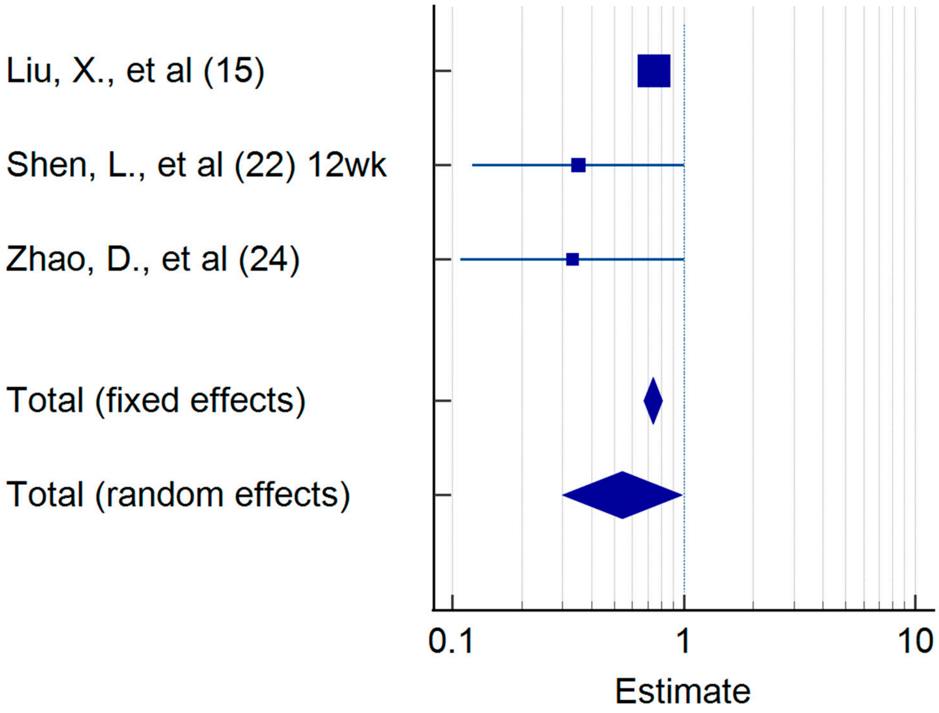

Protein	Study	RoM	95%CI	P	Weight (%)
Ig mu chain C region	Liu, X., et al (15)	0.745	0.679 to 0.818		59.44
	Shen, L., et al (22) 12wk	0.350	0.122 to 1.000		21.01
	Zhao, D., et al (24)	0.330	0.109 to 1.000		19.55
	Total (random effects)	0.542	0.299 to 0.984	0.044	100.00
Heterogeneity	$I^2 = 50.08$ %, P = 0.1349				
