# Supplementary material for: Proteomics Studies in Gestational Diabetes Mellitus: A Systematic Review and Meta-Analysis
Source: J Clin Med. 2022 May 12;11(10):2737. doi: 10.3390/jcm11102737 (PMC9143836; doi:10.3390/jcm11102737)
Supplement: Supplementary file 1 [file jcm-11-02737-s001.zip › jcm-1695841-SI/Supplementary Figure 44.pdf]

Figure S44a. Forest plot for Alpha-1-antitripsin. GDM compared to controls.

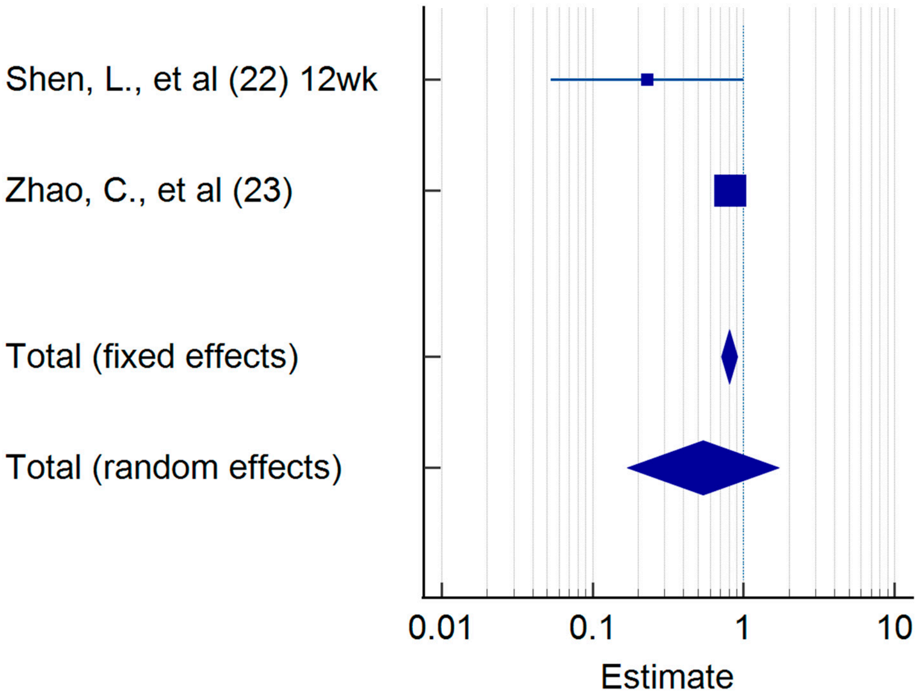

Protein	Study	RoM	95%CI	P	Weight (%)
Alpha-1-antitripsin	Shen, L., et al (22) 12wk	0.230	0.0529 to 1.000		32.49
	Zhao, C., et al (23)	0.813	0.719 to 0.920		67.51
	Total (random effects)	0.539	0.169 to 1.719	0.297	100.00
Heterogeneity	I^2 = 64.48 %, P = 0.0933				

Figure S44b. Forest plot for C-reactive protein. GDM compared to controls.

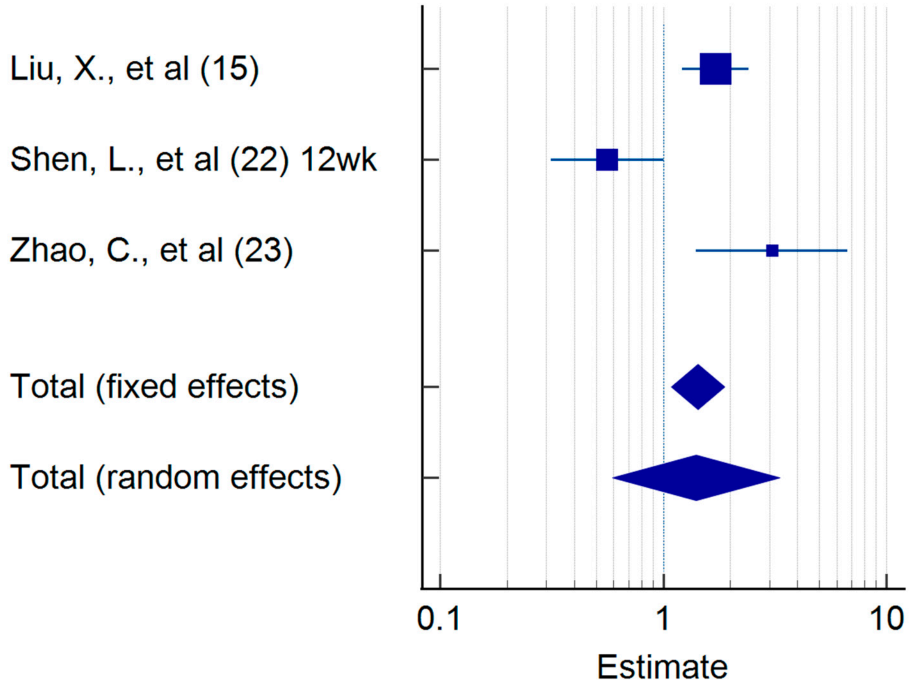

Protein	Study	RoM	95%CI	P	Weight (%)
C-reactive protein	Liu, X., et al (15)	1.714	1.217 to 2.415		36.97
	Shen, L., et al (22) 12wk	0.560	0.314 to 1.000		33.35
	Zhao, C., et al (23)	3.070	1.403 to 6.719		29.68
	Total (random effects)	1.403	0.591 to 3.329	0.442	100.00
Heterogeneity	I^2 = 86.46 %, P = 0.0006				

Figure S44c. Forest plot for Pregnancy zone protein. GDM compared to controls.

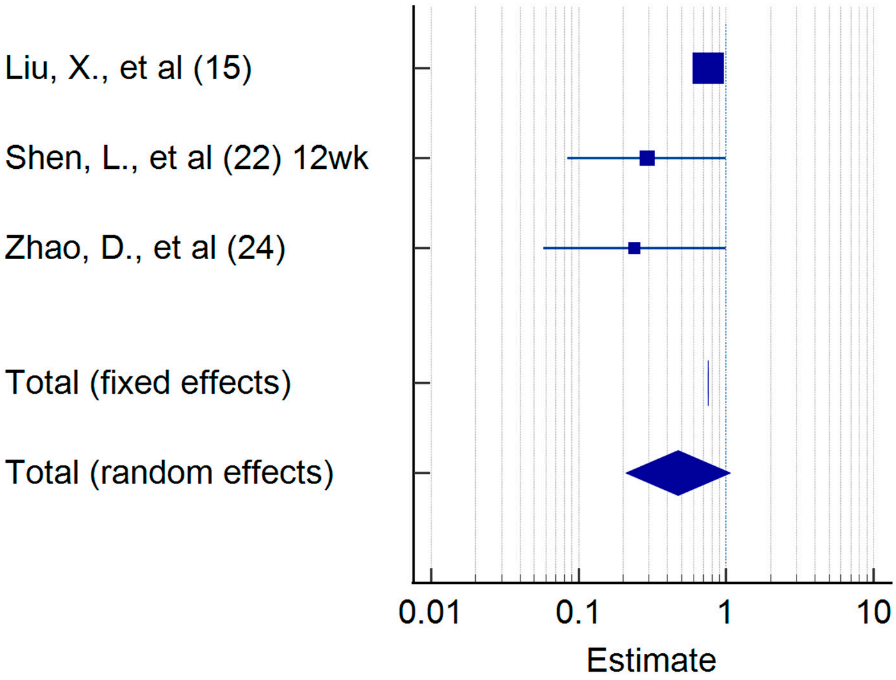

Protein	Study	RoM	95%CI	P	Weight (%)
Pregnancy zone protein	Liu, X., et al (15)	0.755	0.753 to 0.756		55.11
	Shen, L., et al (22) 12wk	0.290	0.0841 to 1.000		24.34
	Zhao, D., et al (24)	0.240	0.0576 to 1.000		20.56
	Total (random effects)	0.473	0.209 to 1.070	0.072	100.00
Heterogeneity	I^2 = 58.09 %, P = 0.0920				
