# Supplementary material for: Proteomics Studies in Gestational Diabetes Mellitus: A Systematic Review and Meta-Analysis
Source: J Clin Med. 2022 May 12;11(10):2737. doi: 10.3390/jcm11102737 (PMC9143836; doi:10.3390/jcm11102737)
Supplement: Supplementary file 1 [file jcm-11-02737-s001.zip › jcm-1695841-SI/Supplementary Figures 1 to 40.pdf]

Supplementary Figure S1. Forest plot for Apolipoprotein A-V. GDM compared to controls.

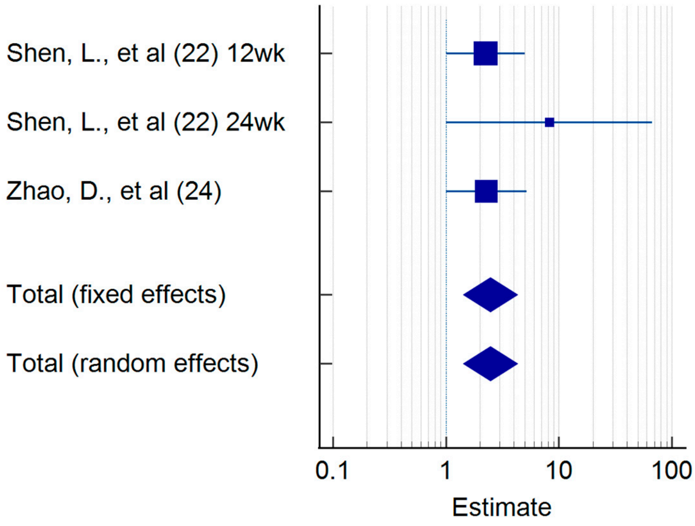

Protein	Study	RoM	95% CI	P	Weight (%)
Apolipoprotein A-V	Shen, L., et al (22) 12wk	2.240	1.000 to 5.018		47.52
	Shen, L., et al (22) 24wk	8.200	1.000 to 67.243		6.98
	Zhao, D., et al (24)	2.280	1.000 to 5.198		45.50
	Total (random effects)	2.472	1.418 to 4.311	0.001	100.00
Heterogeneity	I^2 = 0.00%, P = 0.5112				

Supplementary Figure S2. Forest plot for Apolipoprotein C-III. GDM compared to controls.

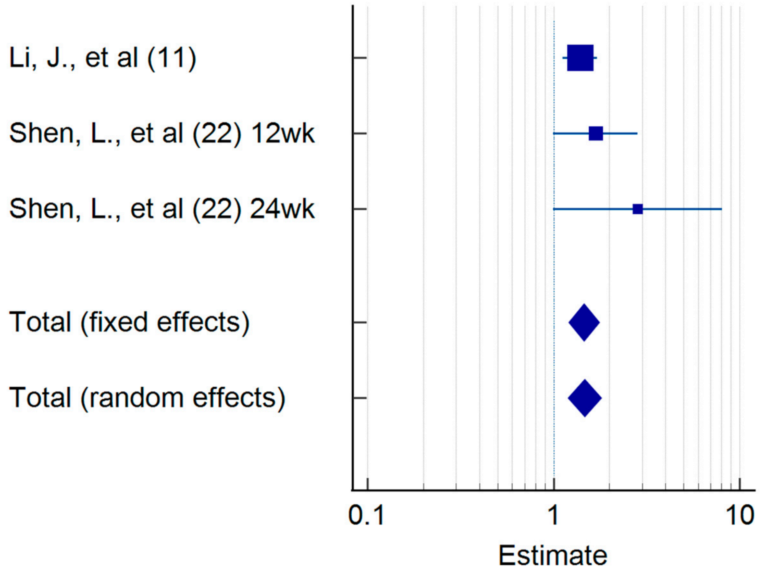

Protein	Study	RoM	95% CI	P	Weight (%)
Apolipoprotein C-III	Li, J., et al (11)	1.386	1.123 to 1.710		80.44
	Shen, L., et al (22) 12wk	1.680	1.000 to 2.822		15.60
	Shen, L., et al (22) 24wk	2.840	1.000 to 8.066		3.96
	Total (random effects)	1.469	1.192 to 1.810	<0.001	100.00
Heterogeneity	I^2 = 3.83%, P = 0.3535				

Supplementary Figure S3. Forest plot for Apolipoprotein E. GDM compared to controls.

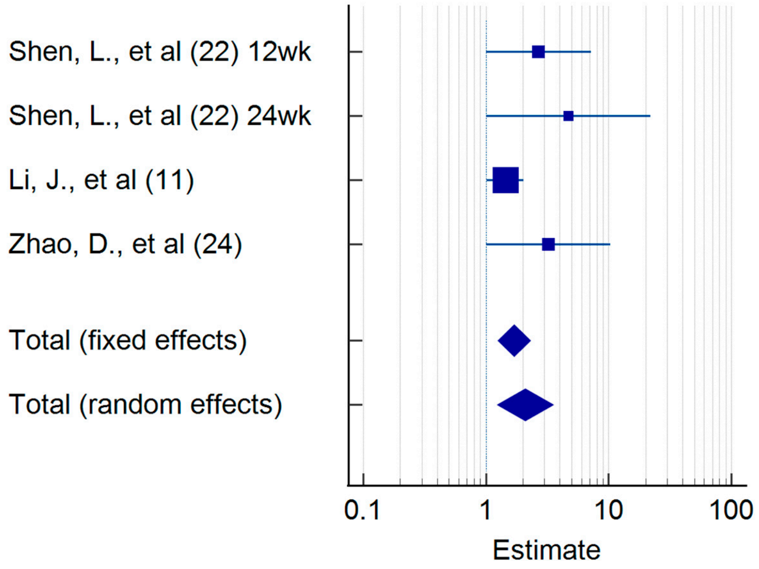

Protein	Study	RoM	95% CI	P	Weight (%)
Apolipoprotein E	Shen, L., et al (22) 12wk	2.680	1.000 to 7.183		20.35
	Shen, L., et al (22) 24wk	4.690	1.000 to 21.997		10.01
	Li, J., et al (11)	1.441	1.020 to 2.036		53.77
	Zhao, D., et al (24)	3.210	1.000 to 10.304		15.86
	Total (random effects)	2.089	1.232 to 3.543	0.006	100.00
Heterogeneity	I^2 = 33.29%, P = 0.2126				

Supplementary Figure S4. Forest plot for Coagulation factor IX. GDM compared to controls.

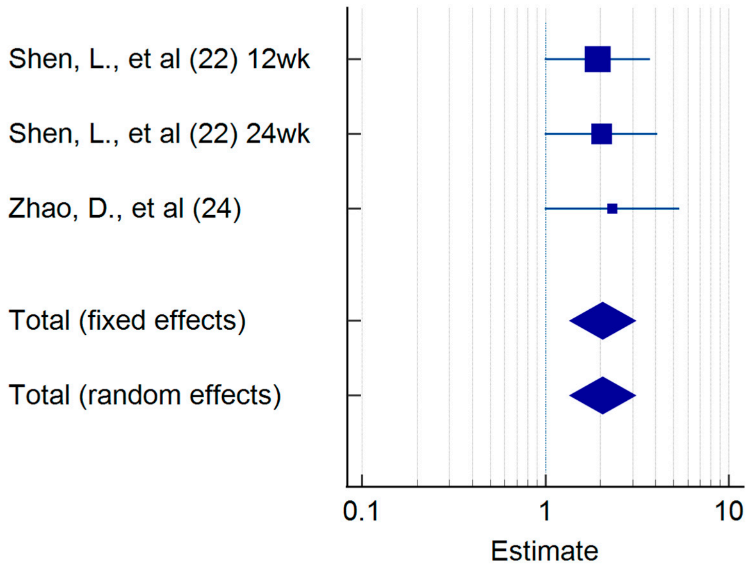

Protein	Study	RoM	95% CI	P	Weight (%)
Coagulation factor IX	Shen, L., et al (22) 12wk	1.930	1.000 to 3.725		40.24
	Shen, L., et al (22) 24wk	2.020	1.000 to 4.080		35.19
	Zhao, D., et al (24)	2.320	1.000 to 5.382		24.56
	Total (random effects)	2.052	1.352 to 3.114	0.001	100.00
Heterogeneity	I^2 = 0.00%, P = 0.9432				

Supplementary Figure S5. Forest plot for Coagulation factor X. GDM compared to controls.

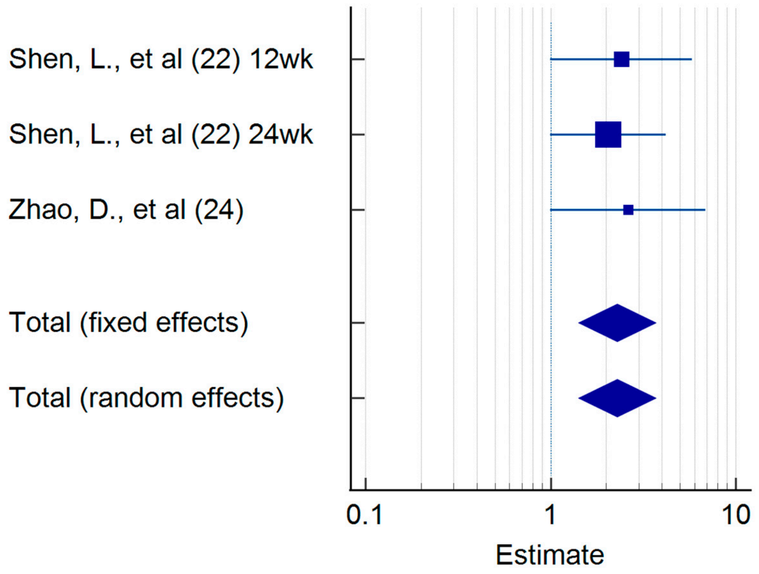

Protein	Study	RoM	95% CI	P	Weight (%)
Coagulation factor X	Shen, L., et al (22) 12wk	2.410	1.000 to 5.808		29.98
	Shen, L., et al (22) 24wk	2.050	1.000 to 4.203		45.02
	Zhao, D., et al (24)	2.620	1.000 to 6.865		25.00
	Total (random effects)	2.288	1.413 to 3.704	0.001	100.00
Heterogeneity	I^2 = 0.00%, P = 0.9142				

Supplementary Figure S6. Forest plot for Coagulation factor XII. GDM compared to controls.

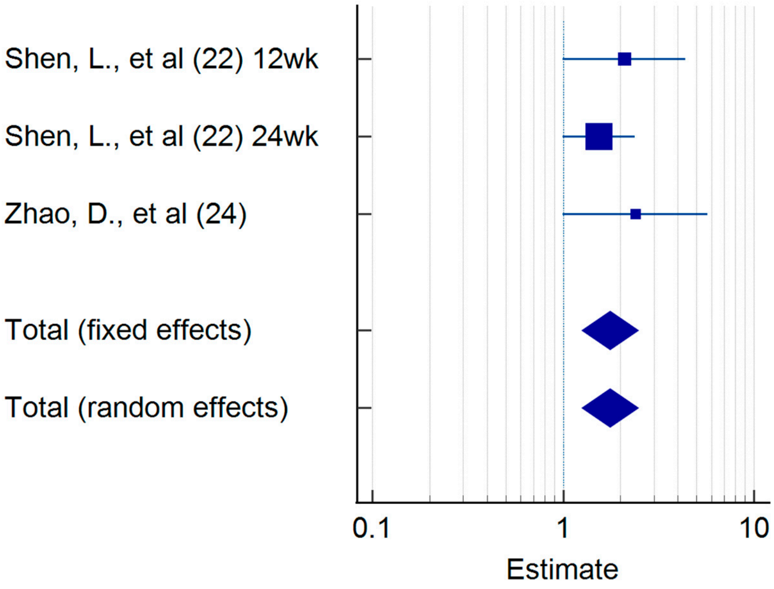

Protein	Study	RoM	95% CI	P	Weight (%)
Coagulation factor XII	Shen, L., et al (22) 12wk	2.090	1.000 to 4.368		21.60
	Shen, L., et al (22) 24wk	1.540	1.000 to 2.372		62.95
	Zhao, D., et al (24)	2.390	1.000 to 5.712		15.46
	Total (random effects)	1.761	1.250 to 2.480	0.001	100.00
Heterogeneity	I^2 = 0.00%, P = 0.5916				

Supplementary Figure S7. Forest plot for Complement C1s subcomponent**.** GDM compared to controls.

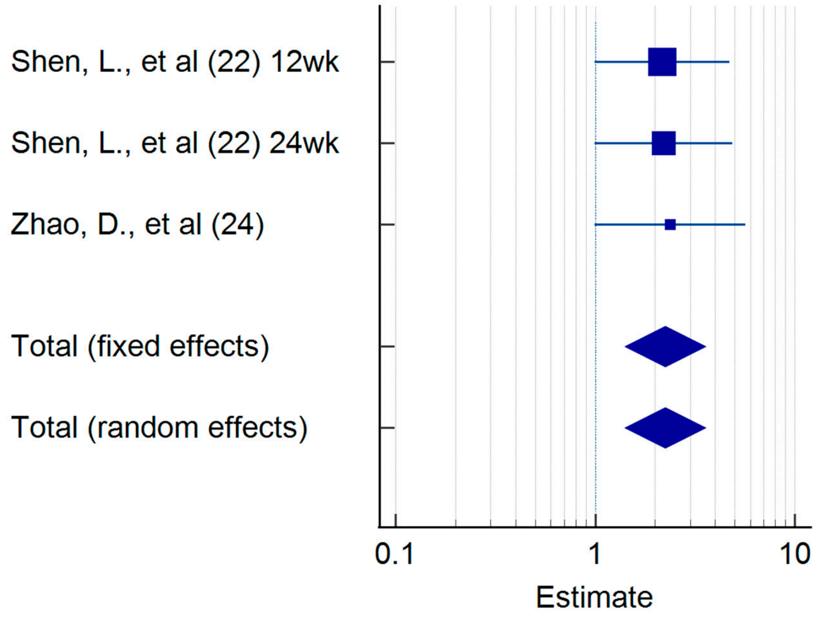

Protein	Study	RoM	95% CI	P	Weight (%)
Complement C1s subcomponent	Shen, L., et al (22) 12wk	2.170	1.000 to 4.709		36.33
	Shen, L., et al (22) 24wk	2.210	1.000 to 4.884		34.67
	Zhao, D., et al (24)	2.380	1.000 to 5.664		29.00
	Total (random effects)	2.243	1.406 to 3.578	0.001	100.00
Heterogeneity	I^2 = 0.00%, P = 0.9869				

Supplementary Figure S8. Forest plot for Proteoglycan 4. GDM compared to controls.

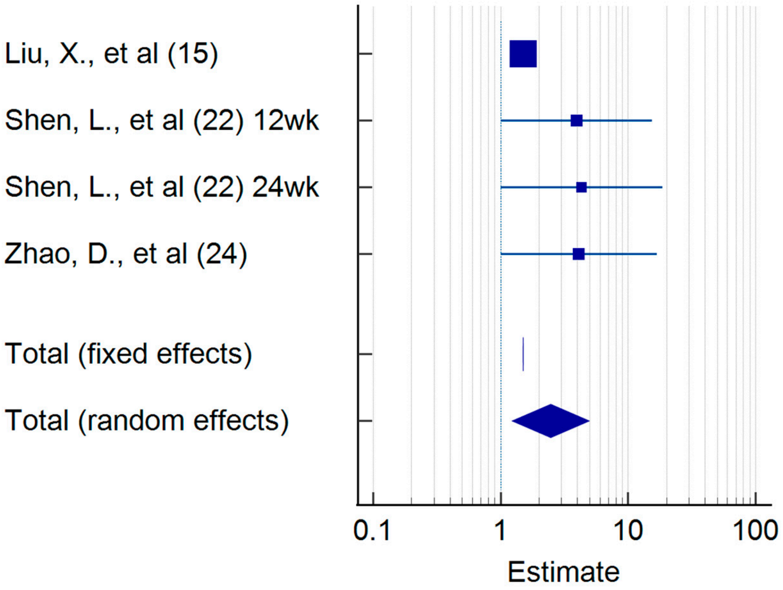

Protein	Study	RoM	95% CI	P	Weight (%)
Proteoglycan 4	Liu, X., et al (15)	1.498	1.495 to 1.501		50.70
	Shen, L., et al (22) 12wk	3.940	1.000 to 15.524		17.14
	Shen, L., et al (22) 24wk	4.320	1.000 to 18.663		15.70
	Zhao, D., et al (24)	4.110	1.000 to 16.893		16.46
	Total (random effects)	2.466	1.227 to 4.954	0.011	100.00
Heterogeneity	I^2 = 48.99%, P = 0.1176				

Supplementary Figure S9. Forest plot for Serum amyloid P-component**.** GDM compared to controls.

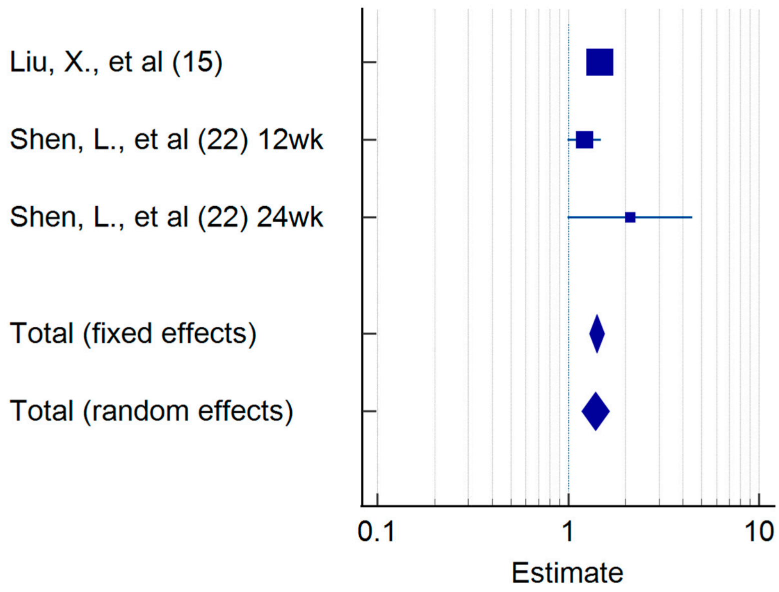

Protein	Study	RoM	95% CI	P	Weight (%)
Serum amyloid P-component	Liu, X., et al (15)	1.460	1.318 to 1.618		58.73
	Shen, L., et al (22) 12wk	1.220	1.000 to 1.488		36.59
	Shen, L., et al (22) 24wk	2.120	1.000 to 4.494		4.68
	Total (random effects)	1.391	1.176 to 1.645	<0.001	100.00
Heterogeneity	I^2 = 44.50%, P = 0.1650				

Supplementary Figure S10. Forest plot for C4b-binding protein alpha chain. GDM compared to controls.

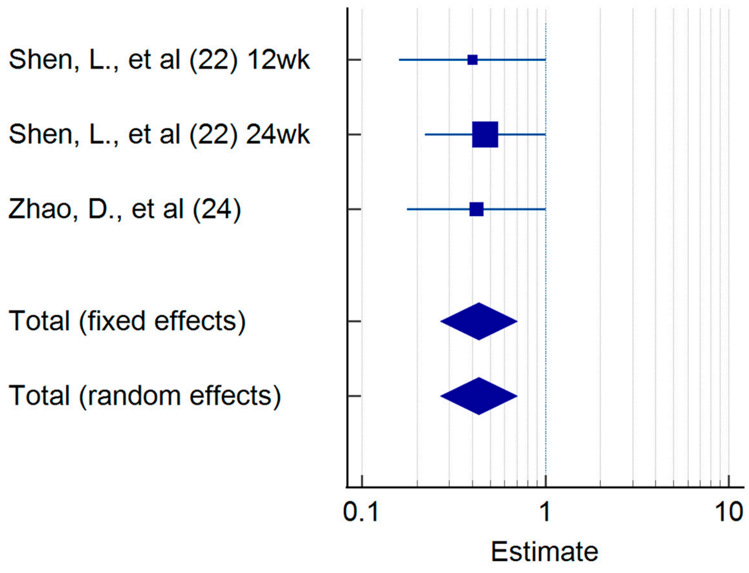

Protein	Study	RoM	95% CI	P	Weight (%)
C4b-binding protein alpha chain	Shen, L., et al (22) 12wk	0.400	0.160 to 1.000		27.87
	Shen, L., et al (22) 24wk	0.470	0.221 to 1.000		41.04
	Zhao, D., et al (24)	0.420	0.176 to 1.000		31.09
	Total (random effects)	0.434	0.267 to 0.704	0.001	100.00
Heterogeneity	I^2 = 0.00%, P = 0.9614				

Supplementary Figure S11. Forest plot for Complement component C6**.** GDM compared to controls.

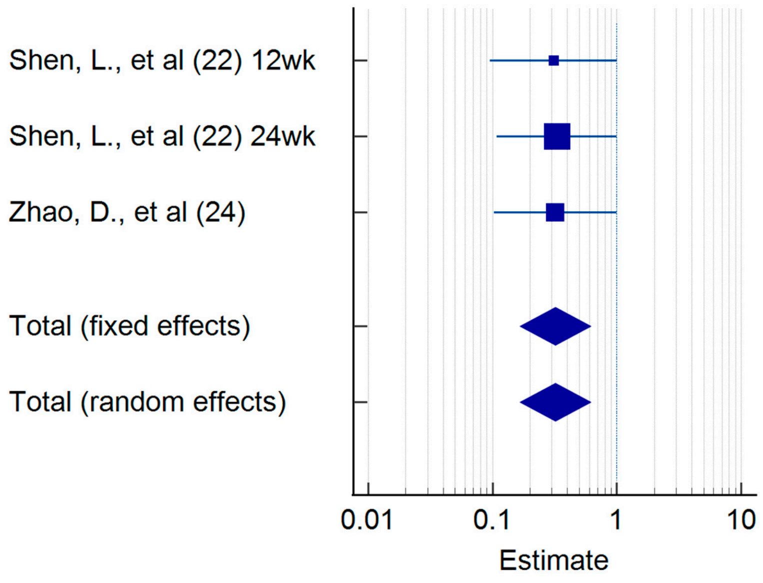

Protein	Study	RoM	95% CI	P	Weight (%)
Complement component C6	Shen, L., et al (22) 12wk	0.310	0.0961 to 1.000		31.52
	Shen, L., et al (22) 24wk	0.330	0.109 to 1.000		35.18
	Zhao, D., et al (24)	0.320	0.102 to 1.000		33.30
	Total (random effects)	0.320	0.166 to 0.618	0.001	100.00
Heterogeneity	$I^2 = 0.00\%$, P = 0.9971				

Supplementary Figure S12. Forest plot for Complement component C7. GDM compared to controls.

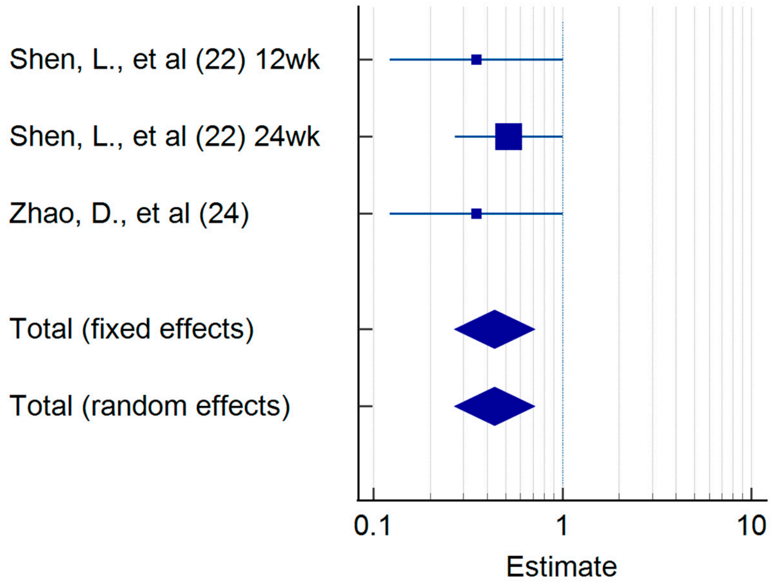

Protein	Study	RoM	95% CI	P	Weight (%)
Complement component C7	Shen, L., et al (22) 12wk	0.350	0.122 to 1.000		21.85
	Shen, L., et al (22) 24wk	0.520	0.270 to 1.000		56.31
	Zhao, D., et al (24)	0.350	0.122 to 1.000		21.85
	Total (random effects)	0.437	0.268 to 0.714	0.001	100.00
Heterogeneity	I^2 = 0.00%, P = 0.7352				

Supplementary Figure S13. Forest plot for Complement component C8 beta chain. GDM compared to controls.

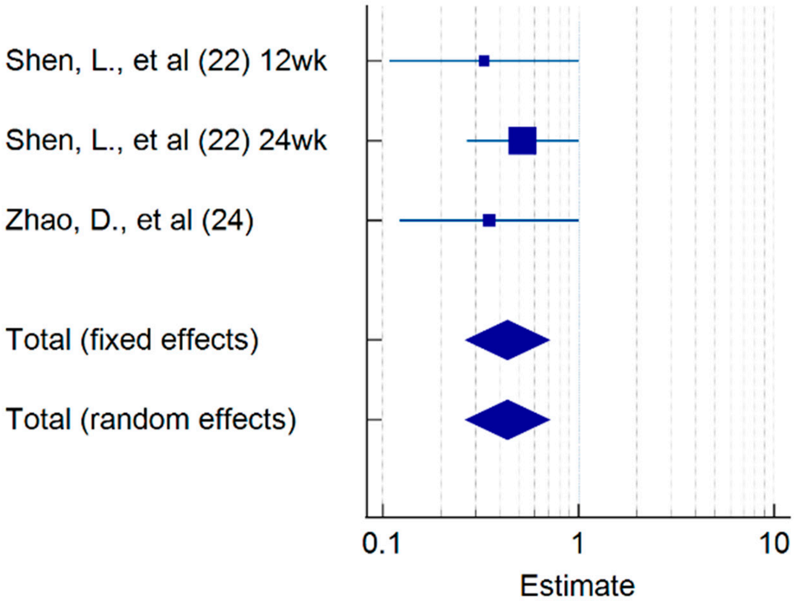

Protein	Study	RoM	95% CI	P	Weight (%)
Complement component C8 beta chain	Shen, L., et al (22) 12wk	0.330	0.109 to 1.000		20.04
	Shen, L., et al (22) 24wk	0.520	0.270 to 1.000		57.61
	Zhao, D., et al (24)	0.350	0.122 to 1.000		22.35
	Total (random effects)	0.435	0.265 to 0.714	0.001	100.00
Heterogeneity	I^2 = 0.00%, P = 0.7084				

Supplementary Figure S14. Forest plot for Complement component C8 gamma chain. GDM compared to controls.

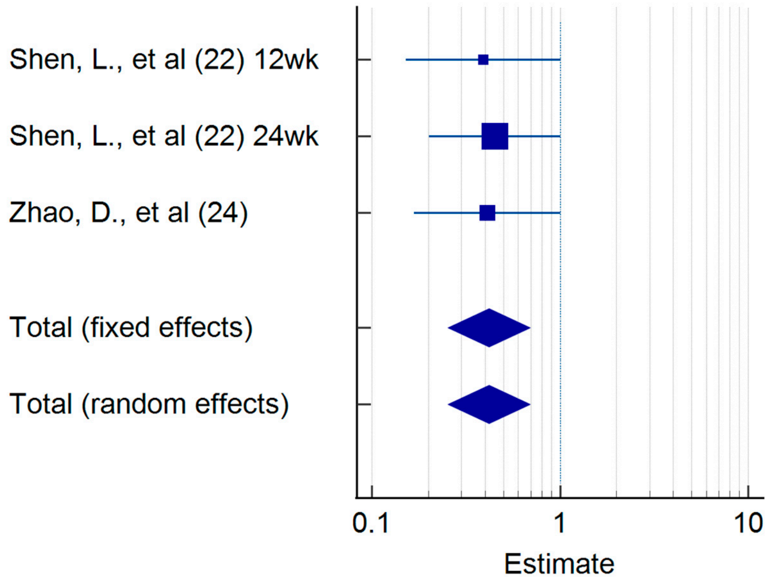

Protein	Study	RoM	95% CI	P	Weight (%)
Complement component C8 gamma chain	Shen, L., et al (22) 12wk	0.390	0.152 to 1.000		28.52
	Shen, L., et al (22) 24wk	0.450	0.202 to 1.000		39.66
	Zhao, D., et al (24)	0.410	0.168 to 1.000		31.81
	Total (random effects)	0.419	0.254 to 0.693	0.001	100.00
Heterogeneity	I^2 = 0.00%, P = 0.9728				

Supplementary Figure S15. Forest plot for Complement component C9**.** GDM compared to controls.

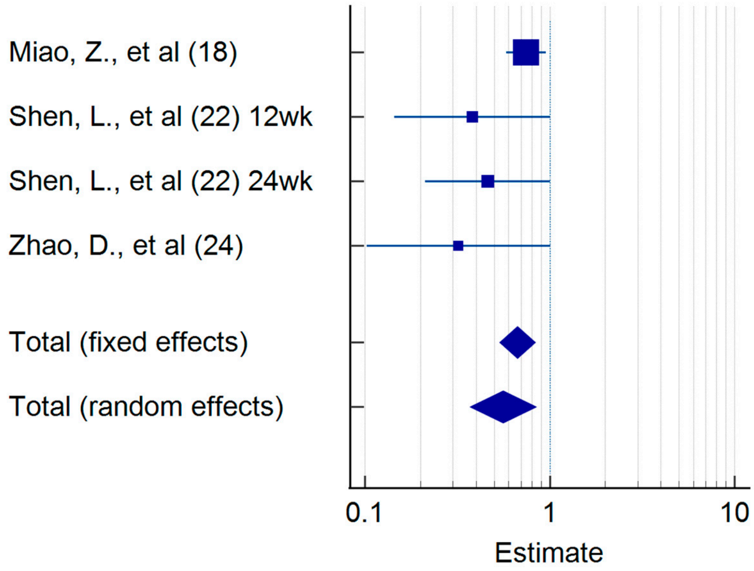

Protein	Study	RoM	95% CI	P	Weight (%)
Complement component C9	Miao, Z., et al (18)	0.746	0.583 to 0.954		54.54
	Shen, L., et al (22) 12wk	0.380	0.144 to 1.000		14.41
	Shen, L., et al (22) 24wk	0.460	0.212 to 1.000		20.01
	Zhao, D., et al (24)	0.320	0.102 to 1.000		11.04
	Total (random effects)	0.560	0.370 to 0.846	0.006	100.00
Heterogeneity	I^2 = 34.22%, P = 0.2070				

Supplementary Figure S16. Forest plot for Complement factor H. GDM compared to controls.

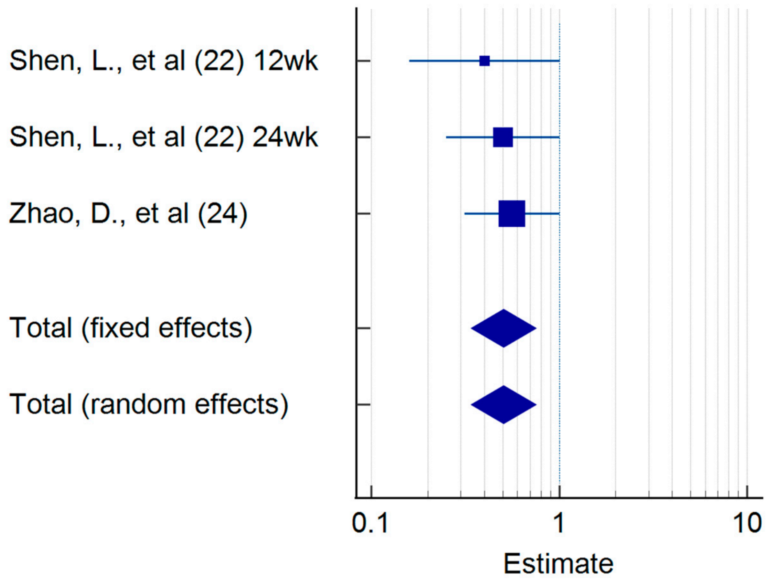

Protein	Study	RoM	95% CI	P	Weight (%)
Complement factor H	Shen, L., et al (22) 12wk	0.400	0.160 to 1.000		19.07
	Shen, L., et al (22) 24wk	0.500	0.250 to 1.000		33.32
	Zhao, D., et al (24)	0.560	0.314 to 1.000		47.62
	Total (random effects)	0.506	0.339 to 0.755	0.001	100.00
Heterogeneity	I^2 = 0.00%, P = 0.8305				

Supplementary Figure S17. Forest plot for Endoplasmin. GDM compared to controls.

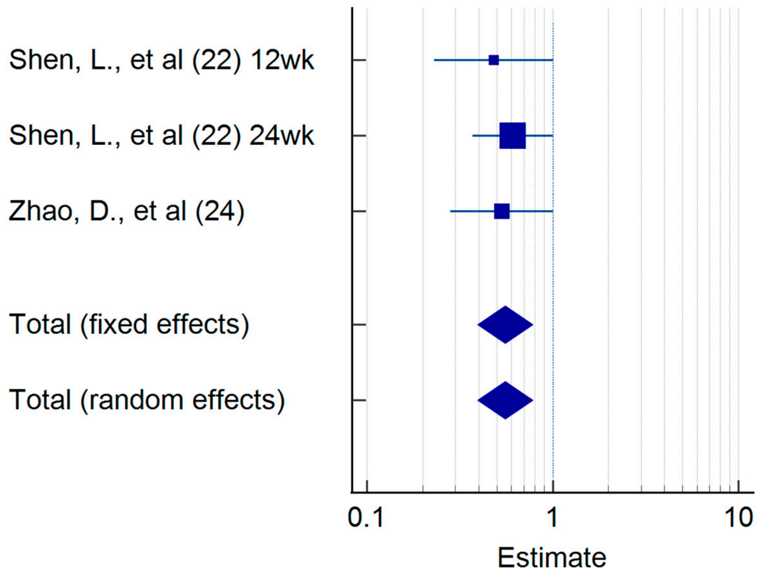

Protein	Study	RoM	95% CI	P	Weight (%)
Endoplasmin	Shen, L., et al (22) 12wk	0.480	0.230 to 1.000		22.02
	Shen, L., et al (22) 24wk	0.610	0.372 to 1.000		48.55
	Zhao, D., et al (24)	0.530	0.281 to 1.000		29.43
	Total (random effects)	0.555	0.393 to 0.783	0.001	100.00
Heterogeneity	I^2 = 0.00%, P = 0.8560				

Supplementary Figure S18. Forest plot for Gelsolin**.** GDM compared to controls.

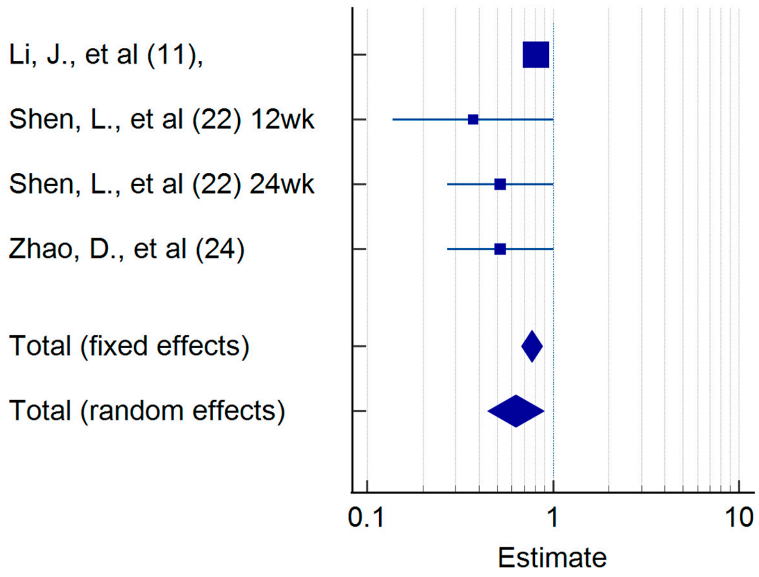

Protein	Study	RoM	95% CI	P	Weight (%)
Gelsolin	Li, J., et al (11),	0.809	0.704 to 0.929		51.61
	Shen, L., et al (22) 12wk	0.370	0.137 to 1.000		10.23
	Shen, L., et al (22) 24wk	0.520	0.270 to 1.000		19.08
	Zhao, D., et al (24)	0.520	0.270 to 1.000		19.08
	Total (random effects)	0.631	0.444 to 0.897	0.010	100.00
Heterogeneity	I^2 = 43.83%, P = 0.1485				

Supplementary Figure S19. Forest plot for Ig mu chain C region**.** GDM compared to controls.

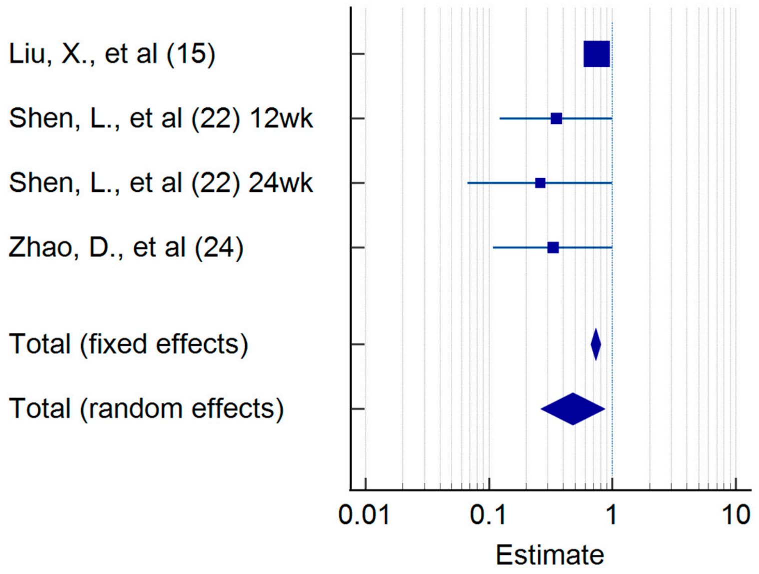

Protein	Study	RoM	95% CI	P	Weight (%)
Ig mu chain C region	Liu, X., et al (15)	0.745	0.679 to 0.818		48.21
	Shen, L., et al (22) 12wk	0.350	0.122 to 1.000		19.50
	Shen, L., et al (22) 24wk	0.260	0.0676 to 1.000		14.05
	Zhao, D., et al (24)	0.330	0.109 to 1.000		18.24
	Total (random effects)	0.478	0.263 to 0.870	0.016	100.00
Heterogeneity	I^2 = 52.33%, P = 0.0982				

Supplementary Figure S20. Forest plot for Pappalysin-1**.** GDM compared to controls.

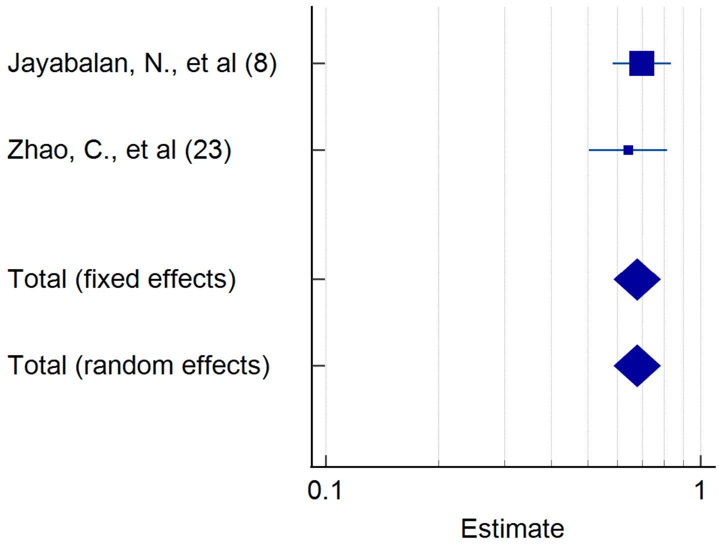

Protein	Study	RoM	95% CI	P	Weight (%)
Pappalysin-1	Jayabalan, N., et al (8)	0.697	0.583 to 0.834		64.46
	Zhao, C., et al (23)	0.641	0.504 to 0.816		35.54
	Total (random effects)	0.677	0.586 to 0.781	<0.001	100.00
Heterogeneity	I^2 = 0.00%, P = 0.5825				

Supplementary Figure S21. Forest plot for Secreted phosphoprotein 24. GDM compared to controls.

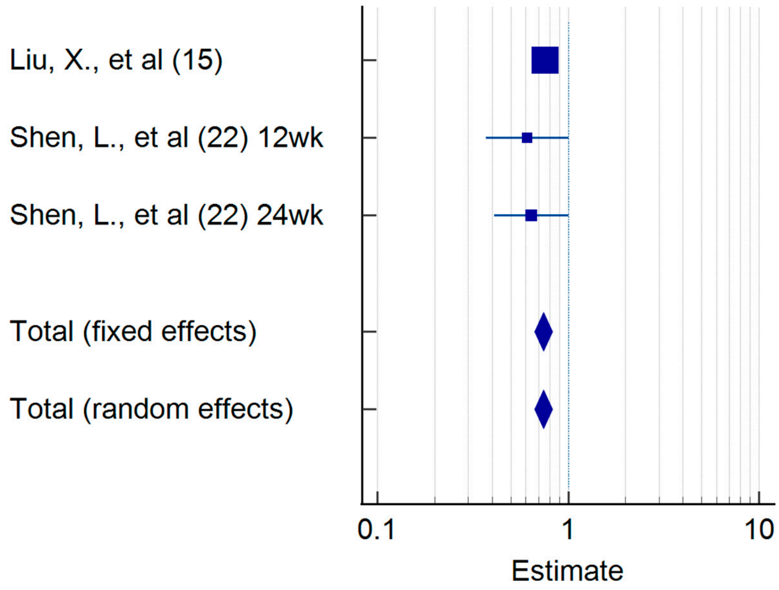

Protein	Study	RoM	95% CI	P	Weight (%)
Secreted phosphoprotein 24	Liu, X., et al (15)	0.757	0.676 to 0.849		89.44
	Shen, L., et al (22) 12wk	0.610	0.372 to 1.000		4.74
	Shen, L., et al (22) 24wk	0.640	0.410 to 1.000		5.82
	Total (random effects)	0.742	0.667 to 0.827	<0.001	100.00
Heterogeneity	I^2 = 0.00%, P = 0.5630				

Supplementary Figure S22. Forest plot for Sex hormone-binding globulin. GDM compared to controls.

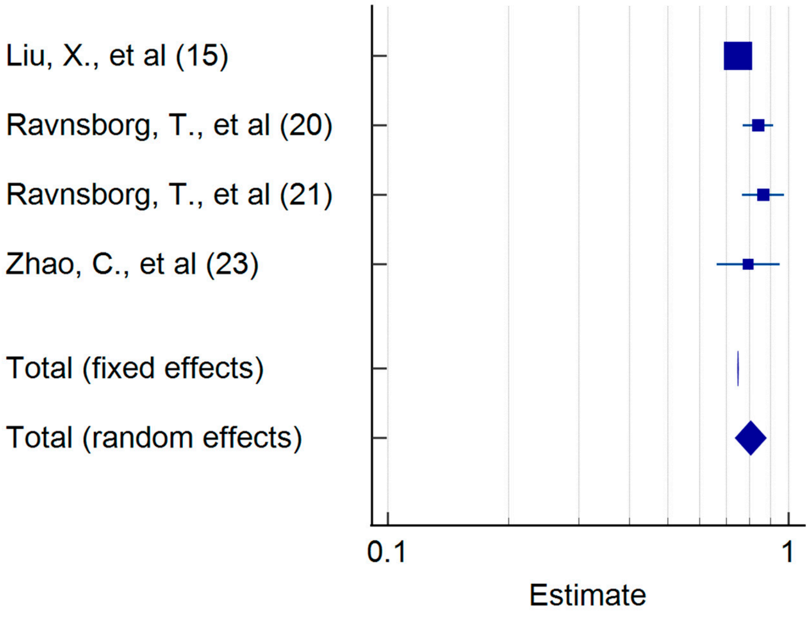

Protein	Study	RoM	95% CI	P	Weight (%)
Sex hormone-binding globulin	Liu, X., et al (15)	0.747	0.746 to 0.749		36.34
	Ravnsborg, T., et al (20)	0.841	0.771 to 0.918		27.10
	Ravnsborg, T., et al (21)	0.866	0.768 to 0.977		21.99
	Zhao, C., et al (23)	0.794	0.661 to 0.952		14.57
	Total (random effects)	0.804	0.735 to 0.880	<0.001	100.00
Heterogeneity	I^2 = 77.39%, P = 0.0041				

Supplementary Figure S23. Forest plot for C-reactive protein. GDM compared to controls.

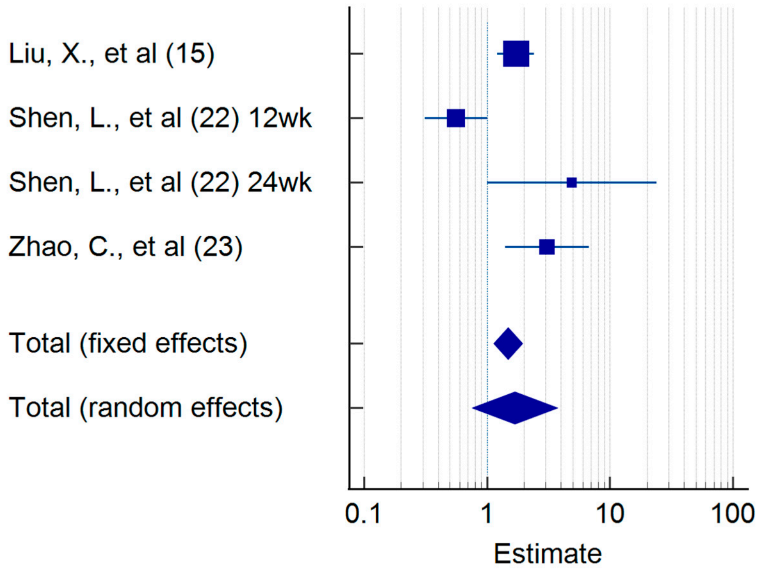

Protein	Study	RoM	95% CI	P	Weight (%)
C-reactive protein	Liu, X., et al (15)	1.714	1.217 to 2.415		31.57
	Shen, L., et al (22) 12wk	0.560	0.314 to 1.000		28.51
	Shen, L., et al (22) 24wk	4.880	1.000 to 23.815		14.52
	Zhao, C., et al (23)	3.070	1.403 to 6.719		25.40
	Total (random effects)	1.682	0.754 to 3.751	0.204	100.00
Heterogeneity	I^2 = 82.36%, P = 0.0007				

Supplementary Figure S24. Forest plot for Glyceraldehyde-3-phosphate dehydrogenase. GDM compared to controls.

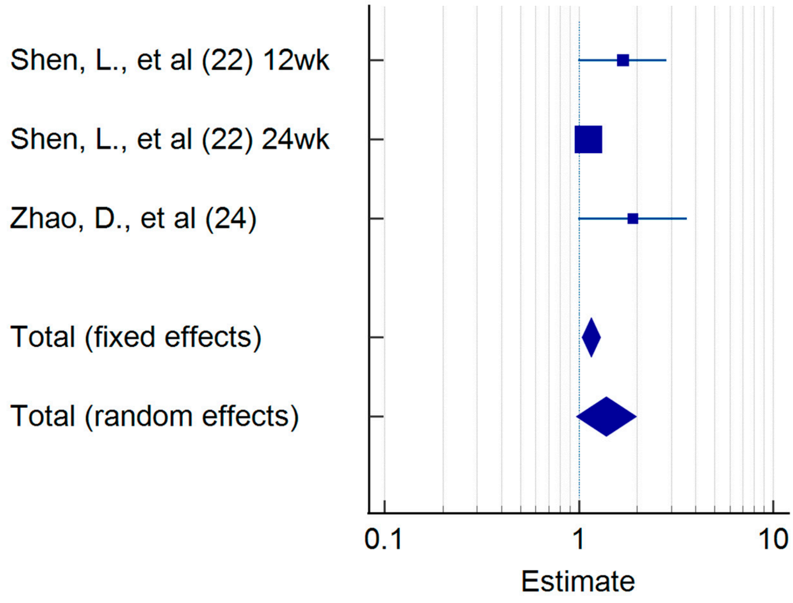

Protein	Study	RoM	95% CI	P	Weight (%)
Glyceraldehyde-3-phosphate dehydrogenase	Shen, L., et al (22) 12wk	1.680	1.000 to 2.822		25.91
	Shen, L., et al (22) 24wk	1.120	1.000 to 1.254		53.99
	Zhao, D., et al (24)	1.900	1.000 to 3.610		20.09
	Total (random effects)	1.383	0.968 to 1.978	0.075	100.00
Heterogeneity	I^2 = 56.48%, P = 0.1005				

Supplementary Figure S25. Forest plot for Insulin-like growth factor-binding protein 5. GDM compared to controls.

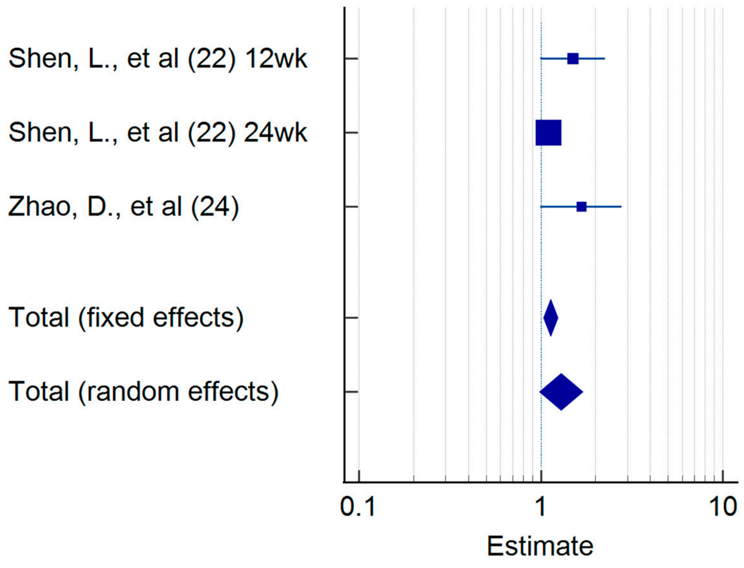

Protein	Study	RoM	95% CI	P	Weight (%)
Insulin-like growth factor-binding protein 5	Shen, L., et al (22) 12wk	1.500	1.000 to 2.250		25.86
	Shen, L., et al (22) 24wk	1.100	1.000 to 1.210		54.76
	Zhao, D., et al (24)	1.670	1.000 to 2.789		19.38
	Total (random effects)	1.292	0.981 to 1.703	0.069	100.00
Heterogeneity	I^2 = 54.62%, P = 0.1104				

Supplementary Figure S26. Forest plot for Alpha-1-antitrypsin**.** GDM compared to controls.

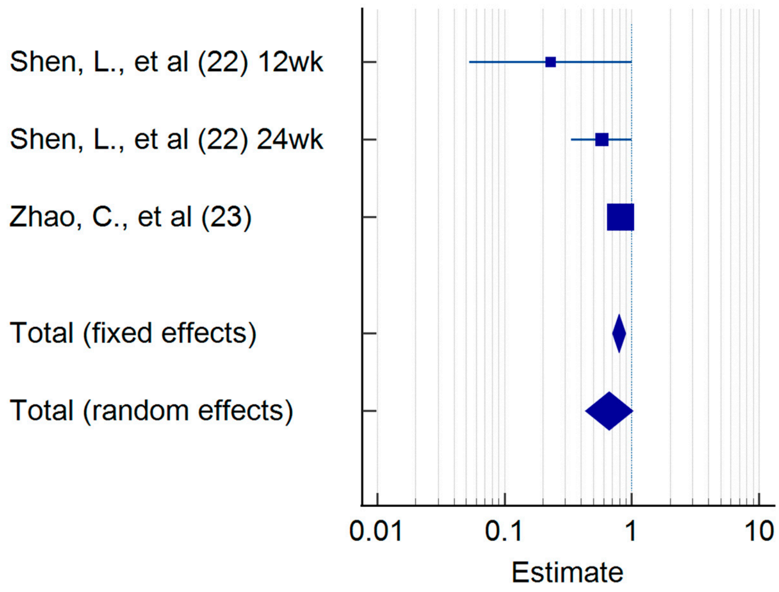

Protein	Study	RoM	95% CI	P	Weight (%)
Alpha-1-antitripsin	Shen, L., et al (22) 12wk	0.230	0.0529 to 1.000		7.65
	Shen, L., et al (22) 24wk	0.580	0.336 to 1.000		31.75
	Zhao, C., et al (23)	0.813	0.719 to 0.920		60.60
	Total (random effects)	0.663	0.430 to 1.023	0.063	100.00
Heterogeneity	I^2 = 51.79%, P = 0.1257				

Supplementary Figure S27. Forest plot for Annexin A4. GDM compared to controls.

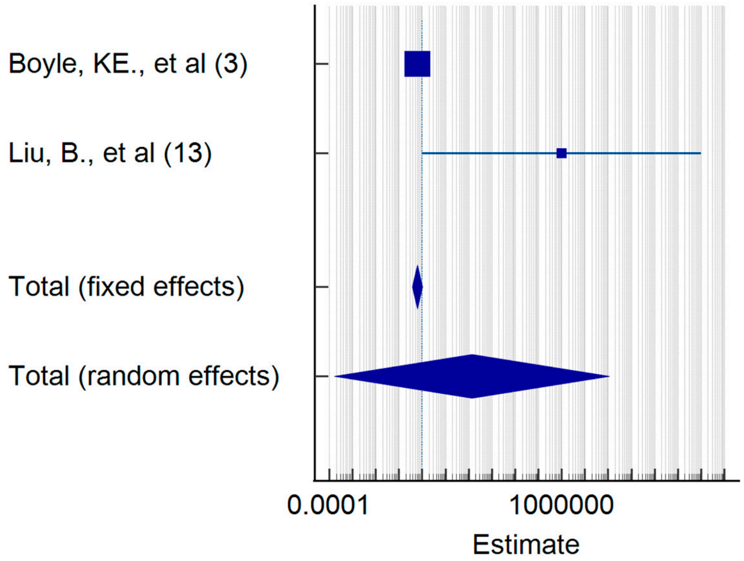

Protein	Study	RoM	95% CI		P	Weight (%)
Annexin A4	Boyle, KE., et al (3)	0.606	0.367 1.000	to		62.11
	Liu, B., et al (13)	10E07	1.000 1.000E07	to		37.89
	Total (random effects)	137.598	0.000169 1.123E08	to	0.478	100.00
Heterogeneity	I^2 = 75.73%, P = 0.0424					

Supplementary Figure S28. Forest plot for Apolipoprotein M. GDM compared to controls.

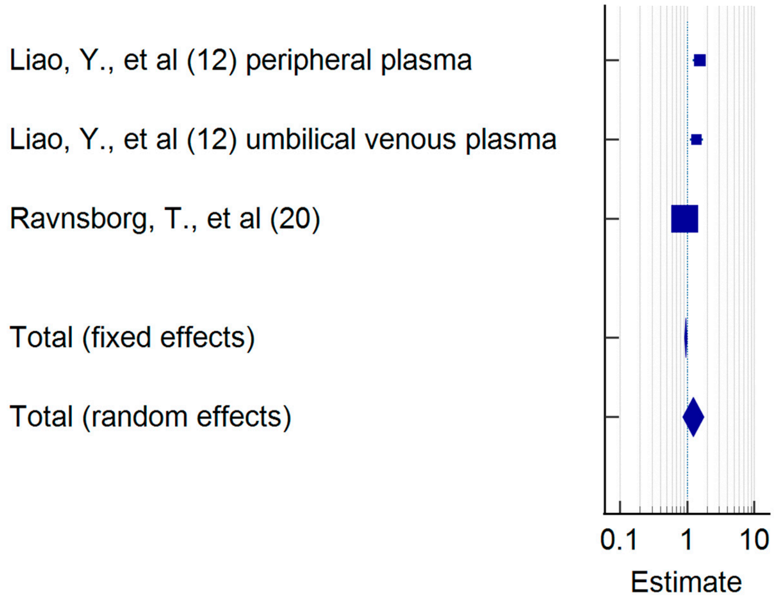

Protein	Study	RoM	95% CI	P	Weight (%)
Apolipoprotein M	Liao, Y., et al (12) peripheral plasma	1.530	1.235 to 1.896		32.17
	Liao, Y., et al (12) umbilical venous plasma	1.380	1.102 to 1.728		31.81
	Ravnsborg, T., et al (20)	0.926	0.887 to 0.966		36.01
	Total (random effects)	1.235	0.858 to 1.779	0.256	100.00
Heterogeneity	I2 = 93.53%, P < 0.0001				

Supplementary Figure S29. Forest plot for Coagulation factor V. GDM compared to controls.

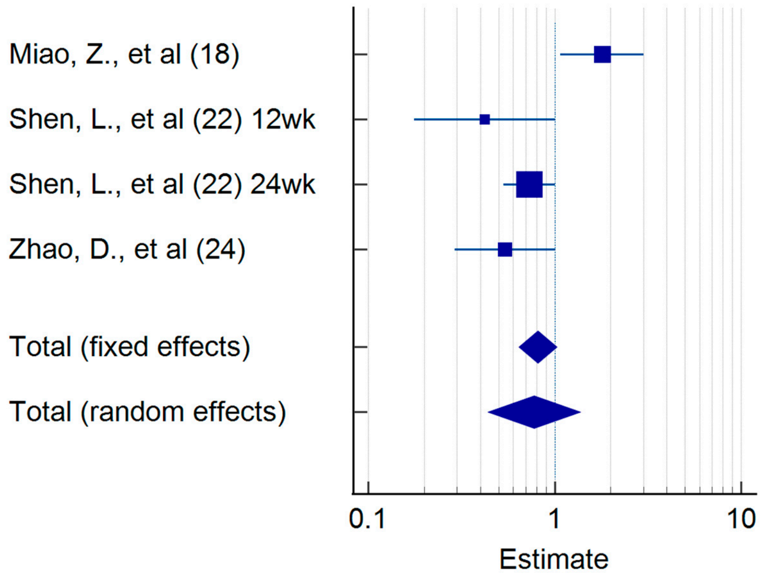

Protein	Study	RoM	95% CI	P	Weight (%)
Coagulation factor V	Miao, Z., et al (18)	1.797	1.077 to 2.998		26.45
	Shen, L., et al (22) 12wk	0.420	0.176 to 1.000		18.94
	Shen, L., et al (22) 24wk	0.730	0.533 to 1.000		30.46
	Zhao, D., et al (24)	0.540	0.292 to 1.000		24.15
	Total (random effects)	0.776	0.438 to 1.374	0.384	100.00
Heterogeneity	I^2 = 77.93 %, P = 0.0035				

Supplementary Figure S30. Forest plot for Complement factor B. GDM compared to controls.

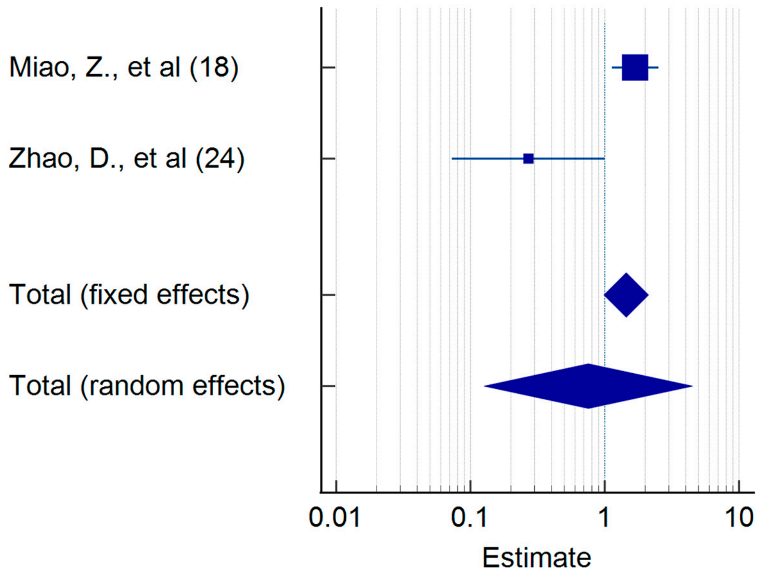

Protein	Study	RoM	95% CI	P	Weight (%)
Complement factor B	Miao, Z., et al (18)	1.692	1.133 to 2.528		56.00
	Zhao, D., et al (24)	0.270	0.0729 to 1.000		44.00
	Total (random effects)	0.755	0.127 to 4.500	0.757	100.00
Heterogeneity	I^2 = 85.50%, P = 0.0086				

Supplementary Figure S31. Forest plot for Fibrinogen alpha chain. GDM compared to controls.

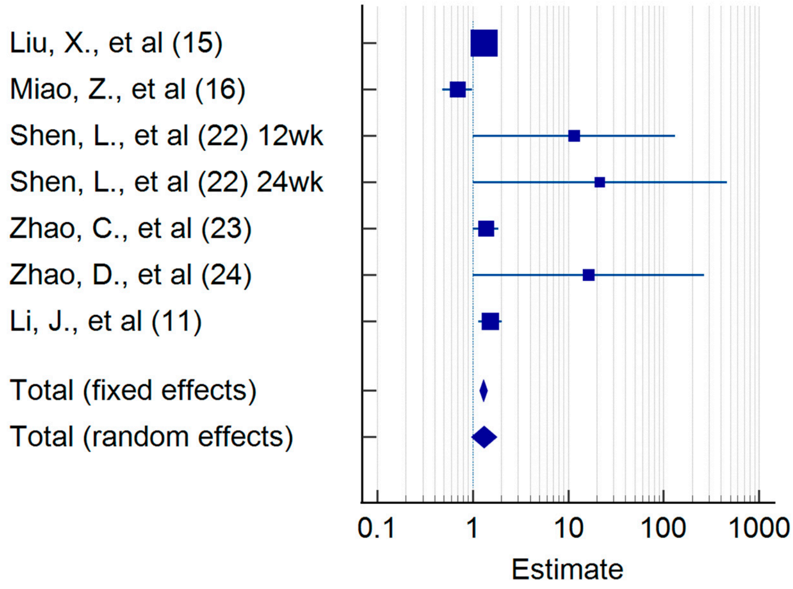

Protein	Study	RoM	95% CI	P	Weight (%)
Fibrinogen alpha chain	Liu, X., et al (15)	1.316	1.181 to 1.465		28.41
	Miao, Z., et al (16)	0.690	0.484 to 0.983		21.29
	Shen, L., et al (22) 12wk	11.550	1.000 to 133.408		1.53
	Shen, L., et al (22) 24wk	21.480	1.000 to 461.416		0.99
	Zhao, C., et al (23)	1.370	1.011 to 1.856		22.96
	Zhao, D., et al (24)	16.330	1.000 to 266.683		1.19
	Li, J., et al (11)	1.522	1.147 to 2.020		23.64
	Total (random effects)	1.312	0.962 to 1.790	0.087	100.00
Heterogeneity	I^2 = 74.01%, P = 0.0008				

Supplementary Figure S32. Forest plot for Fibrinogen beta chain. GDM compared to controls.

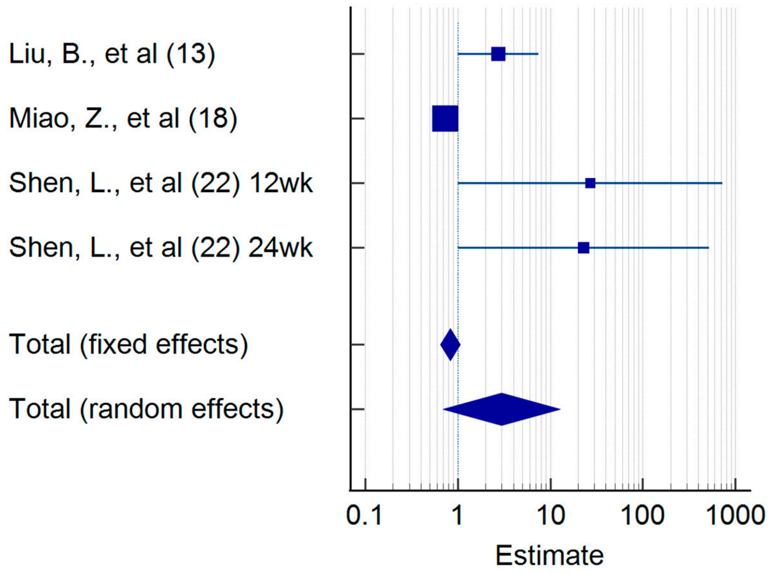

Protein	Study	RoM	95% CI	P	Weight (%)
Fibrinogen beta chain	Liu, B., et al (13)	2.733	1.000 to 7.469		33.49
	Miao, Z., et al (18)	0.734	0.568 to 0.949		39.33
	Shen, L., et al (22) 12wk	27.040	1.000 to 731.206		13.13
	Shen, L., et al (22) 24wk	22.890	1.000 to 523.982		14.06
	Total (random effects)	2.969	0.690 to 12.774	0.144	100.00
Heterogeneity	I^2 = 79.83%, P = 0.0019				

Supplementary Figure S33. Forest plot for Fibrinogen gamma chain. GDM compared to controls.

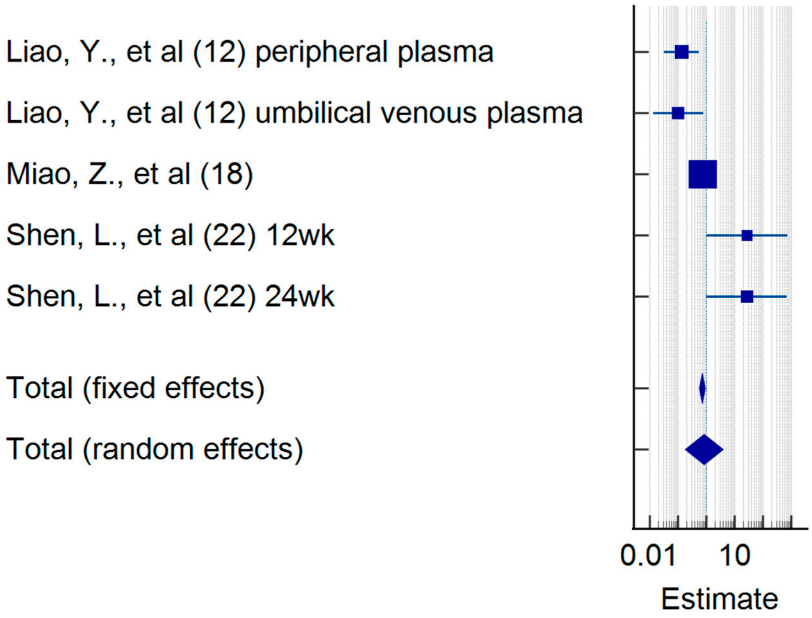

Protein	Study	RoM	95% CI	P	Weight (%)
Fibrinogen gamma chain	Liao, Y., et al (12) peripheral plasma	0.136	0.0327 to 0.565		24.19
	Liao, Y., et al (12) umbilical venous plasma	0.102	0.0132 to 0.779		19.96
	Miao, Z., et al (18)	0.742	0.579 to 0.952		30.08
	Shen, L., et al (22) 12wk	27.160	1.000 to 737.710		12.84
	Shen, L., et al (22) 24wk	26.630	1.000 to 709.200		12.93
	Total (random effects)	0.835	0.176 to 3.964	0.820	100.00
Heterogeneity	I^2 = 77.97%, P = 0.0011				

Supplementary Figure S34. Forest plot for IGL@ protein. GDM compared to controls.

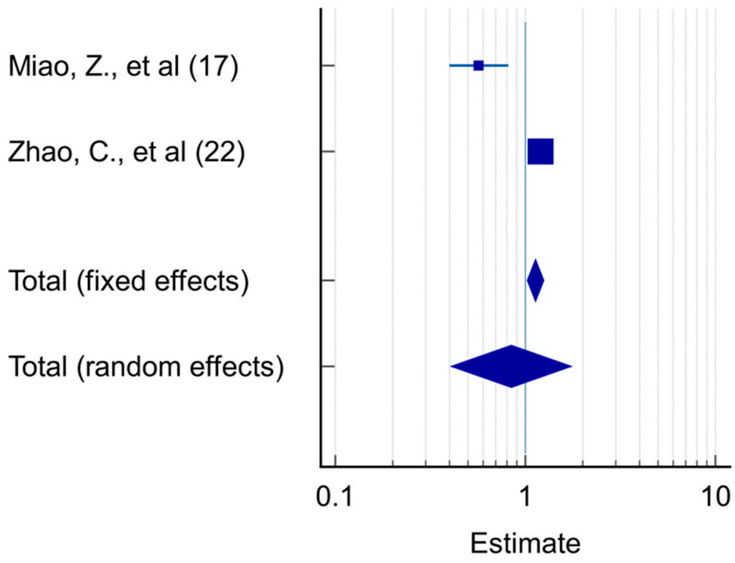

Protein	Study	RoM	95% CI	P	Weight (%)
IGL@ protein	Miao, Z., et al (17)	0.569	0.397 to 0.814		47.26
	Zhao, C., et al (22)	1.200	1.080 to 1.333		52.74
	Total (random effects)	0.843	0.406 to 1.751	0.647	100.00
Heterogeneity	I^2 = 93.48%, P = 0.0001				

Supplementary Figure S35. Forest plot for Mannan-binding lectin serine protease 2. GDM compared to controls.

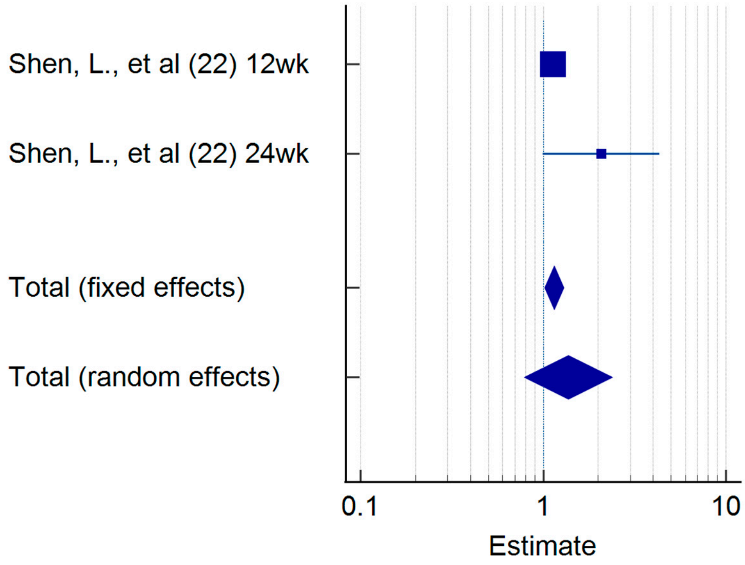

Protein	Study	RoM	95% CI	P	Weight (%)
Mannan-binding lectin serine protease 2	Shen, L., et al (22) 12wk	1.130	1.000 to 1.277		68.23
	Shen, L., et al (22) 24wk	2.080	1.000 to 4.326		31.77
	Total (random effects)	1.372	0.786 to 2.394	0.266	100.00
Heterogeneity	I^2 = 61.45%, P = 0.1073				

Supplementary Figure S36. Forest plot for Plasma protease C1 inhibitor. GDM compared to controls.

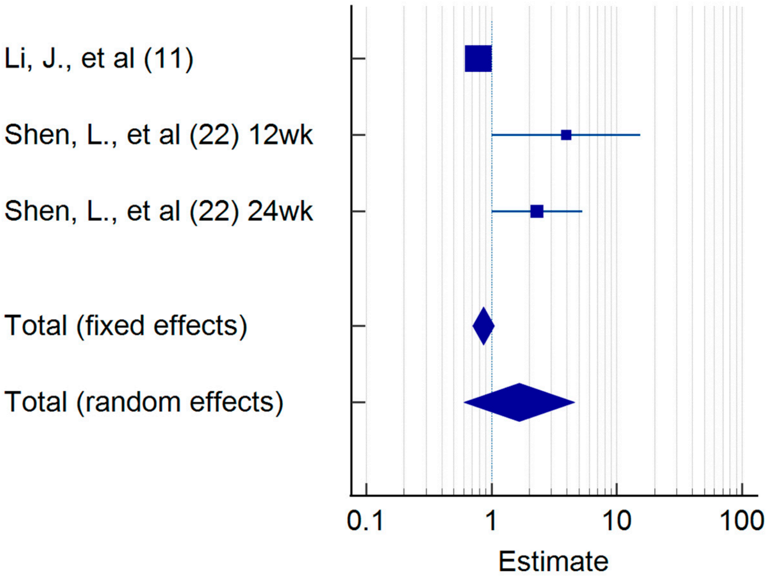

Protein	Study	RoM	95% CI	P	Weight (%)
Plasma protease C1 inhibitor	Li, J., et al (11)	0.784	0.638 to 0.964		42.38
	Shen, L., et al (22) 12wk	3.940	1.000 to 15.524		24.22
	Shen, L., et al (22) 24wk	2.310	1.000 to 5.336		33.40
	Total (random effects)	1.663	0.600 to 4.608	0.328	100.00
Heterogeneity	I^2 = 81.57%, P = 0.0044				

Supplementary Figure S37. Forest plot for Pregnancy zone protein. GDM compared to controls.

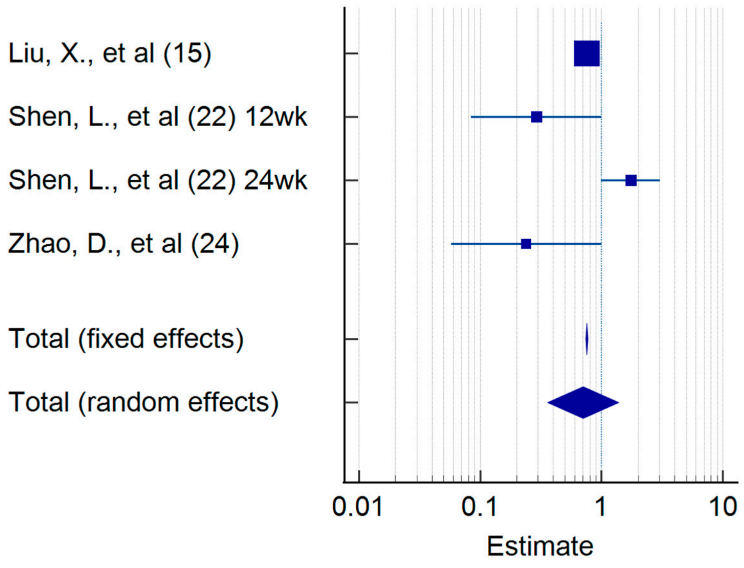

Protein	Study	RoM	95% CI	P	Weight (%)
Pregnancy zone protein	Liu, X., et al (15)	0.755	0.740 to 0.770		38.41
	Shen, L., et al (22) 12wk	0.290	0.0841 to 1.000		16.83
	Shen, L., et al (22) 24wk	1.740	1.000 to 3.028		30.57
	Zhao, D., et al (24)	0.240	0.0576 to 1.000		14.20
	Total (random effects)	0.705	0.358 to 1.388	0.312	100.00
Heterogeneity	I^2 = 77.78%, P = 0.0037				

Supplementary Figure S38. Forest plot for Prothrombin. GDM compared to controls.

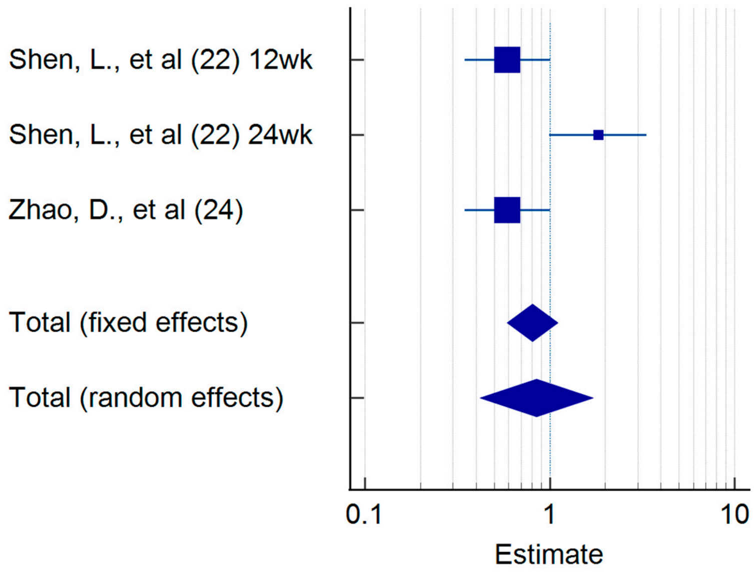

Protein	Study	RoM	95% CI	P	Weight (%)
Prothrombin	Shen, L., et al (22) 12wk	0.590	0.348 to 1.000		33.97
	Shen, L., et al (22) 24wk	1.830	1.000 to 3.349		32.06
	Zhao, D., et al (24)	0.590	0.348 to 1.000		33.97
	Total (random effects)	0.848	0.419 to 1.715	0.647	100.00
Heterogeneity	I^2 = 79.50%, P = 0.0076				

Supplementary Figure S39. Forest plot for Retinol-binding protein 4. GDM compared to controls.

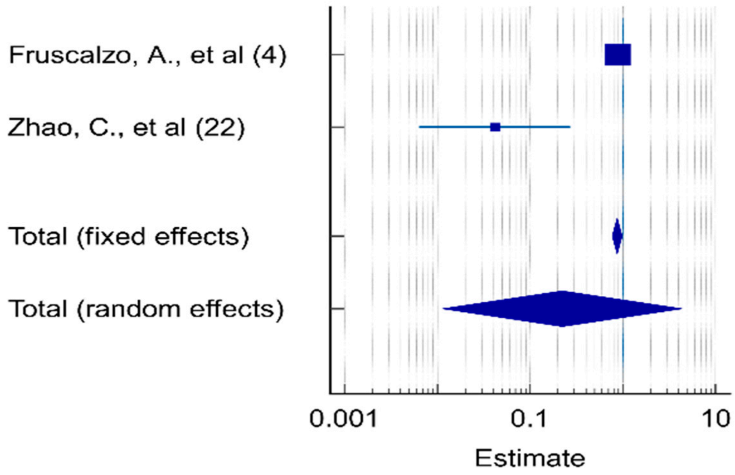

Protein	Study	RoM	95% CI	P	Weight (%)
Retinol-binding protein 4	Fruscalzo, A., et al (4)	0.884	0.781 to 1.001		54.89
	Zhao, C., et al (22)	0.0417	0.00638 to 0.272		45.11
	Total (random effects)	0.223	0.0113 to 4.385	0.323	100.00
Heterogeneity	I^2 = 90.14%, P = 0.0015				

Supplementary Figure S40. Forest plot for Serum paraoxonase/arylesterase 1. GDM compared to controls.

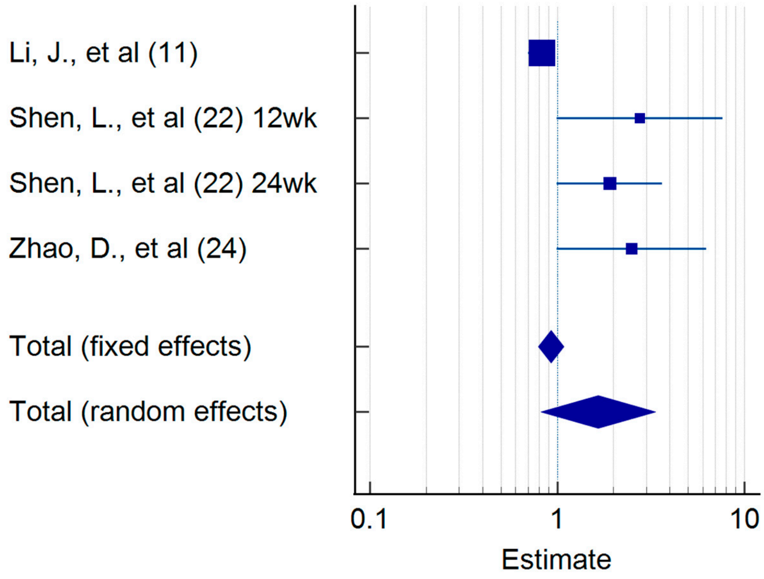

Protein	Study	RoM	95% CI			P	Weight (%)
Serum paraoxonase/arylesterase 1	Li, J., et al (11)	0.830	0.702	to	0.981		32.84
	Shen, L., et al (22) 12wk	2.770	1.000	to	7.673		19.69
	Shen, L., et al (22) 24wk	1.910	1.000	to	3.648		26.10
	Zhao, D., et al (24)	2.500	1.000	to	6.250		21.37
	Total (random effects)	1.655	0.818	to	3.349	0.161	100.00
Heterogeneity	I^2 = 80.55%, P = 0.0015						
