# Supplementary material for: Proteomics Studies in Gestational Diabetes Mellitus: A Systematic Review and Meta-Analysis
Source: J Clin Med. 2022 May 12;11(10):2737. doi: 10.3390/jcm11102737 (PMC9143836; doi:10.3390/jcm11102737)
Supplement: Supplementary file 1 [file jcm-11-02737-s001.zip › jcm-1695841-SI/Supplementary Table 4.pdf]

Supplementary Table S4. Replicability of 262 CB across 23 independent cohorts

1 cohort (213 CB)

Number	Candidate biomarker (CB)	UniProt ID	Study (First name, year)
1	14-3-3 protein zeta/delta	P63104	Liu, B. 2018
2	26S proteasome non-ATPase regulatory subunit 14	O00487	Liu, B. 2018
3	78 kDa glucose-regulated protein	B5X397	Ravnsborg, T. 2019
4	ADAMTS-like protein 2	Q86TH1	Ravnsborg, T. 2019
5	Adhesion G-protein coupled receptor G6	Q86SQ4	Ravnsborg, T. 2019
6	Adipocyte plasma membrane –associated protein	Q9HDC9	Zhao, D. 2017
7	Adiponectin	Q15848	*Ravnsborg, T. 2016 and 2019
8	Afamin	P43652	Ravnsborg, T. 2019
9	Agrin (AGRN)	O00468	Kopylov, A.T. 2020
10	Alpha 2 type IV collagen preproprotein	P08572	Ramachandrarao, SP. 2016
11	Alpha-1-syntrophin	Q13424	Boyle, KE. 2014
12	Alpha-2-HS-glycoprotein	P02765	Liao, Y. 2018
13	Alpha-N-acetylglucosaminidase	P54802	Liu, X. 2020
14	Amine oxidase [flavin-containing] A	P21397	Ma, Y. 2016
15	Aminopeptidase N	P15144	Miao, Z. 2016
16	Annexin A2	P07355	Liu, B. 2018
17	Annexin A5	P08758	Liu, B. 2018
18	Apolipoprotein A-II	P02652	Kim, SM. 2012
19	Apolipoprotein C-II	P02655	Ai, T. 2015
20	Apolipoprotein D	P05090	Ravnsborg, T. 2016
21	ATP-dependent RNA helicase DDX55	Q8NHQ9	Li, J. 2021
22	ATP-dependent translocase ABCB1	P08183	Kopylov, A.T. 2020
23	Basement membrane-specific heparan sulfate proteoglycan core protein	P98160	Mavreli, D. 2020

24	Beta-ala-his dipeptidase	Q96KN2	Mavreli, D. 2020
25	BMP/retinoic acid-inducible neural-specific protein 1	O60477	Miao, Z. 2016
26	C14orf159 isoform 2 of UPF0317 protein C14orf1	NA	Ma, Y. 2016
27	C20orf3 isoform 1 of adipocyte plasma membrane associated protein	NA	Ma, Y. 2016
28	Calcium/calmodulin - dependent protein kinase II beta	Q13554	Jayabalan, N. 2019
29	Calcium/calmodulin-dependent protein kinase kinase 2	Q96RR4	Miao, Z. 2016
30	Calmodulin-dependent calcineurin A β	NA	Boyle, KE. 2014
31	Calpastatin	P20810	Miao, Z. 2016
32	Carboxymethylenebutenolidase homolog	Q96DG6	Boyle, KE. 2014
33	Carboxypeptidase N catalytic chain	P15169	Ravnsborg, T. 2019
34	Carboxypeptidase N subunit 2	P22792	Ravnsborg, T. 2019
35	carboxypeptidase N, polypeptide 2	NA	Ramachandrarao, SP. 2016
36	Cathelicidin antimicrobial peptide	P49913	Zhao, C. 2015
37	Cathepsin Z	Q9UBR2	Ravnsborg, T. 2019
38	Cation-independent mannose-6-phosphate receptor	P11717	Kopylov, A.T. 2020
39	CD59	P13987	Guo, Y. 2018
40	cDNA FLJ14473 fis	Q96K68	Zhao, C. 2015
41	cDNA FLJ50585, highly similar to T-complex protein 1 subunit beta	B7Z4R3	Zhao, C. 2015
42	cDNA FLJ55606	B7Z8Q2	Zhao, C. 2015
43	cDNA FLJ58413	B4E3S6	Zhao, C. 2015
44	Ceruloplasmin	P00450	Shen, L. 2019
45	Cholesteryl ester transfer protein	P11597	Liao, Y. 2018
46	chromatin modifying protein 2A	A0A024R4S0	Ramachandrarao, SP. 2016
47	Citrate lyase subunit beta-like protein, mitochondrial	Q8N0X4	Liu, X. 2020
48	Clusterin	P10909	Ai, T. 2015

49	Clusterin alpha chain	NA	Ravnsborg, T. 2016
50	Coagulation factor XIII B chain	P05160	Zhao, C. 2015
51	Cofilin-1	P23528	Liu, X. 2020
52	Coiled-coil domain-containing protein R3HCC1L	Q7Z5L2	Miao, Z. 2016
53	Complement C1q subcomponent subunit B	P02746	Miao, Z. 2016
54	Complement C1q subcomponent subunit C	P02747	Miao, Z. 2016
55	Complement C1r subcomponent	P00736	Shen, L. 2019
56	Complement C4-B	P0C0L5	Liao, Y. 2018
57	Complement C5	P01031	Shen, L. 2019
58	Connective tissue growth factor	Q5M8T4	Ravnsborg, T. 2019
59	Crk-like protein	P46109	Miao, Z. 2016
60	Cystatin-C	P01034	Li, J. 2021
61	Cystatin-M	Q15828	Ravnsborg, T. 2019
62	Desmoglein-2	Q14126	Ravnsborg, T. 2019
63	Desmoplakin	P15924	Miao, Z. 2016
64	Dihydropyrimidine dehydrogenase [NADP(+)]	Q12882	Miao, Z. 2016
65	Disintegrin and metalloproteinase domain-containing protein 12	O43184	Li, J. 2021
66	DNA polymerase epsilon catalytic subunit A	Q07864	Miao, Z. 2016
67	Dynein assembly factor 1, axonemal	Q8NEP3	Miao, Z. 2016
68	Ectonucleotide pyrophosphatase/phosphodiesterase family member 2	Q13822	Li, J. 2021
69	Ezrin	P15311	Kopylov, A.T. 2020
70	Fatty acid binding protein 4	P15090	Jayabalan, N. 2019
71	Ferritin light chain	P02792	Ravnsborg, T. 2019
72	Ficolin-3	O75636	Zhao, C. 2015
73	Folate receptor 1 precursor	P15328	Ramachandrarao, SP. 2016
74	Full-length cDNA clone CS0DD006YL02 of neuroblastoma of homo sapiens (human)	Q86TT1	Zhao, C. 2015
75	Fumarate hydratase, mitochondrial	P07954	Liu, B. 2018

76	Galectin-3-binding protein	Q08380	Zhao, D. 2017
77	GC-rich sequence DNA-binding factor 2	P16383	Miao, Z. 2016
78	Glycyl-tRNA synthetase	P41250	Liu, B. 2018
79	Golgin subfamily A member 4	Q13439	Miao, Z. 2016
80	Growth hormone receptor	P10912	Liu, X. 2020
81	Guanine nucleotide-binding protein G(I)/G(S)/G(T) subunit beta-2	P62879	Liu, B. 2018
82	Hemoglobin subunit beta	P68871	Shen, L. 2019
83	Hexokinase-3	P52790	Jayabalan, N. 2019
84	Histone cluster 2, H4b	NA	Ramachandrarao, SP. 2016
85	Histone cluster 3, H3	NA	Ramachandrarao, SP. 2016
86	Human serum albumin-cys	NA	Boisvert, MR. 2010
87	Ig gamma-4 chain C region	P01861	Miao, Z. 2016
88	Ig heavy chain V-I region V35	P23083	Liu, X. 2020
89	Ig heavy chain V-II region SESS	P04438	Zhao, C. 2015
90	Ig heavy chain V-II region WAH	P01824	Zhao, C. 2015
91	Ig kappa chain C region	P01834	Miao, Z. 2016
92	Ig kappa chain V-I region DEE	P01597	Zhao, C. 2015
93	Ig kappa chain V-I region Roy	P01608	Zhao, C. 2015
94	Ig kappa chain V-III region CLL	P04207	Zhao, C. 2015
95	Ig kappa chain V-III region HAH	P18135	Miao, Z. 2016
96	Ig kappa chain V-III region HIC	P18136	Miao, Z. 2016
97	Ig kappa chain V-IV region B17	P06314	Miao, Z. 2016
98	Ig lambda chain V-III region LOI	P80748	Liu, X. 2020
99	Ig lambda-2 chain C regions	P0CG05	Miao, Z. 2016
100	IgGFc-binding protein	Q9Y6R7	Ravnsborg, T. 2019
101	IGK@ protein	Q6P5S8	Zhao, C. 2015
102	Immunoglobulin heavy chain	A2KLM6	Zhao, C. 2015
103	Immunoglobulin heavy chain variant	Q9NPP6	Zhao, C. 2015

104	Immunoglobulin heavy variable 2-70D	A0A0C4DH43	Li, J. 2021
105	Immunoglobulin J chain	P01591	Shen, L. 2019
106	Immunoglobulin kappa variable 3-15	P01624	Li, J. 2021
107	Immunoglobulin lambda constant 2	P0DOY2	Li, J. 2021
108	Immunoglobulin lambda constant 3	P0DOY3	Li, J. 2021
109	Immunoglobulin lambda variable 7-43	P04211	Li, J. 2021
110	Insulin-like growth factor-binding protein 2	P18065	Liu, X. 2020
111	Insulin-like growth factor-binding protein complex acid labile subunit	P35858	Shen, L. 2019
112	Inter alpha trypsin inhibitor heavy chain H1	P19827	Zhao, D. 2017
113	Inter alpha trypsin inhibitor heavy chain H4	Q14624	Zhao, D. 2017
114	Inter-alpha (globulin) inhibitor H2, isoform CRA_a	D3DRR6	Zhao, C. 2015
115	Inter-alpha-trypsin inhibitor heavy chain H1	P19827	Shen, L. 2019
116	Interleukin-1 receptor antagonist	P18510	Guo, Y. 2018
117	Isthmin-2	Q6H9L7	Liu, X. 2020
118	Kinesin heavy chain isoform 5C	O60282	Miao, Z. 2016
119	Kininogen-1	P01042	Liao, Y. 2018
120	Lactotransferrin	P02788	Kopylov, A.T. 2020
121	Laminin subunit alpha-4	Q16363	Ma, Y. 2016
122	Leucyl-cystinyl aminopeptidase	Q9UIQ6	Li, J. 2021
123	Lipopolysaccharide-binding protein	P18428	Kopylov, A.T. 2020
124	Lumican	P51884	Shen, L. 2019
125	Macrophage migration inhibitory factor	P14174	Ramachandrarao, SP. 2016
126	Mannan-binding lectin serine protease 1	P48740	Shen, L. 2019
127	Mitochondrial amidoxime-reducing component 1	Q5VT66	Ma, Y. 2016
128	Monocyte differentiation antigen CD14	P08571	Kopylov, A.T. 2020
129	Myeloperoxidase	P05164	Zhao, D. 2017
130	Myosin 11	P35749	Liu, F. 2016

131	Myosin regulatory light polypeptide 9	P24844	Liu, B. 2018
132	Myosin-reactive immunoglobulin heavy chain variable region	Q9UL92 and Q9UL95	Zhao, C. 2015
133	Myosin-reactive immunoglobulin light chain variable region	Q9UL82	Zhao, C. 2015
134	N-acetylglucosamine-1-phosphotransferase subunit gamma	Q9UJJ9	Li, J. 2021
135	NADH dehydrogenase 1 alpha subcomplex subunit 9	Q16795	Ma, Y., et al 2016
136	NADH dehydrogenase flavoprotein 2	P19404	Boyle, KE. 2014
137	NADH dehydrogenase iron-sulfur protein 3	O75489	Boyle, KE. 2014
138	Neutrophil gelatinase-associated lipocalin	P80188	Ravnsborg, T. 2016
139	Perilipin 4	Q96Q06	Jayabalan, N. 2019
140	Phosphatidylcholine-sterol acyltransferase	P04180	Miao, Z. 2016
141	Phosphodiesterase 8A	Q9UMB5	Kopylov, A.T. 2020
142	Plasma alpha-L-fucosidase	Q9BTY2	Li, J. 2021
143	Plasma kallikrein	P03952	Shen, L. 2019
144	Plasma serine protease inhibitor	P05154	Kopylov, A.T. 2020
145	Platelet glycoprotein 4	P16671	Boyle, KE. 2014
146	Platelet glycoprotein Ib alpha chain	P07359	Ravnsborg, T. 2019
147	Platelet glycoprotein V	O08770	Ravnsborg, T. 2019
148	Poliovirus receptor	P15151	Liu, X. 2020
149	Polyubiquitin-B	P0CG47	Li, J. 2021
150	Polyubiquitin-C	P0CG48	Li, J. 2021
151	Pregnancy-specific beta-1-glycoprotein 11	Q9UQ72	Li, J. 2021
152	Pregnancy-specific beta-1-glycoprotein 2	P11465	Liu, X., 2020
153	Pregnancy-specific beta-1-glycoprotein 4	Q6P520	Zhao, C. 2015
154	Pregnancy-specific beta-1-glycoprotein 5	Q15238	Li, J. 2021
155	Prenylcysteine oxidase 1	Q9UHG3	Mavreli, D. 2020
156	Programmed cell death 6-interacting protein	Q8WUM4	Liu, X. 2020
157	Prostasin preproprotein	A0A5F4WBI9	Ramachandrarao, SP. 2016
158	Proteasome subunit alpha type-1	P25786	Boyle, KE. 2014

159	Protein disulfide-isomerase	P07237	Zhao, D. 2017
160	Protein Z-dependent protease inhibitor	Q9UK55	Shen, L. 2019
161	Putative heat shock protein HSP 90-beta 4	Q58FF6	Miao, Z. 2016
162	Putative pregnancy-specific beta-1-glycoprotein 7	Q13046	Ravnsborg, T. 2019
163	Putative uncharacterized protein DKFZp686N02209	Q7Z351	Zhao, C. 2015
164	Putative uncharacterized protein DKFZp686O01196	Q6N094	Zhao, C. 2015
165	Ras-related protein Rap-1A	P62834	Liu, B. 2018
166	Ras-related protein R-Ras2	P62070	Kopylov, A.T. 2020
167	Receptor-type tyrosine-protein phosphatase S	Q13332	Ravnsborg, T. 2019
168	Rheumatoid factor RF-IP12	A2J1M8	Zhao, C. 2015
169	Rho guanine nucleotide exchange factor 11	O15085	Miao, Z. 2016
170	S100 calcium-binding protein A9	P06702	Ramachandrarao, SP. 2016
171	Selenoprotein P, plasma, 1	A0A024R054	Kopylov, A.T. 2020
172	Serine protease inhibitor Kazal-type 5	Q9NQ38	Ravnsborg, T. 2019
173	Serotransferrin (transferrin) (beta-1 metal-binding globulin)	P02787	Zhao, C. 2015
174	Serum deprivation response protein	A0A1V4K6T9	Liu, F. 2016
175	Serum paraoxonase/lactonase 3	Q15166	Liu, X. 2020
176	Solute carrier family 3 (activators of dibasic and neutral amino acid transport), member 2 isoform c	A0A024R599	Ramachandrarao, SP. 2016
177	Spectrin alpha chain, erythrocytic 1	P02549	Jayabalan, N. 2019
178	Stomatin isoform a	P27105	Ramachandrarao, SP. 2016
179	Sulfhydryl oxidase 1	O00391	Kopylov, A.T. 2020
180	Superoxide dismutase [Cu-Zn]	P00441	Kopylov, A.T. 2020
181	Suprabasin	Q6UWP8	Ravnsborg, T. 2019
182	Syntenin isoform 1	NA	Ramachandrarao, SP. 2016
183	Syntenin isoform 3	NA	Ramachandrarao, SP. 2016
184	Tenascin-X	P22105	Kopylov, A.T. 2020
185	Thioredoxin	P10599	Kopylov, A.T. 2020
186	Thrombospondin-4	P49744	Mavreli, D. 2020

187	Thymosin beta-4	P62328	Liu, F. 2016
188	Tissue alpha-L-fucosidase	P04066	
189	Transgelin-2	P37802	Zhao, C. 2015
190	Transthyretin glycine	NA	Fruscalzo, A. 2015
191	Trypsin-3	P35030	Liu, X. 2020
192	Trypsin-3	P35030	Liu, X. 2020
193	Tryptophanyl-tRNA synthetase, cytoplasmic	B2L5L1	Liu, B. 2018
194	Tryptophanyl-tRNA synthetase, cytoplasmic	B2L5L1	Liu, B. 2018
195	Tubulin beta chain	P07437	Kopylov, A.T. 2020
196	Tyrosine 3/tryptophan 5 -monooxygenase activation protein, beta polypeptide	Q59EQ2	Ramachandrarao, SP. 2016
197	Ubiquitin-40S ribosomal protein S27a	P62979	Li, J.2021
198	Ubiquitin-60S ribosomal protein L40	P62987	Li, J.2021
199	Uncharacterized protein	Q8NEJ1	Zhao, C. 2015
200	Uncharacterized protein C18orf63	Q68DL7	Li, J.2021
201	Uncharacterized protein C2orf80	H7C2Y3	Miao, Z. 2016
202	V1-3 protein	Q5NV84	Zhao, C. 2015
203	V2-17 protein	Q5NV90	Zhao, C. 2015
204	Vitamin D-binding protein	P02774	Miao, Z. 2016
205	Vitamin K-dependent protein C	P04070	Zhao, D. 2017
206	Vitamin K-dependent protein S	P07225	Shen, L. 2019
207	Vitronectin	P04004	Ravnsborg, T. 2019
208	Von Willebrand factor	P04275	Shen, L. 2019
209	Xaa-Pro dipeptidase	P12955	Liu, X. 2020
210	Zinc finger protein basonuclin-2	Q6ZN30	Liu, X. 2020
211	Zinc finger, AN1-type domain 2B, isoform CRA_c	B3KQB0	Miao, Z. 2016
212	Zinc-alpha-2-glycoprotein	P25311	Li, J. 2021
213	ZNF207 zinc finger protein 207 isoform c	F7BQH6	Ma, Y. 2016

2 cohorts (33 CB)

Number	Candidate biomarker (CB)	UniProt ID	Study (First name, year)
1	Alpha-1-antitrypsin	P01009	Shen, L. 2019<br>Zhao, C. 2015
2	Alpha-2-macroglobulin	P01023	Ravnsborg, T. 2016<br>Li, J. 2021
3	Annexin A4	P09525	Boyle, KE. 2014<br>Liu, B. 2018
4	Antithrombin-III	P01008	Ravnsborg, T. 2019<br>Zhao, D. 2017
5	Apolipoprotein A-IV	P06727	Hajduk, J. 2015<br>Li, J. 2021
6	Apolipoprotein A-V	Q6Q788	Shen, L. 2019<br>Zhao, D. 2017
7	Apolipoprotein L1	O14791	Ravnsborg, T. 2016<br>Li, J. 2021
8	Apolipoprotein M	O95445	Liao, Y. 2018<br>Ravnsborg, T. 2016
9	C4b-binding protein alpha chain	P04003	Shen, L. 2019<br>Zhao, D. 2017
10	Coagulation factor IX	P00740	Shen, L. 2019<br>Zhao, D. 2017
11	Coagulation factor X	P00742	Shen, L. 2019<br>Zhao, D. 2017
12	Coagulation factor XII	P00748	Shen, L. 2019<br>Zhao, D. 2017
13	Complement C1s subcomponent	P09871	Shen, L. 2019<br>Zhao, D. 2017
14	Complement component C6	P13671	Shen, L. 2019<br>Zhao, D. 2017
15	Complement component C7	P10643	Shen, L. 2019<br>Zhao, D. 2017
16	Complement component C8 beta chain	P07358	Shen, L. 2019<br>Zhao, D. 2017
17	Complement component C8 gamma chain	P07360	Shen, L. 2019<br>Zhao, D. 2017
18	Complement factor B	B4E1Z4	Miao, Z. 2016<br>Zhao, D. 2017
19	Complement factor H	P08603	Shen, L. 2019<br>Zhao, D. 2017
20	Endoplasmin	P14625	Shen, L. 2019<br>Zhao, D. 2017
21	Extracellular matrix protein 1	Q16610	Mavreli, D. 2020<br>Li, J. 2021

22	Glyceraldehyde-3-phosphate dehydrogenase	P04406	Shen, L. 2019<br>Zhao, D. 2017
23	Haptoglobin	P00738	Ramachandrarao, SP. 2016<br>Ravnsborg, T. 2016
24	IGL@ protein	Q6PJR7	Miao, Z. 2016<br>Zhao, C. 2015
25	Insulin-like growth factor-binding protein 5	P24593	Shen, L. 2019<br>Zhao, D. 2017
26	Mannan-binding lectin serine protease 2	O00187	Kopylov, A.T. 2020<br>Shen, L. 2019
27	Pappalysin-1	Q13219	Jayabalan, N. 2019<br>Zhao, C. 2015
28	Phospholipid transfer protein	P55058	Miao, Z. 2016<br>Ravnsborg, T. 2019
29	Plasma protease C1 inhibitor	P05155	Shen, L. 2019<br>Li, J. 2021
30	Prothrombin	P00734	Shen, L. 2019<br>Zhao, D. 2017
31	Serum amyloid A-2 protein	P0DJI9	Kopylov, A.T. 2020<br>Liu, X. 2020
32	Serum amyloid P-component	P02743	Liu, X. 2020<br>Ravnsborg, T. 2019
33	Transthyretin	P02766	Fruscalzo, A. 2015<br>Ilyas, S. 2020

3 cohorts (15 CB)

**Number**	**Candidate biomarker (CB)**	**UniProt ID**	**Study (First name, year)**
1	Apolipoprotein C-III	P02656	Kim, SM. 2012<br>Shen, L. 2019<br>Li, J. 2021
2	Apolipoprotein E	P02649	Shen, L. 2019<br>Zhao, D. 2017<br>Li, J. 2021
3	Coagulation factor V	P12259	Miao, Z. 2016<br>Shen, L. 2019<br>Zhao, D. 2017
4	Complement component C9	P02748	Miao, Z. 2016<br>Shen, L. 2019<br>Zhao, D. 2017
5	C-reactive protein	P02741	Liu, X. 2020<br>Shen, L. 2019<br>Zhao, C. 2015

6	Fibrinogen beta chain	P02675	Liu, B. 2018<br>Miao, Z. 2016<br>Shen, L. 2019
7	Fibrinogen gamma chain	P02679	Liao, Y. 2018<br>Miao, Z. 2016<br>Shen, L. 2019
8	Gelsolin	P06396	Shen, L. 2019<br>Zhao, D. 2017<br>Li, J. 2021
9	Ig mu chain C region (Immunoglobulin heavy constant mu)	P01871	Liu, X. 2020<br>Shen, L. 2019<br>Zhao, D. 2017
10	Pregnancy zone protein	P20742	Liu, X. 2020<br>Shen, L. 2019<br>Zhao, D. 2017
11	Proteoglycan 4	Q92954	Liu, X. 2020<br>Shen, L. 2019<br>Zhao, D. 2017
12	Retinol-binding protein 4	P02753	Fruscalzo, A. 2015<br>Ilyas, S. 2020<br>Zhao, C. 2015
13	Secreted phosphoprotein 24	Q13103	Liu, X. 2020<br>Ravnsborg, T. 2019<br>Shen, L. 2019
14	Serum paraoxonase/arylesterase 1	P27169	Shen, L. 2019<br>Zhao, D. 2017<br>Li, J. 2021
15	Sex hormone-binding globulin	P04278	Liu, X. 2020<br>*Ravnsborg, T. 2016 and 2019<br>Zhao, C. 2015

10 cohorts (1 CB)

**Number**	**Candidate biomarker (CB)**	**UniProt ID**	**Study (First name, year)**
1	Fibrinogen alpha chain	P02671	Ai, T. 2015<br>Hajduk, J. 2015<br>Liu, B. 2018<br>Liu, F. 2016<br>Liu, X. 2020<br>Miao, Z. 2016<br>Shen, L. 2019<br>Zhao, C. 2015<br>Zhao, D. 2017<br>Li, J. 2021

Note: NA (Data not available)
