# Supplementary material for: Proteomics Studies in Gestational Diabetes Mellitus: A Systematic Review and Meta-Analysis
Source: J Clin Med. 2022 May 12;11(10):2737. doi: 10.3390/jcm11102737 (PMC9143836; doi:10.3390/jcm11102737)
Supplement: Supplementary file 1 [file jcm-11-02737-s001.zip › jcm-1695841-SI/Supplementary Tables 1 2 3 5 6 7 8 9.pdf]

Supplementary Table S1. Summary of included studies (n = 24) on differential protein expressions in GDM and controls

Author (Ref)	Year	Country	Continent	Age Analysis	Trimester (Analysis)	GDM Diagnosis Criteria	Glucose Dose	Age at GDM diagnosis	Trimester (GDM diagnosis)	Study Type	Control (n)	GDM (n)	Sample type	Proteomic platform	NOS
Ai, T., et al (1)	2015	China	Asia	24 weeks	2	IADPSG	75 g	24 weeks	2	case-control	10	11	serum	MALDI-TOF MS	8
Boisvert, MR., et al (2)	2010	Canada	North America	12-20 weeks	2	ADA	75 g	12-20 weeks (mean 15 weeks)	2	case-control	26	26	amniotic Fluid	QTOF-MS	9
Boyle, KE., et al (3)	2014	America	North America	37 weeks	2	CC	100 g	24-28 weeks	2	case-control	6	6	rectus abdominus skeletal muscle tissue	LTQ-FTICR MS	8
Fruscalzo, A., et al (4)	2015	Italy	Europe	11-14 weeks	1-2	IADPSG	75 g	24-28 weeks	2	case-control	44	32	serum	MALDI-TOF MS	8
Guo, Y., et al (5)	2018	China	Asia	15-20 weeks and 24-28 weeks	2	IADPSG	75 g	24 to 28 weeks	2	case-control and longitudinal	16	16	urine	iTRAQ LC-MS/MS	8
Hajduk, J., et al (6)	2015	Poland	Europe	24 to 28 weeks	2	Polish GS standard	75 g	24 to 28 weeks	2	case-control	13	18	plasma	MALDI-TOF MS	9
Ilyas, S., et al (7)	2020	Pakistan	Asia	16-20 weeks and 24-28 weeks	2	ACOG	100 g	24 to 28 weeks	2	case-control and longitudinal	84	84	plasma	2-DE-LC-MS/MS	8

Jayabalan, N., et al (8)	2019	Australia	Australia	22-28 weeks	2	ADIPS and WHO	75 g	24 to 28 weeks	2	case-control	11	11	plasma	SWATH-MS	8
Kim, SM., et al (9)	2012	Korea	Asia	16-20 weeks and 24-28 weeks	2	ACOG	100 g	24 to 28 weeks	2	case-control and longitudinal	12	12	peripheral plasma	SELDI-TOF MS	9
Kopylov, A.T., et al (10),	2020	Russia	Europe	23 to 28 weeks	NA	IADPSG and adopted Russian AOG	75 g	23 to 28 weeks	2	case-control	36	80	plasma	LC-MS/MS	8
Li, J., et al (11)	2021	China	Asia	NA	NA	IADPSG	75g	24 to 28 weeks	2	case-control	8	12	serum	2-DE-LC-MS/MS	8
Liao, Y., et al (12)	2018	China	Asia	*NA	NA	IADPSG	75 g	24 to 28 weeks	2	case-control	4	4	peripheral plasma; umbilical venous plasma	iTRAQ LC-MS/MS	9
Liu, B., et al (13)	2018	China	Asia	NA	NA	ADA	75 g	24 to 28 weeks	2	case-control	25	25	placenta villi	2-DE-MALDI-TOF/TOF	8
Liu, F., et al (14)	2016	China	Asia	*NA	NA	IADPSG and ADA	75 g	24 to 28 weeks	2	case-control	30	30	umbilical cord plasma	LC-MS/MS	9
Liu, X., et al (15)	2020	China	Asia	16-18 weeks	2	Chinese PLA General Hospital	NA	24 to 28 weeks	2	case-control and longitudinal	22	22	plasma	LC-MS/MS	9
Ma, Y., et al (16)	2016	China	Asia	*NA	NA	IADPSG	75 g	24-28 weeks	2	case-control	6	6	omental adipose tissue	LC-MS/MS	8
Mavreli, D., et al (17)	2020	Greece	Europe	11-13 weeks	1	ACOG	75 g	24 to 28 weeks	2	case-control and longitudinal	5	5	plasma	TMT LC-MS/MS	9

Miao, Z., et al (18)	2016	China	Asia	NA	NA	ADA	75 g	24 to 28 weeks	2	case-control and longitudinal	30	30	umbilical vein blood plasma	TMT LC-MS/MS	9
Ramachandrarao, SP., et al (19)	2016	America	North America	20 weeks	2-3	The California Sweet Success Program	NA	at first or second trimester	1 or 2	case-control and longitudinal	10	8	urine exosomes	LC-MS/MS	8
Ravnsborg, T., et al (20)	2016	Denmark	Europe	8 - 13 weeks	1	the Danish GDM guideline	NA	NA	NA	case-control	208	199	serum	SIL peptide LC-MS/MS	9
Ravnsborg, T., et al (21)	2019	Denmark	Europe	8 - 13 weeks	1	the Danish GDM guideline	NA	NA	NA	case-control	30	30	peripheral serum	TMT LC-MS/MS	9
Shen, L., et al (22)	2019	China	Asia	12–16 weeks and 24-28 weeks	1	IADPSG	75 g	24-28 weeks	2	case-control and longitudinal	30	30	peripheral serum	iTRAQ LC-MS/MS	8
Zhao, C., et al (23)	2015	China	Asia	16-18 weeks	2	Hospital of Nanjing	75 g	24-28 weeks	2	case-control	20	20	peripheral plasma	TMT LC-MS/MS	9
Zhao, D., et al (24)	2017	China	Asia	12-16 weeks	2	IADPSG	75 g	24-28 weeks	2	case-control and longitudinal	10	10	serum	iTRAQ LC-MS/MS	8

Note: Abbreviations IADPSG (International Association of the Diabetes and Pregnancy Study Groups), ADA (the American Diabetes Association), WHO (World Health Organization), ACOG (The American College of Obstetricians and Gynecologists), CC (Carpenter-Coustan Criteria), ADIPS (Australasian Diabetes in Pregnancy Society), NA (data not available), NOS (Newcastle-Ottawa Scale), MS (Mass Spectrometer), MALDI (Matrix Assisted Laser Desorption/Ionization), TOF (Time of Flight), LTQ (Hybrid Linear Ion Trap), FTICR (Fourier Transform Ion Cyclotron Resonance), iTRAQ (Isobaric Tagging for Relative and Absolute Quantification), LC (Liquid Chromatography), 2DE (Two-Dimensional Gel Electrophoresis), SWATH (Sequential Window Acquisition of All Theoretical), SELDI (Surface-Enhanced Laser Desorption/Ionization), SIL (Stable Isotope Labeled)

Supplementary Table S2. Diagnostic criteria using the OGTT for GDM guidelines used in included studies

Diagnostic Criteria	Guidelines	Glucose Dose	Fasting Glucose mg/dl (mmol/l)	1 hour mg/dl (mmol/l)	2 hours mg/dl (mmol/l)	3 hours mg/dl (mmol/l)	
1	ADA(75)	75g	95(5.3)	180(10.0)	155(8.6)		2 or more
2	ADIPS	75g	99(5.5)		144(8.0)		1 or more
3	EASD	75g	108(6.0)		162(9.0)		1 or more
4	IADPSG	75g	92(5.1)	180(10.0)	153(8.5)		1 or more
5	WHO 1999 /Modified	75g	126(7.0)/110(6.1)		140(7.8)		1 or more
6	ACOG/ C&C/ADA(100)	100g	95(5.3)	180(10.0)	155(8.6)	140(7.8)	2 or more
7.	Polish GS standard	75g	92(5.1)	180(10.0)	153(8.5)		1 or more
8.	Adopted Russian AOG	75g	104.4 (5.8)	176.4(9.8)			1 or more
9.	Chinese PLA General Hospital	75g	92(5.1)	180(10.0)	153(8.5)		1 or more
10.	The California Sweet Success program	75g	92(5.1)	180(10.0)	153(8.5)		1 or more
11.	The Danish GDM	75g	110(6.1)		140(7.8)		1 or more
12.	Hospital of Nanjing	75g	92(5.1)	180(10.0)	153(8.5)		1 or more

Supplementary Table S3. Study quality assessment using Newcastle-Ottawa scale for case-control studies

First author, year (reference)	**Selection**				**Comparability**	**Outcome**			**Total score**
	Adequate case definition	Representativeness of cases	Selection of controls	Definition of controls	Comparability of cases-controls	Ascertainment of exposure	Same method of ascertainment	Non-response rate	
Ai, T., 2015	*	*	*	*	*	*	*	*	8
Boisvert, MR., 2010	*	*	*	*	**	*	*	*	9
Boyle, KE., 2014	*	*	*	*	*	*	*	*	8
Fruscalzo, A., 2015	*	*	*	*	*	*	*	*	8
Guo, Y., 2018	*	*	*	*	*	*	*	*	8
Hajduk, J., 2015	*	*	*	*	**	*	*	*	9
Ilyas, S., 2020	*	*	*	*	*	*	*	*	8
Jayabalan, N., 2019	*	*	*	*	*	*	*	*	8
Kim, SM., 2012	*	*	*	*	**	*	*	*	9
Kopylov, AT., 2020	*	*	*	*	*	*	*	*	8
Li, J., 2021	*	*	*	*	*	*	*	*	8
Liao, Y., 2018	*	*	*	*	**	*	*	*	9
Liu, B., 2012	*	*	*	*	*	*	*	*	8

Liu, F., 2016	*	*	*	*	**	*	*	*	9
Liu, X., 2020	*	*	*	*	**	*	*	*	9
Ma, Y., 2016	*	*	*	*	*	*	*	*	8
Mavreli, D., 2020	*	*	*	*	**	*	*	*	9
Miao, Z., 2016	*	*	*	*	**	*	*	*	9
Ramachandrarao, SP., 2016	*	*	*	*	*	*	*	*	8
Ravnsborg, T., 2016	*	*	*	*	**	*	*	*	9
Ravnsborg, T., 2019	*	*	*	*	**	*	*	*	9
Shen, L., 2019	*	*	*	*	*	*	*	*	8
Zhao, C., 2015	*	*	*	*	**	*	*	*	9
Zhao, D., 2017	*	*	*	*	*	*	*	*	8

Note: Each asterisk represents if individual criterion within the subsection was fulfilled. A study can be awarded a maximum of one star (*) for each numbered item within the Selection and Exposure categories. A maximum of two stars can be given for Comparability.

Thresholds for converting the Newcastle-Ottawa scales to AHRQ standards (good, fair, and poor):

Good quality: 3 or 4 stars in selection domain AND 1 or 2 stars in comparability domain AND 2 or 3 stars in outcome/exposure domain

Fair quality: 2 stars in selection domain AND 1 or 2 stars in comparability domain AND 2 or 3 stars in outcome/exposure domain

Poor quality: 0 or 1 star in selection domain OR 0 stars in comparability domain OR 0 or 1 stars in outcome/exposure domain

Supplementary Table S5. Grouping of replicable CBs by sample type

Sample Type	Replicable CB	Regulation	Total cohort	Reference (First name, year)		
**A) Plasma**						
2 cohorts	C-reactive protein	up	3	Liu, X. 2020	Zhao, C. 2015	
2 cohorts	Sex hormone-binding globulin	down	3	Liu, X. 2020	Zhao, C. 2015	
2 cohorts	Pappalysin 1	down	2	Jayabalan, N. 2019	Zhao, C. 2015	
1 cohort	Complement component C9	down	3	Miao, Z. 2016		
1 cohort	Ig mu chain C region (Immunoglobulin heavy constant mu)	down	3	Liu, X. 2020		
1 cohort	Proteoglycan 4	up	3	Liu, X. 2020		
1 cohort	Secreted phosphoprotein 24	down	3	Liu, X. 2020		
1 cohort	Alpha-1-antitrypsin	down	2	Zhao, C. 2015		
1 cohort	Apolipoprotein C-III	up	2	Kim, SM. 2012		
1 cohort	Serum amyloid P-component	up	2	Liu, X. 2020		
**B) Serum**						
3 cohorts	Apolipoprotein A-V	up	3	Shen, L. 2019	Zhao, D. 2017	Li, J. 2021
3 cohorts	Apolipoprotein E	up	3	Shen, L. 2019	Zhao, D. 2017	Li, J. 2021
3 cohorts	Gelsolin	down	3	Shen, L. 2019	Zhao, D. 2017	Li, J. 2021
2 cohort	Alpha-2-macroglobulin	down	2	Ravnsborg, T. 2016	Li, J. 2021	
2 cohorts	Antithrombin-III	down	2	Ravnsborg, T. 2019	Zhao, D. 2017	
2 cohort	Apolipoprotein C-III	up	3	Shen, L. 2019	Li, J. 2021	
2 cohort	Apolipoprotein L1	up	2	Ravnsborg, T. 2016	Li, J. 2021	
2 cohorts	Complement component C9	down	3	Shen, L. 2019	Zhao, D. 2017	
2 cohorts	Ig mu chain C region (Immunoglobulin heavy constant mu)	down	3	Shen, L. 2019	Zhao, D. 2017	
2 cohorts	Proteoglycan 4	up	3	Shen, L. 2019	Zhao, D. 2017	
2 cohorts	Secreted phosphoprotein 24	down	3	Ravnsborg, T. 2019	Shen, L. 2019	

2 cohorts	C4b-binding protein alpha chain	down	2	Shen, L. 2019	Zhao, D. 2017
2 cohorts	Coagulation factor IX	up	2	Shen, L. 2019	Zhao, D. 2017
2 cohorts	Coagulation factor X	up	2	Shen, L. 2019	Zhao, D. 2017
2 cohorts	Coagulation factor XII	up	2	Shen, L. 2019	Zhao, D. 2017
2 cohorts	Complement C1s subcomponent	up	2	Shen, L. 2019	Zhao, D. 2017
2 cohorts	Complement component C6	down	2	Shen, L. 2019	Zhao, D. 2017
2 cohorts	Complement component C7	down	2	Shen, L. 2019	Zhao, D. 2017
2 cohorts	Complement component C8 beta chain	down	2	Shen, L. 2019	Zhao, D. 2017
2 cohorts	Complement component C8 gamma chain	down	2	Shen, L. 2019	Zhao, D. 2017
2 cohorts	Complement factor H	down	2	Shen, L. 2019	Zhao, D. 2017
2 cohorts	Endoplasmin	down	2	Shen, L. 2019	Zhao, D. 2017
2 cohorts	Glyceraldehyde-3-phosphate dehydrogenase	up	2	Shen, L. 2019	Zhao, D. 2017
2 cohorts	Insulin-like growth factor-binding protein 5	up	2	Shen, L. 2019	Zhao, D. 2017
2 cohorts	Serum paraoxonase/arylesterase 1	up	2	Shen, L. 2019	Zhao, D. 2017
1 cohort	Alpha-1-antitrypsin	down	2	Shen, L. 2019	
1 cohort	C-reactive protein	up	3	Shen, L. 2019	
1 cohort	Haptoglobin	up	2	Ravnsborg, T. 2016	
1 cohort	Serum amyloid P-component	up	2	Ravnsborg, T. 2019	
1 cohort	Sex hormone-binding globulin	down	3	*Ravnsborg, T. 2016 and 2019	
**C) Urine exosomes**					
1 cohort	Haptoglobin	up	2	Ramachandrarao, SP. 2016	

Supplementary Table S6. Grouping of replicable CBs by proteomic platform

Replicable CB	Regulation	Total cohort	Proteomic platform MS-based (number of cohort)						Reference (First name, year)		
			A) Gel image	B) Spectra intensity							
				i) Lable free			ii) Chemical labeling				
			2-DE-MS	MALDI/SELDI-MS	LC-MS/MS	SWATH-MS	TMT LC-MS/MS	iTRAQ LC-MS/MS			
Complement component C9	down	3					1	2	Miao, Z. 2016	Shen, L. 2019	Zhao, D. 2017
C-reactive protein	up	3			1		1	1	Liu, X. 2020	Shen, L. 2019	Zhao, C. 2015
Ig mu chain C region	down	3			1			2	Liu, X. 2020	Shen, L. 2019	Zhao, D. 2017
Proteoglycan 4	up	3			1			2	Liu, X. 2020	Shen, L. 2019	Zhao, D. 2017
Secreted phosphoprotein 24	down	3			1		1	1	Liu, X. 2020	Ravnsborg, T. 2019	Shen, L. 2019
Sex hormone-binding globulin	down	3			1		2		Liu, X. 2020	*Ravnsborg, T. 2016 and 2019	Zhao, C. 2015
Alpha-1-antitrypsin	down	2					1	1	Shen, L. 2019	Zhao, C. 2015	
Alpha-2-macroglobulin	down	2	1				1		Ravnsborg, T. 2016	Li, J. 2021	
Antithrombin-III	down	2					1	1	Ravnsborg, T. 2019	Zhao, D. 2017	
Apolipoprotein A-V	up	3	1					2	Shen, L. 2019	Zhao, D. 2017	Li, J. 2021
Apolipoprotein C-III	up	3	1	1				1	Kim, SM. 2012	Shen, L. 2019	Li, J. 2021
Apolipoprotein E	up	3	1					2	Shen, L. 2019	Zhao, D. 2017	Li, J. 2021
Apolipoprotein L1	up	2	1				1		Ravnsborg, T. 2016	Li, J. 2021	

C4b-binding protein alpha chain	down	2					2	Shen, L. 2019	Zhao, D. 2017	
Coagulation factor IX	up	2					2	Shen, L. 2019	Zhao, D. 2017	
Coagulation factor X	up	2					2	Shen, L. 2019	Zhao, D. 2017	
Coagulation factor XII	up	2					2	Shen, L. 2019	Zhao, D. 2017	
Complement C1s subcomponent	up	2					2	Shen, L. 2019	Zhao, D. 2017	
Complement component C6	down	2					2	Shen, L. 2019	Zhao, D. 2017	
Complement component C7	down	2					2	Shen, L. 2019	Zhao, D. 2017	
Complement component C8 beta chain	down	2					2	Shen, L. 2019	Zhao, D. 2017	
Complement component C8 gamma chain	down	2					2	Shen, L. 2019	Zhao, D. 2017	
Complement factor H	down	2					2	Shen, L. 2019	Zhao, D. 2017	
Endoplasmin	down	2					2	Shen, L. 2019	Zhao, D. 2017	
Gelsolin	down	3	1				2	Shen, L. 2019	Zhao, D. 2017	Li, J. 2021
Glyceraldehyde-3-phosphate dehydrogenase	up	2					2	Shen, L. 2019	Zhao, D. 2017	
Haptoglobin	up	2		1		1		Ramachandrarao, SP. 2016	Ravnsborg, T. 2016	
Insulin-like growth factor-binding protein 5	up	2					2	Shen, L. 2019	Zhao, D. 2017	
Pappalysin1	down	2			1	1		Jayabalan, N. 2019	Zhao, C. 2015	
Serum amyloid P-component	up	2		1		1		Liu, X. 2020	Ravnsborg, T. 2019	
Serum paraoxonase/arylesterase 1	up	2					2	Shen, L. 2019	Zhao, D. 2017	

Supplementary Table S7. Grouping of replicable CBs by continent

Continent	Replicable CB	Country	Regulation	Total cohorts	Reference (First name, year)		
**A) Asia**							
3 cohorts	Complement component C9	China	down	3	Miao, Z. 2017	Shen, L. 2020	Zhao, D. 2018
3 cohorts	C-reactive protein	China	up	3	Liu, X. 2020	Shen, L. 2019	Zhao, C. 2015
3 cohorts	Ig mu chain C region (Immunoglobulin heavy constant mu)	China	down	3	Liu, X. 2020	Shen, L. 2019	Zhao, D. 2017
3 cohorts	Proteoglycan 4	China	up	3	Liu, X. 2020	Shen, L. 2019	Zhao, D. 2017
2 cohorts	Secreted phosphoprotein 24	China	down	3	Liu, X. 2020	Shen, L. 2019	
2 cohorts	Sex hormone-binding globulin	China	down	3	Liu, X. 2020	Zhao, C. 2015	
2 cohorts	Alpha-1-antitrypsin	China	down	2	Shen, L. 2019	Zhao, C. 2015	
3 cohorts	Apolipoprotein A-V	China	up	3	Shen, L. 2019	Zhao, D. 2017	Li, J. 2021
3 cohorts	Apolipoprotein C-III	Korea and China	up	3	Kim, SM. 2012	Shen, L. 2019	Li, J. 2021
3 cohorts	Apolipoprotein E	China	up	3	Shen, L. 2019	Zhao, D. 2017	Li, J. 2021
3 cohorts	Gelsolin	China	down	3	Shen, L. 2019	Zhao, D. 2017	Li, J. 2021
2 cohorts	C4b-binding protein alpha chain	China	down	2	Shen, L. 2019	Zhao, D. 2017	
2 cohorts	Coagulation factor IX	China	up	2	Shen, L. 2019	Zhao, D. 2017	
2 cohorts	Coagulation factor X	China	up	2	Shen, L. 2019	Zhao, D. 2017	
2 cohorts	Coagulation factor XII	China	up	2	Shen, L. 2019	Zhao, D. 2017	
2 cohorts	Complement C1s subcomponent	China	up	2	Shen, L. 2019	Zhao, D. 2017	
2 cohorts	Complement component C6	China	down	2	Shen, L. 2019	Zhao, D. 2017	
2 cohorts	Complement component C7	China	down	2	Shen, L. 2019	Zhao, D. 2017	

2 cohorts	Complement component C8 beta chain	China	down	2	Shen, L. 2019	Zhao, D. 2017
2 cohorts	Complement component C8 gamma chain	China	down	2	Shen, L. 2019	Zhao, D. 2017
2 cohorts	Complement factor H	China	down	2	Shen, L. 2019	Zhao, D. 2017
2 cohorts	Endoplasmin	China	down	2	Shen, L. 2019	Zhao, D. 2017
2 cohorts	Glyceraldehyde-3-phosphate dehydrogenase	China	up	2	Shen, L. 2019	Zhao, D. 2017
2 cohorts	Insulin-like growth factor-binding protein 5	China	up	2	Shen, L. 2019	Zhao, D. 2017
2 cohorts	Serum paraoxonase/arylesterase 1	China	up	2	Shen, L. 2019	Zhao, D. 2017
1 cohort	Alpha-2-macroglobulin	China	down	2	Li, J. 2021	
1 cohort	Antithrombin-III	China	down	2	Zhao, D. 2017	
1 cohort	Apolipoprotein L1	China	up	2	Li, J. 2021	
1 cohort	Pappalysin1	China	down	2	Zhao, C. 2015	
1 cohort	Serum amyloid P-component	China	up	2	Liu, X. 2020	
**B) Europe**						
1 cohort	Alpha-2-macroglobulin	Denmark	down	2	Ravnsborg, T. 2016	
1 cohort	Antithrombin-III	Denmark	down	2	Ravnsborg, T. 2019	
1 cohort	Apolipoprotein L1	Denmark	up	2	Ravnsborg, T. 2016	
1 cohort	Secreted phosphoprotein 24	Denmark	down	3	Ravnsborg, T. 2019	
1 cohort	Sex hormone-binding globulin	Denmark	down	3	*Ravnsborg, T. 2016 and 2019 (same cohort)	
1 cohort	Haptoglobin	Denmark	up	2	Ravnsborg, T. 2016	
1 cohort	Serum amyloid P-component	Denmark	up	2	Ravnsborg, T. 2019	

**C) North America**					
1 cohort	Haptoglobin	America	up	2	Ramachandrarao, SP. 2016
**D) Australia**					
1 cohort	Pappalysin 1	Australia	down	2	Jayabalan, N. 2019

Supplementary Table S8. DAVID functional annotation chart analysis representing pathways in KEGG terms; analysis was conducted for consistent regulation of CBs in 2 or more independent cohorts (see Supplementary Table S1 for UniProt ID of all CBs)

KEGG pathway term	List of proteins	Percentage, %	Modified Fisher Exact p-value/EASE score	Fold enrichment	Bonferroni corrected p-value < 0.05
Complement and coagulation cascades	alpha-2-macroglobulin, coagulation factor IX(F9), coagulation factor X(F10), coagulation factor XII(F12), complement C1s(C1S), complement C6(C6), complement C7(C7), complement C8 beta chain(C8B), complement C8 gamma chain(C8G), complement C9(C9)	43.3	8.1E-23	68.2	1.5E-19
Systemic lupus erythematosus	complement C1s(C1S), complement C6(C6), complement C7(C7), complement C8 beta chain(C8B), complement C8 gamma chain(C8G), complement C9(C9)	20.0	1.1E-06	16.2	4.2E-04
Prion diseases	complement C6(C6), complement C7(C7), complement C8 beta chain(C8B), complement C8 gamma chain(C8G), complement C9(C9)	16.7	2.4E-08	53.2	3.3E-05
Amoebiasis	complement C8 beta chain(C8B), complement C8 gamma chain(C8G), complement C9(C9)	10.0	2.9E-03	10.2	5.1E-01

Supplementary Table S9. DAVID functional annotation chart analysis representing pathways in KEGG terms on 27 early pregnancy blood CBs for GDM excluding pilot studies; analysis was conducted for consistent regulation of CBs in 2 or more independent cohorts

KEGG pathway term	List of proteins	Percentage, %	Modified Fisher Exact p-value/EASE score	Fold enrichment	Bonferroni corrected p-value < 0.05
Complement and coagulation cascades	coagulation factor V (F5), coagulation factor IX (F9), coagulation factor X (F10), coagulation factor XII (F12), complement C1s (C1S), complement component C6 (C6), complement component C7 (C7), complement component C8 beta chain (C8B), complement component C8 gamma chain (C8G), complement component C9 (C9), fibrinogen alpha chain (FGA),	53.8	1.9E-25	74.2	4.7E-22
Coronavirus disease - COVID-19	complement C1s (C1S), complement component C6 (C6), complement component C7 (C7), complement component C8 beta chain (C8B), complement component C8 gamma chain (C8G), complement component C9 (C9), fibrinogen alpha chain (FGA)	30.8	1.4E-8	15.5	6.3E-6
Systemic lupus erythematosus	complement C1s (C1S), complement component C6 (C6), complement component C7 (C7), complement component C8 beta chain (C8B), complement component C8 gamma chain (C8G), complement component C9 (C9)	23.1	3.1E-7	19.9	1.9E-4
Prion diseases	complement component C6 (C6), complement component C7 (C7), complement component C8 beta chain (C8B), complement component C8 gamma chain (C8G), complement component C9 (C9)	19.2	2.5E-4	8.3	5.9E-2
Amoebiasis	complement component C8 beta chain (C8B), complement component C8 gamma chain (C8G), complement component C9 (C9)	11.5	1.4E-3	13.3	4.2E-1
